# Supplementary material for: Anticancer and Antiphytopathogenic Activity of Fluorinated Isatins and Their Water-Soluble Hydrazone Derivatives
Source: Int J Mol Sci. 2023 Oct 12;24(20):15119. doi: 10.3390/ijms242015119 (PMC10607100; doi:10.3390/ijms242015119)
Supplement: Supplementary file 1 [file ijms-24-15119-s001.zip › ijms-2569665-supplementary.pdf]

SUPPLEMENTARY MATERIALS

Anticancer and Antiphytopathogenic Activity of Fluorinated Isatins and Their Water-Soluble Hydrazone Derivatives

Andrei V. Bogdanov [1,*,†], Margarita Neganova [1,2,†], Alexandra Voloshina [1], Anna Lyubina [1], Syumbelya Amerhanova [1], Igor A. Litvinov [1], Olga Tsivileva [3], Nurgali Akylbekov [4], Rakhmetulla Zhapparbergenov [4,*], Zulfiia Valiullina [5], Alexandr V. Samorodov [5] and Igor Alabugin [1,6]

[1] Arbuzov Institute of Organic and Physical Chemistry, FRC Kazan Scientific Center, Russian Academy of Sciences, Akad. Arbuzov St. 8, Kazan 420088, Russia; neganovam@ipac.ac.ru (M.N.);
microbi@iopc.ru (A.V.); aplyubina@gmail.com (A.L.); syumbelya07@mail.ru (S.A.); litvinov@iopc.ru (I.A.L.); ialabugin@gmail.com (I.A.)
[2] Institute of Physiologically Active Compounds at Federal Research Center of Problems of Chemical Physics and Medicinal Chemistry, Russian Academy of Sciences, Severnij Pr. 1, Chernogolovka 142432, Russia
[3] Institute of Biochemistry and Physiology of Plants and Microorganisms, Saratov Scientific Centre of the Russian Academy of Sciences, Entuziastov Ave. 13, Saratov 410049, Russia; tsivileva_o@ibppm.ru
[4] Laboratory of Engineering Profile "Physical and Chemical Methods of Analysis", Korkyt Ata Kyzylorda University, Aitekebie Str. 29A, Kyzylorda 120014, Kazakhstan; nurgali_089@mail.ru
[5] Department of pharmacology, Bashkir State Medical University, Lenin St. 8, Ufa 450008, Russia; z_suleimanova@mail.ru (Z.V.); avsamorodov@gmail.com (A.V.S.)
[6] Department of Chemistry and Biochemistry, Florida State University, 95 Chieftan Way, Tallahassee, FL 32306-4390, USA
* Correspondence: abogdanov@inbox.ru (A.V.B.); ulagat-91@mail.ru (R.Z.); Tel.: +7-843-272-7384 (A.V.B.); +7-724-223-1041 (R.Z.)
† These authors contributed equally to this work.

Contents

X-Ray data 2

Antiphytopathogenic activity data 3

Copies of NMR spectra 8

X-Ray data

Table S1 The detailed x-ray data for the compounds **3f** and **4c**

**Compound**	**3f**	**4c**
Chemical formula	$C_{15}H_5F_6NO_2$	$C_{19}H_{18}FNO_2$·
M_r	345.20	311.34
Crystal system	orthorhombic	orthorhombic
Space group	*Pna21 (No. 33)*	*Pbca (No. 61)*
Temperature (K)	121	121
a, *b*, *c* (Å)	7.9420(12<br>13.875(2)<br>11.771(2)	13.0749(10)<br>10.6137(9)<br>23.4703(17)
V (Å^3)	1297.1(3)	3257.1(4)
Z	4	8
D(calc) [g/cm^3]	1.768	
Radiation type	Mo *K*α	
μ (mm^{-1})	0.176	0.090
Crystal size (mm)	0.10 × 0.06 × 0.04	0.30 × 0.13 × 0.10
Diffractometer	Bruker D8 QUEST	Bruker D8 QUEST
T_{min}, T_{max}	0.6619 , 0.7241	0.3805, 0.7457
No. of measured reflections	5583	91130
No. of independent reflections	1438	4053
No. of observed [I > 2s(I)] reflections	1262	2419
R_{int}	0.049	0.236
R_1/ $wR(F^2)$, [I>2σ]	0.0276 / 0.0512	0.0562 / 0.1384
R_1/ $wR(F^2)$, (all data)	0.0375 / 0.0532	0.1083 / 0.1597
GOOF	1.009	0.0532
No. of reflections	1438	4053
No. of parameters	217	211
No. of restraints	1	0
Dρ$_{max}$, Dρ$_{min}$ (e Å^{-3})	0.11 / -0.14	0.31 / −0.27
**CCDC no**	2268269	2268273

A)

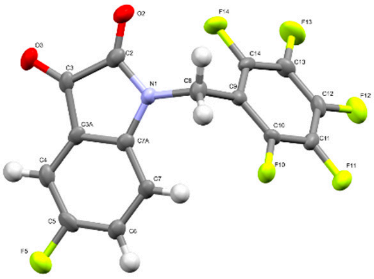

B)

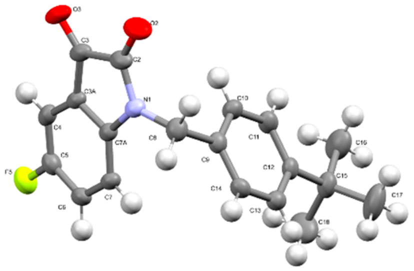

Figure S1 Molecular structure of compounds **3f** (A), and **4c** (B), ellipsoids are shown with 50% probability

According to X-Ray data (Figures S1a, b, Table S1) compounds **3f** and **4c** crystallize in different orthorhombic space groups: compound **3f** crystallizes in the non-centrosymmetric racemic space group $Pna2_1$, compound **4c** crystallizes in the centrosymmetric space group *Pbca*. Thus, both compounds in crystals exist in the form of true racemates. The conformation of the molecules in the crystals is practically the same: the isatin fragments are planar, the nitrogen atom N1 has planar trigonal coordination, and the benzyl substituents at the nitrogen atom are unfolded asymmetrically along the N–C8 and C8–C9 bonds. Torsion angles C2-N1-C8-C9 and N1-C8-C9-C10(C14) in the molecule **3f** -110.3(5) and -114.0(5)^{o}, in the molecule **4c** -109.5(2) and -132.4(2)^{o} respectively, that is, one of the hydrogen atoms at the C8 atom is practically in a eclipsed conformation with the plane of the benzene ring of the substituent. In this case, the main geometric parameters (bond lengths and bond angles) in molecules **3f** and **4c** are usual.

A)

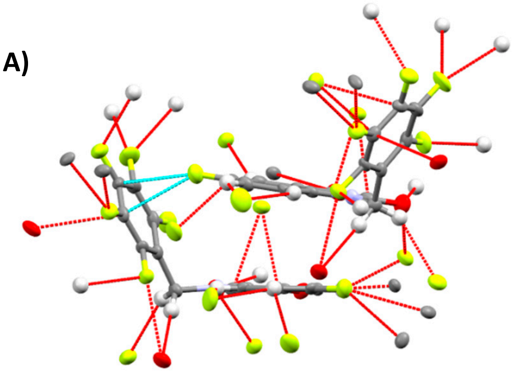

B)

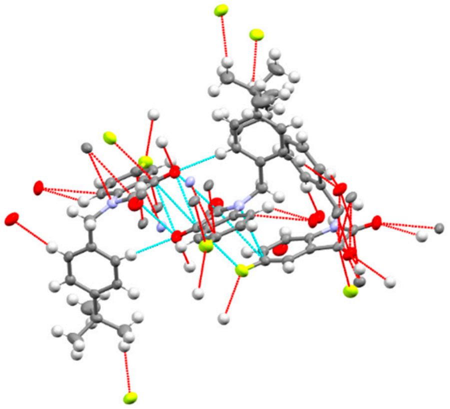

Figure S2 Fragments of crystal packing of the compound **3f** (A), and **4c** (B)

In crystal **3f**, a short intermolecular contact of the F…π type with the benzene ring of the pentafluoromethyl substituent of the neighboring molecule and multiple contacts of the C–H…F and C–H…O types are observed. In the **4c** crystal, contacts were found corresponding to π…π interactions of C=O bonds of isatin fragments of neighboring molecules, multiple C–H…O type contacts, and one C–H…F contact with the hydrogen atom of the methyl substituent. Nevertheless, the packing of crystals is quite dense; no free volumes were found in them available for small molecules (Figures S2a, b).

Antiphytopathogenic activity data

Table S2. Bactericidal activity of hydrazones (2 mM/L) studied*.

Compound	Bacterial phytopathogen				
	M. luteus	*P. atrosepticum*	*P. carotovorum subsp. carotovorum*	*Ps. fluorescens*	*X. campestris*
**5a**	4	6	3	4	3,5
**5b**	4	4	3	2,5	3
**5c**	4	5	5	5	7

**5d**	3	4	4	2	4
**5e**	6	4	5	7	5
**7a**	2	3	6	5	5
**7b**	7	4	5	4,5	7
**7c**	7	6	7	7	9
Norfloxacin, 500 μg/mL	7	7	8	8	7
sodium hypochlorite, 1000 μg/mL	2.5	4	3	4	4
Chlorohexidin, 500 μg/mL	5	4	5	4	5

* shows the values of the width of the inhibition zone (mm), averaged over the results of 3 experiments

Table S3. Comparative bactericidal activity of hydrazones **5a-e, 7a-c** (2 mM/L).

Bacterial test-system	Inhibition zone width (mm), not less						
	9	7	6	5	4	3	2
Micrococcus luteus B-109	–	**7b**, **7c**	**5e**	–	**5a**, **5b**	**5d**	**7a**
Pectobacterium atrosepticum 1043	–	–	**5a**, **7c**	**5c**	**5b**, **5d**	**7a**	–
Pectobacterium carotovorum subsp. *carotovorum* MI	–	**7c**	**7a**	**5c**, **5e**, **7b**	**5d**	**5a**, **5b**	–
Pseudomonas fluorescens EL-2.1	–	**5e**, **7c**	–	**5c**, **7a**	**5a**	–	**5b>5d**
Xanthomonas campestris B-610	**7c**	**5c**, **7b**	–	**5e**, **7a**	**5d**	**5a>5b**	–

Table S4. Fungicidal activity of hydrazones **5a-e, 7a-c** against *Fusarium oxysporum* IBPPM 543.

Compound	Concentration, ***C***, nmol/mL [μg/ml]	Inhibition value, ***I*** (%)*, at the age of the fungus (days)				EC_{50}, mcg/ml at day 5
		3	*5*	*7*	*9*	
**5a**	1 [0.44]	34	25	20	7	13.84
	5 [2.21]	39	31	23	18	
	20 [8.86]	41	45	42	23	
	40 [17.71]	52	54	51	36	
**5b**	1 [0.48]	9	10	9	0	14.84
	5 [2.39]	33	34	25	19	
	20 [9.55]	41	42	34	28	
	40 [19.09]	53	56	40	31	
	1 [0.49]	22	19	13	3	14.92

**5c**	5 [2.46]	40	36	28	13	
	20 [9.86]	49	45	35	21	
	40 [19.71]	57	55	52	51	
**5d**	1 [0.51]	9	10	10	0	17.35
	5 [2.57]	33	24	23	16	
	20 [10.30]	35	37	32	18	
	40 [20.59]	49	54	41	36	
**5e**	1 [0.49]	33	48	33	18	0.60
	3 [1.47]	66	67	65	64	
	20 [9.78]	69	72	69	67	
	30 [14.67]	75	76	72	71	
**7a**	1 [0.48]	9	4	8	0	7.25
	5 [2.42]	43	44	41	33	
	30 [14.50]	51	59	56	55	
	40 [19.33]	61	65	69	64	
**7b**	1 [0.53]	13	15	10	11	20.24
	5 [2.66]	32	29	20	13	
	20 [10.64]	35	35	30	25	
	40 [21.28]	55	52	43	39	
**7c**	1 [0.55]	22	21	15	10	13.90
	5 [2.74]	36	35	27	22	
	30 [16.41]	45	41	44	36	
	40 [21.88]	57	63	66	64	
Fludioxonil	[10.00]	18	23	6	4	83.33
	[15.00]	21	30	20	5	
	[30.00]	26	33	23	8	
	[60.00]	32	35	25	9	
N-cetylpyridinium chloride	[2.50]	0	10	0	0	141.1
	[5.00]	4	12	8	9	
	[25.00]	12	17	12	13	
	[50.00]	19	24	17	21	

* the average values are given; the standard deviation did not exceed 0.03 from the given value.

Table S5**.** Fungicidal activity of hydrazones **5a-e, 7a-c** against *Phytophthora cactorum* VKM F-985.

Compound	Concentration, ***C***, nmol/mL [μg/mL]	Inhibition value, ***I*** (%)*, at the age of the fungus (days)	EC_{50}, mcg/ml

						at day 5
		3	*5*	*7*	*9*	
**5a**	2 [0.89]	26	20	15	8	15.12
	3 [1.33]	32	32	28	19	
	27 [11.81]	40	44	31	25	
	40 [17.71]	47	53	43	36	
	50 [22.14]	52	62	45	38	
**5b**	1 [0.48]	18	26	11	6	18.19
	5 [2.39]	27	35	24	16	
	15 [7.16]	30	38	29	19	
	27 [12.73]	33	43	31	26	
	40 [19.09]	44	50	44	39	
**5c**	3 [1.48]	7	8	4	3	15.40
	7 [3.29]	13	21	14	12	
	27 [13.14]	36	38	29	18	
	40 [19.71]	55	64	42	36	
**5d**	1 [0.51]	15	12	10	8	24.74
	5 [2.57]	20	19	16	11	
	15 [7.72]	26	28	23	13	
	27 [13.73]	28	32	25	22	
	40 [20.59]	35	43	33	29	
**5e**	1 [0.49]	20	16	15	14	27.43
	3 [1.47]	23	19	17	17	
	7 [3.26]	25	30	23	19	
	27 [13.04]	28	35	26	21	
	40 [19.56]	35	38	29	26	
**7a**	3 [1.45]	5	12	4	4	22.87
	7 [3.22]	17	21	10	6	
	20 [9.67]	21	25	16	13	
	27 [12.89]	27	29	20	16	
	40 [19.33]	42	45	38	37	
**7b**	3 [1.60]	15	12	9	7	27.51
	7[3.55]	25	25	22	16	
	27 [14.19]	30	32	28	21	
	40 [21.28]	39	40	37	35	
**7c**	1 [0.55]	10	11	12	11	19.98
	3 [1.64]	20	18	17	16	

	7[3.65]	26	22	22	18	
	20 [10.94]	32	27	25	9	
	27 [14.59]	48	36	33	28	
	40 [21.88]	55	56	44	41	
Fludioxonil	[10.00]	36	38	35	9	29.51
	[15.00]	38	46	42	17	
	[30.00]	40	51	46	23	
	[60.00]	52	57	49	29	
N-cetylpyridinium chloride	[0.30]	0	0	0	3	34.79
	[1.00]	13	9	8	6	
	[2.50]	19	19	8	8	
	[5.00]	25	24	17	17	
	[25.00]	38	58	52	45	
	[50.00]	50	64	60	56	

* the average values are given; the standard deviation did not exceed 0.03 from the given value.

Copies of NMR spectra

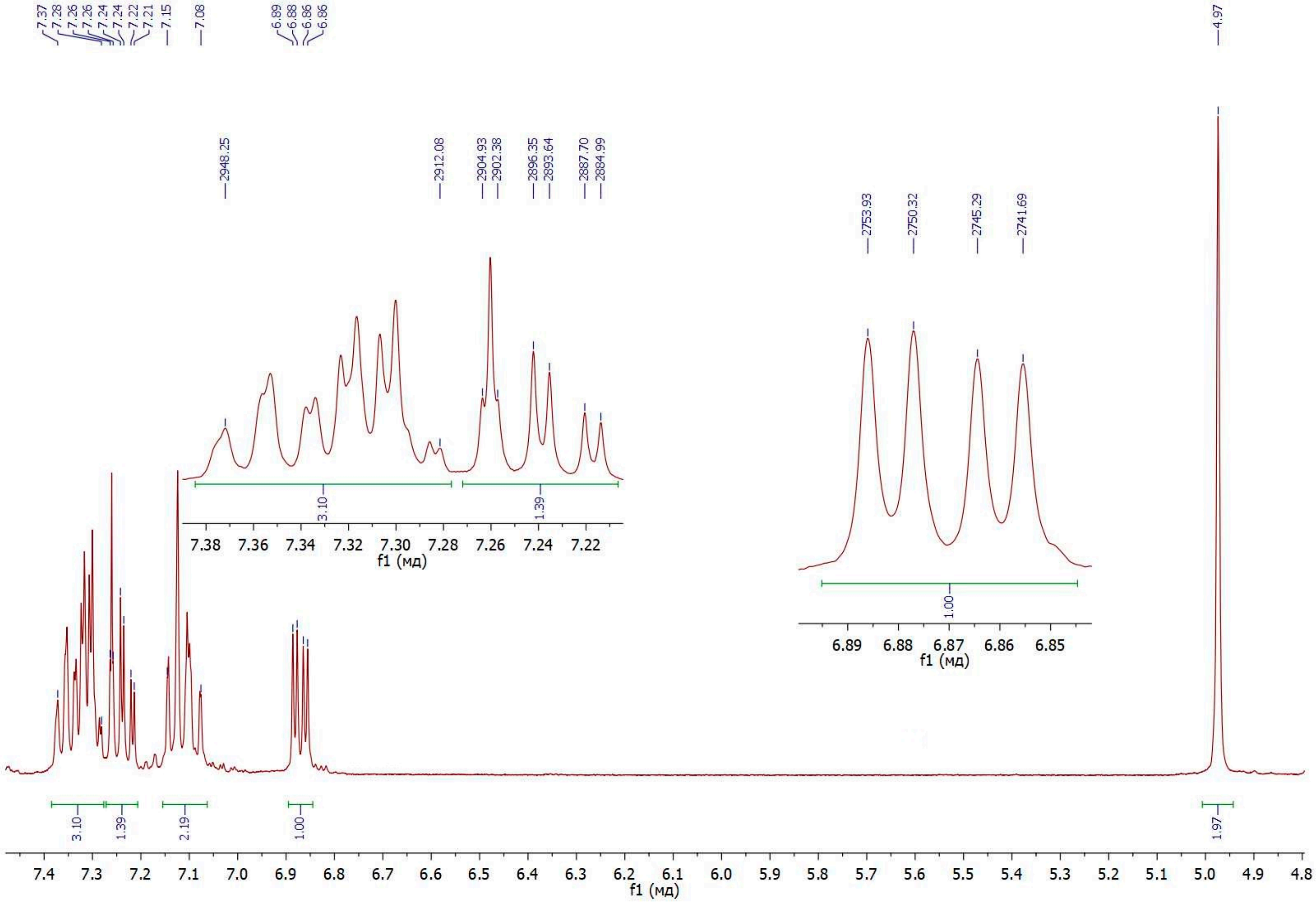

Figure S3. ^{1}H NMR spectrum of compound **3a** (600 MHz, $CDCl_3$)

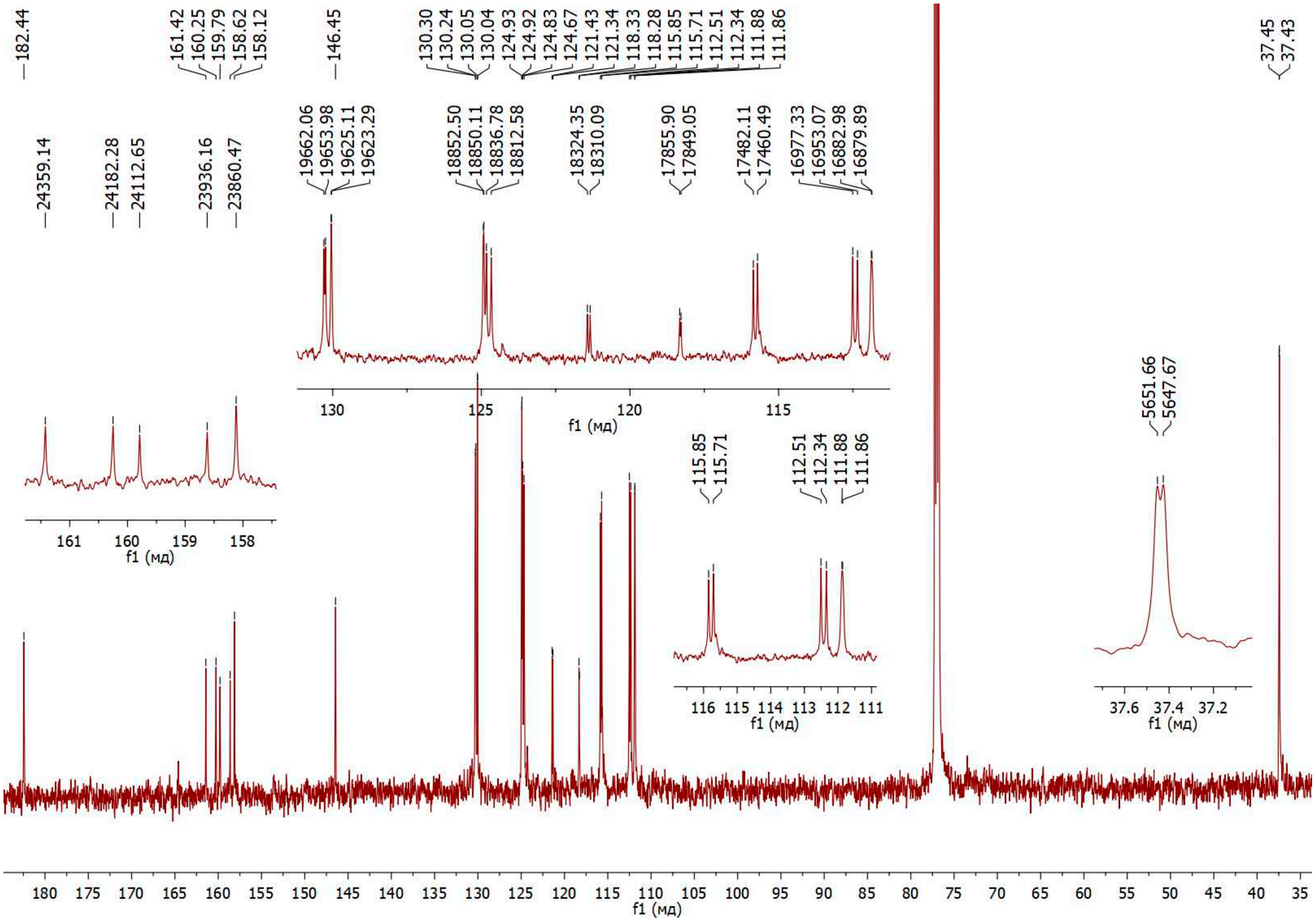

Figure S4. ^{13}C-{^{1}H} NMR spectrum of compound **3a** (151 MHz, $CDCl_3$)

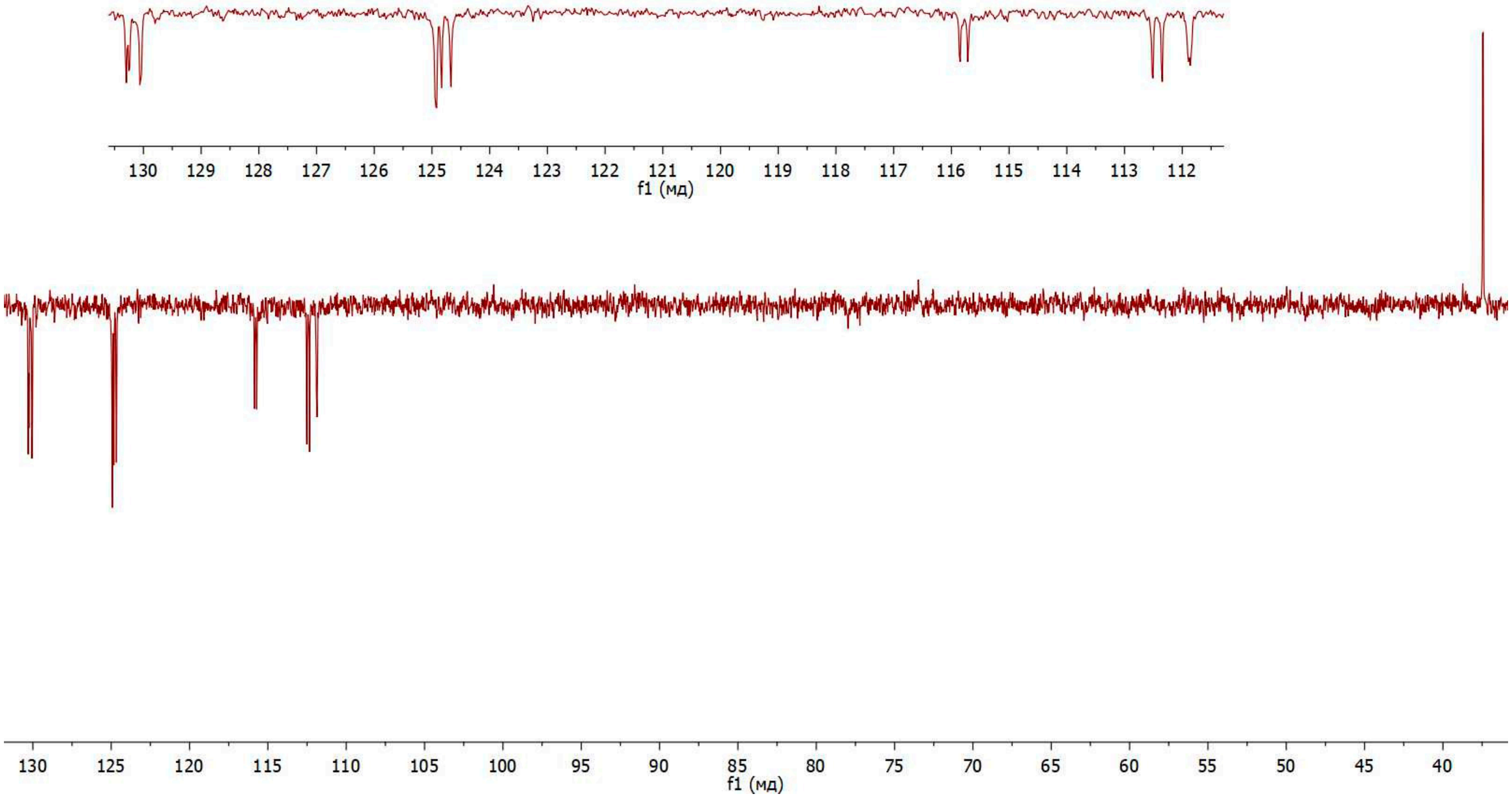

Figure S5. ^{13}C (dept) NMR spectrum of compound **3a** (151 MHz, $CDCl_3$)

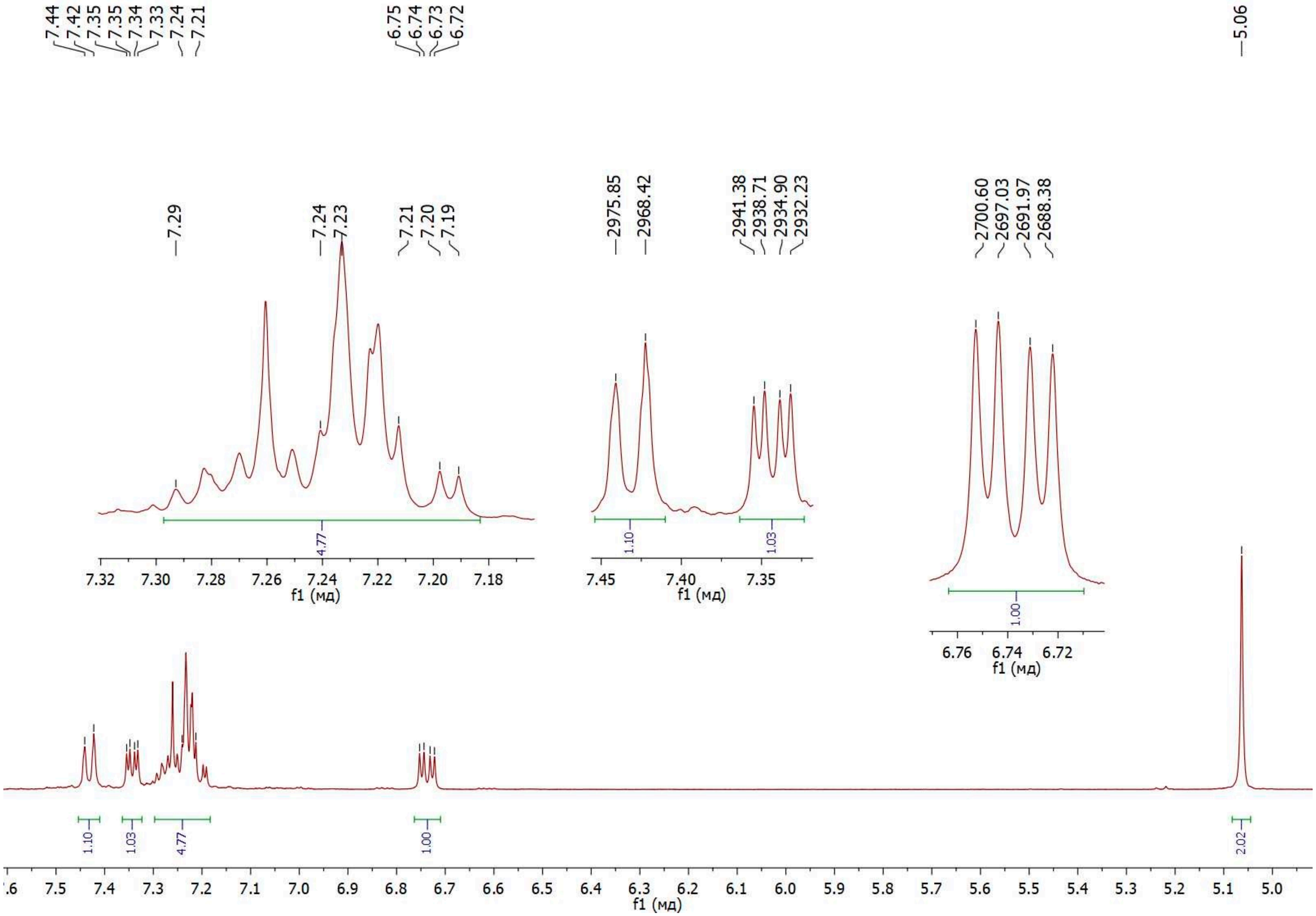

Figure S6. ^{1}H NMR spectrum of compound **3b** (600 MHz, $CDCl_3$)

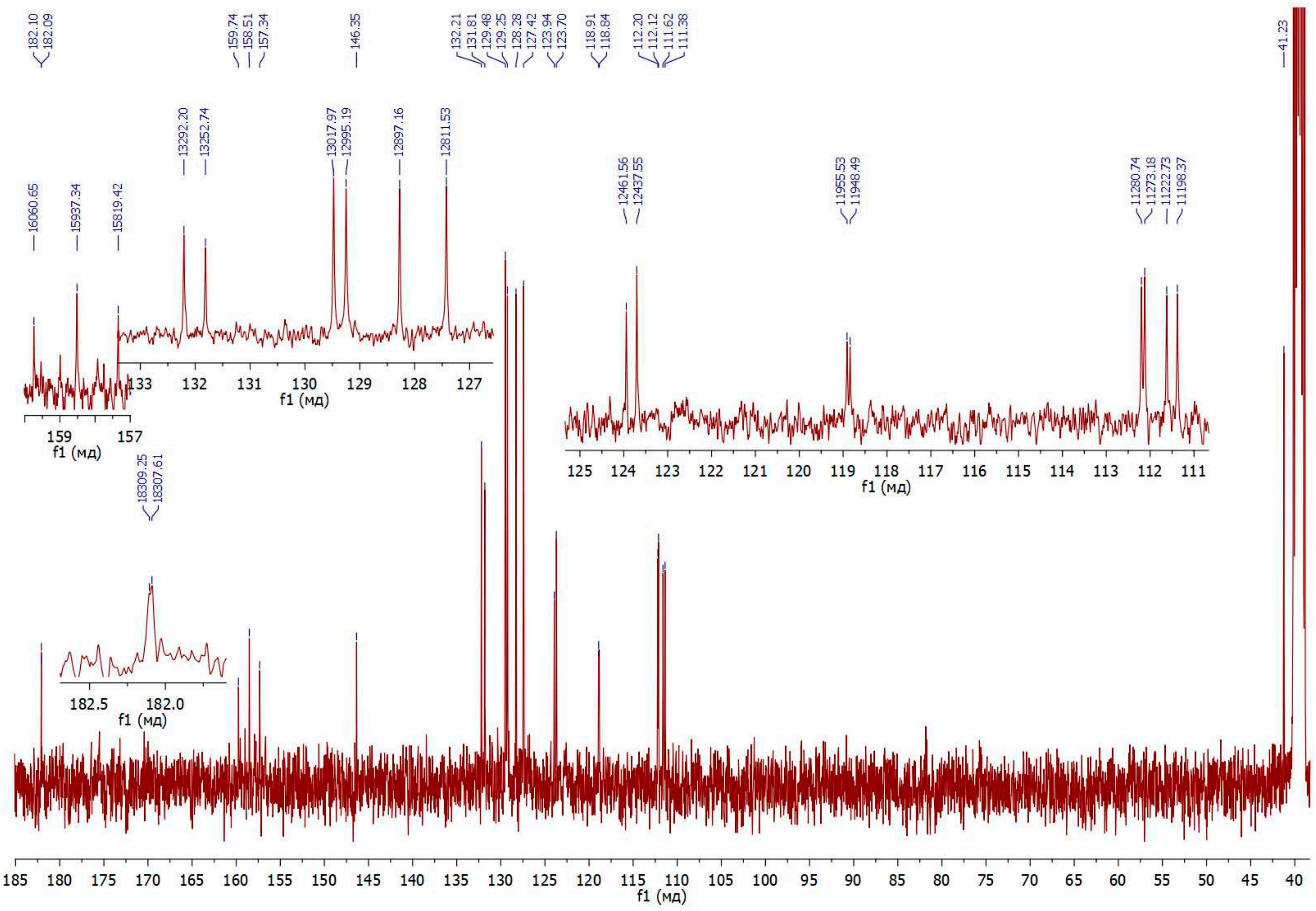

Figure S7. $^{13}C-\{^{1}H\}$ NMR spectrum of compound **3b** (126 MHz, DMSO-d_6)

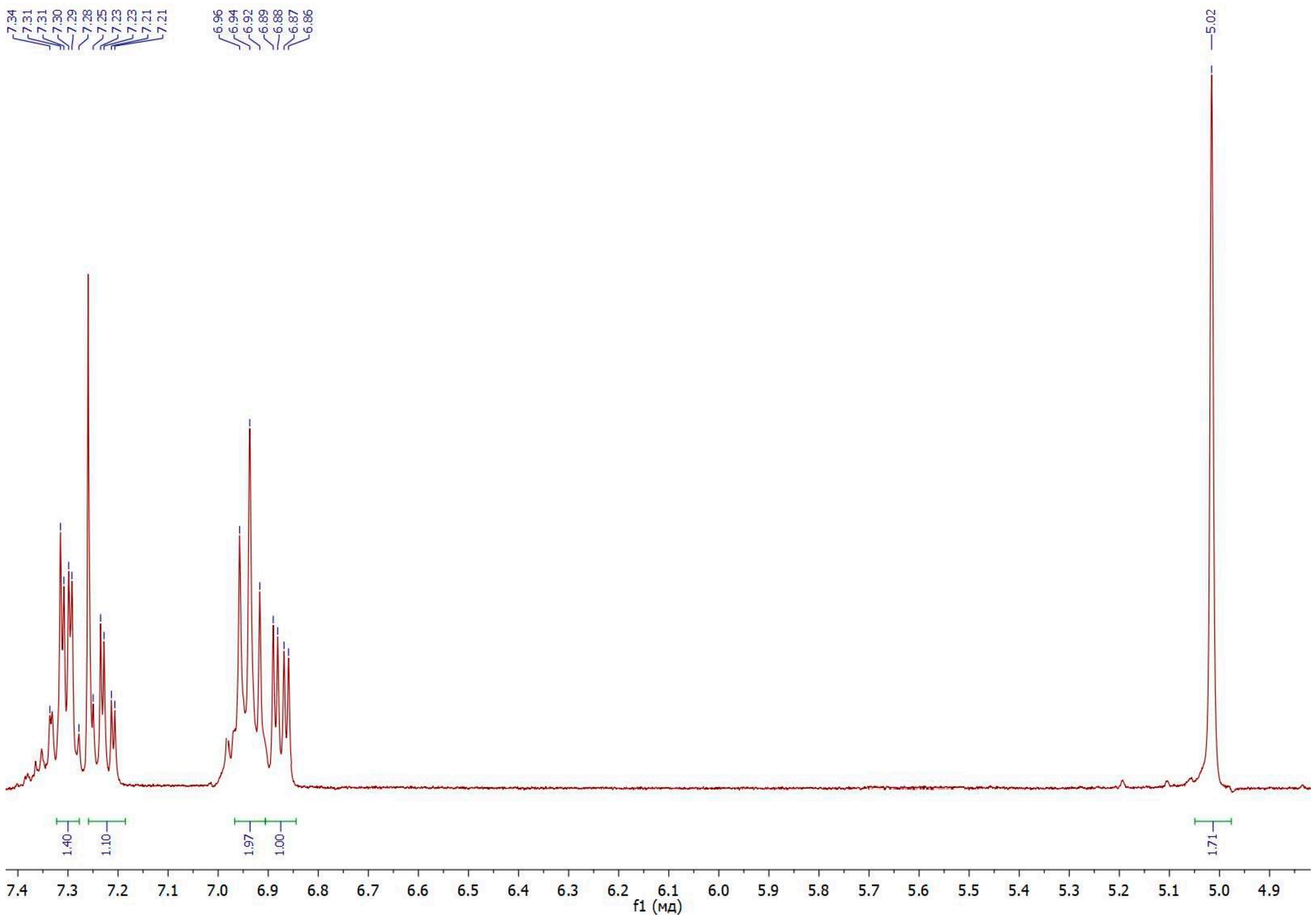

Figure S8. ^{1}H NMR spectrum of compound **3c** (500 MHz, $CDCl_3$)

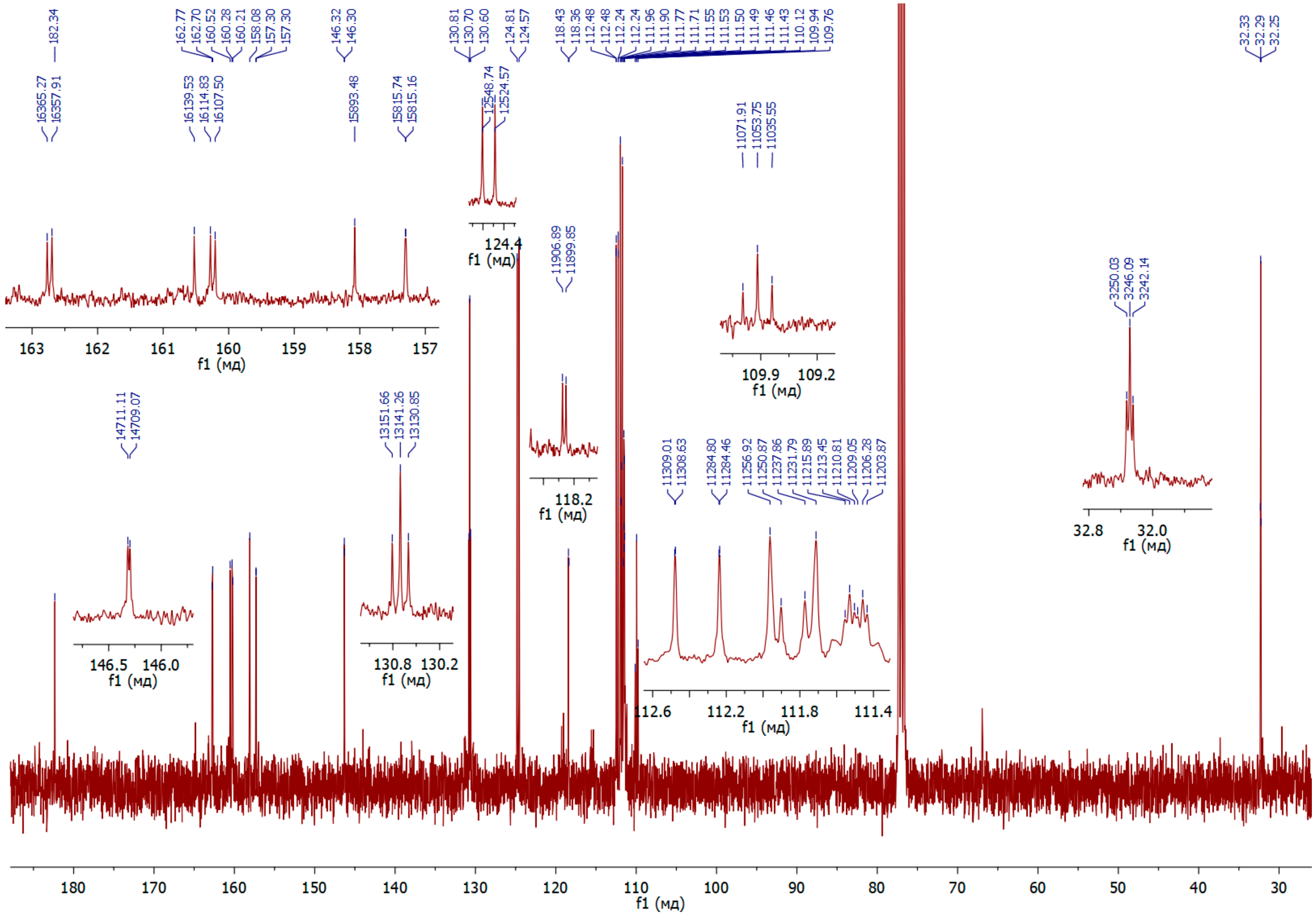

Figure S9. ^{13}C-{^{1}H} NMR spectrum of compound **3c** (126 MHz, $CDCl_3$)

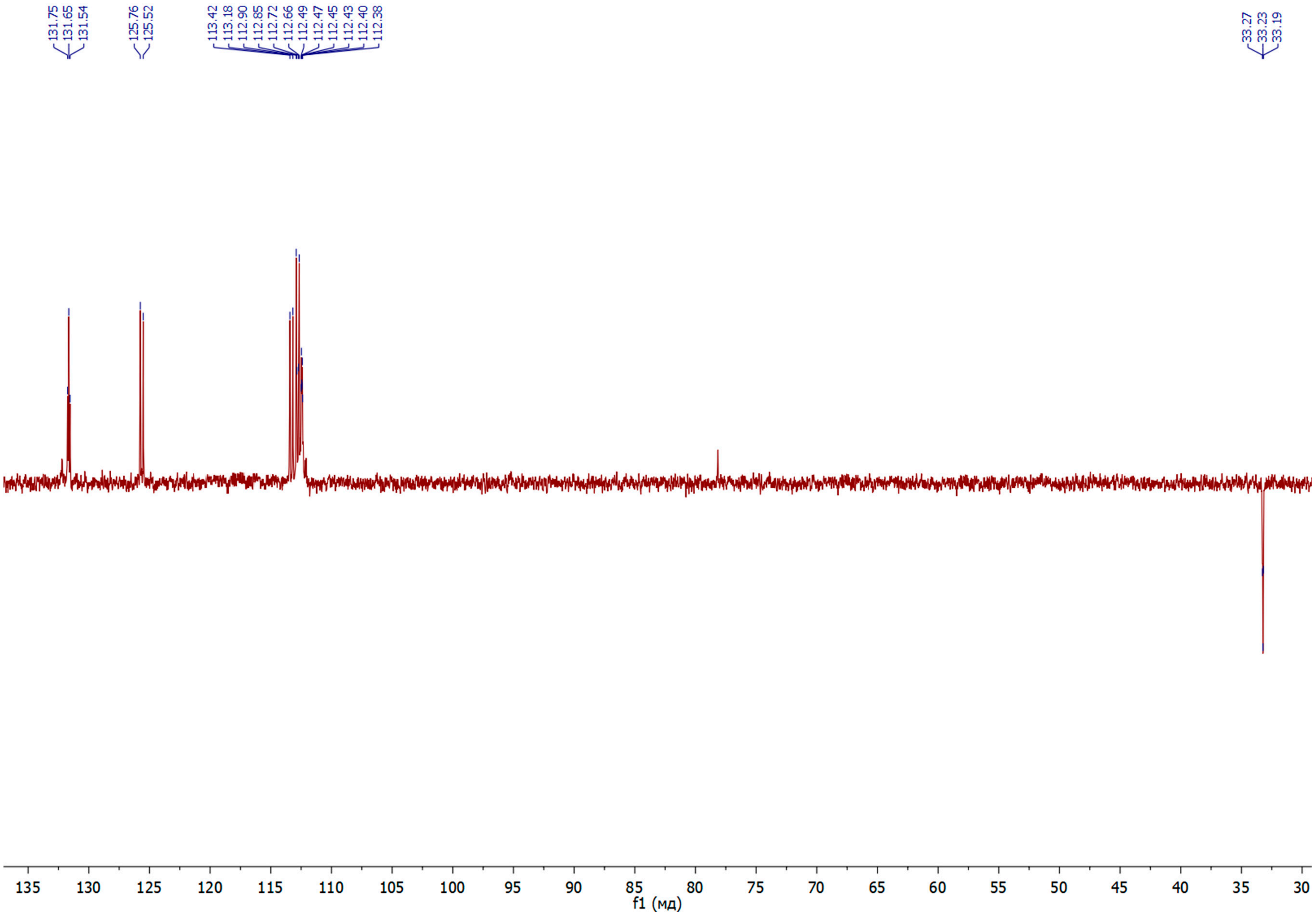

Figure S10. ^{13}C (dept) NMR spectrum of compound **3c** (126 MHz, $CDCl_3$)

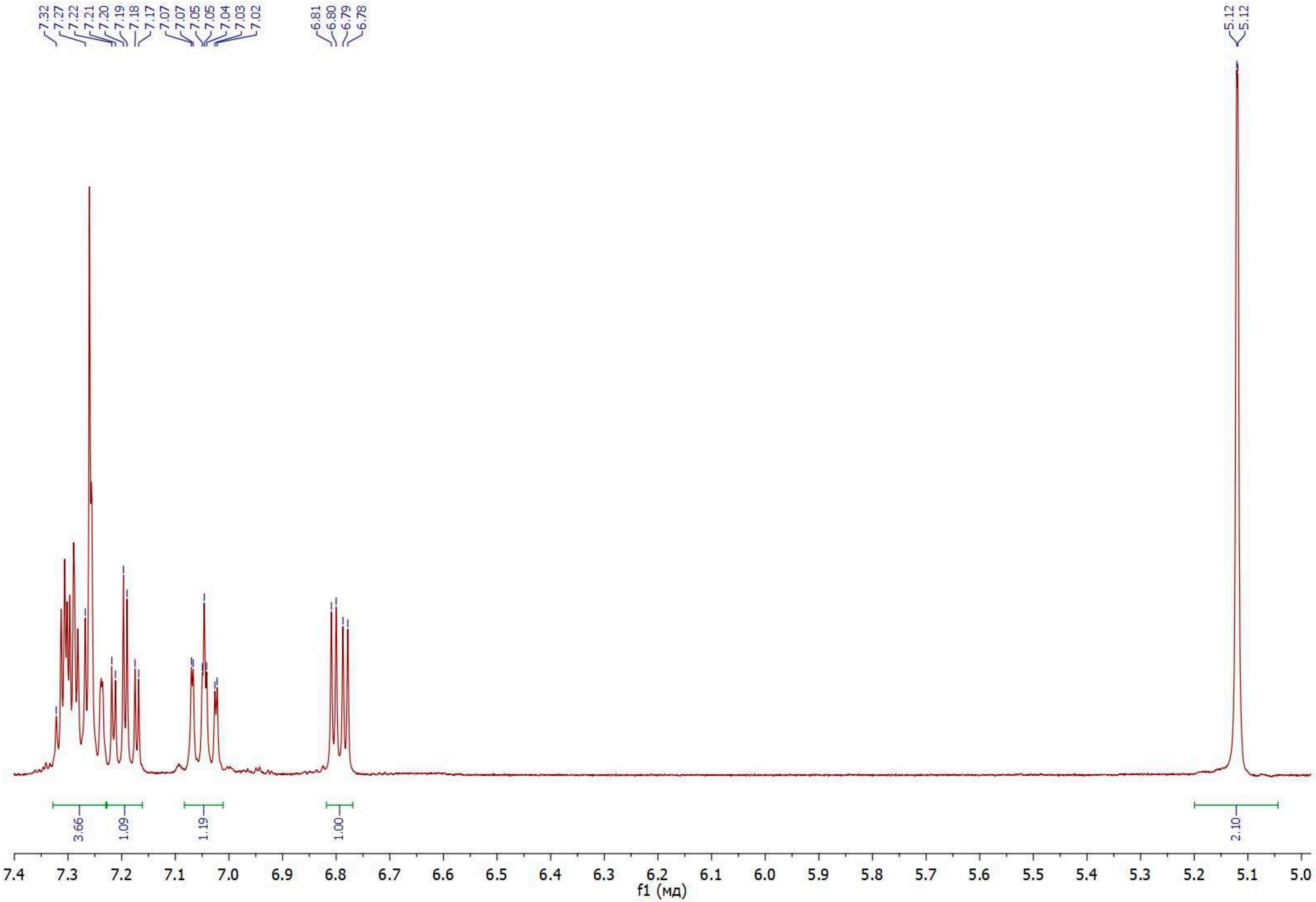

Figure S11. ^{1}H NMR spectrum of compound **3d** (500 MHz, $CDCl_3$)

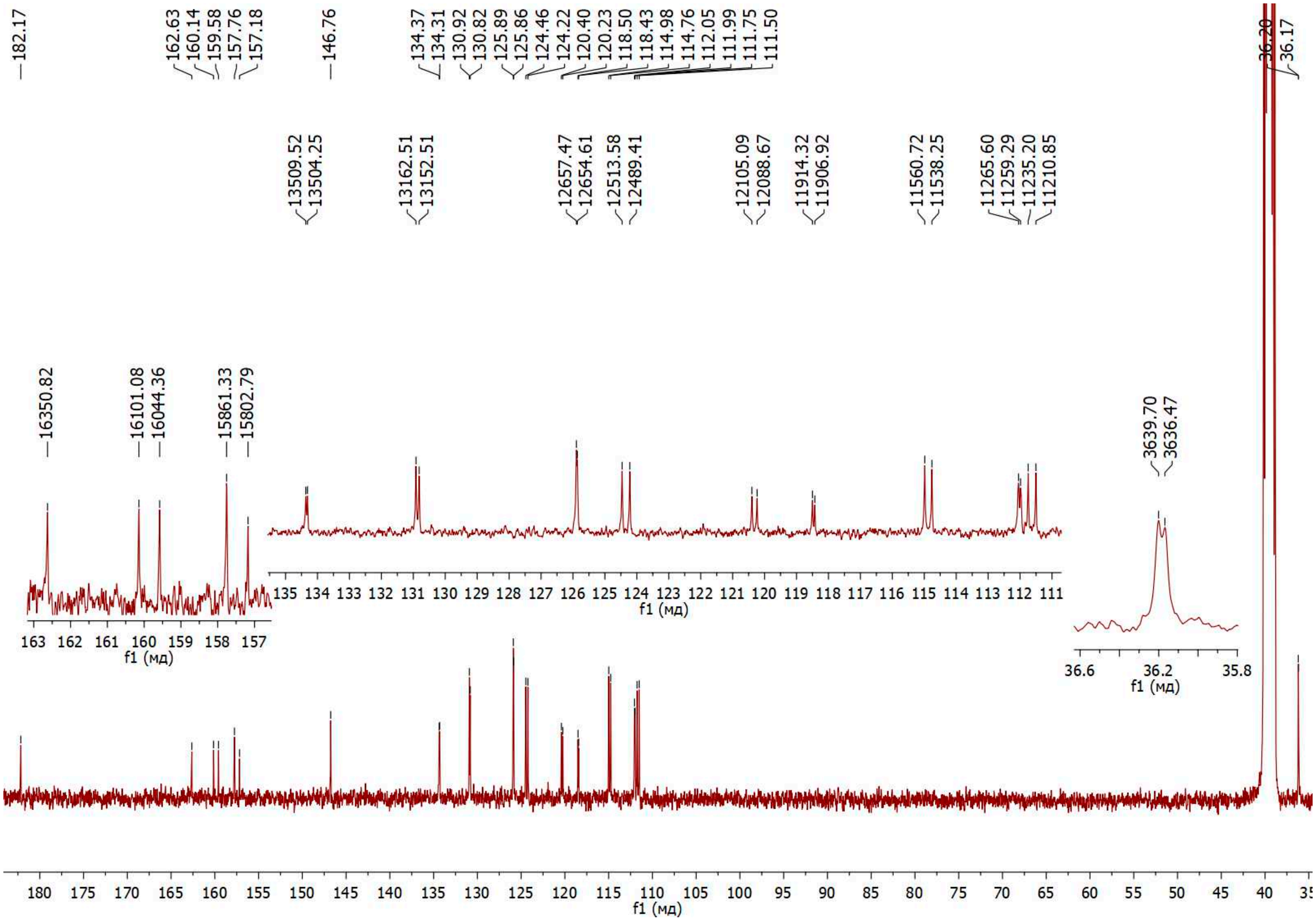

Figure S12. ^{13}C-{^{1}H} NMR spectrum of compound **3d** (126 MHz, DMSO-d_6)

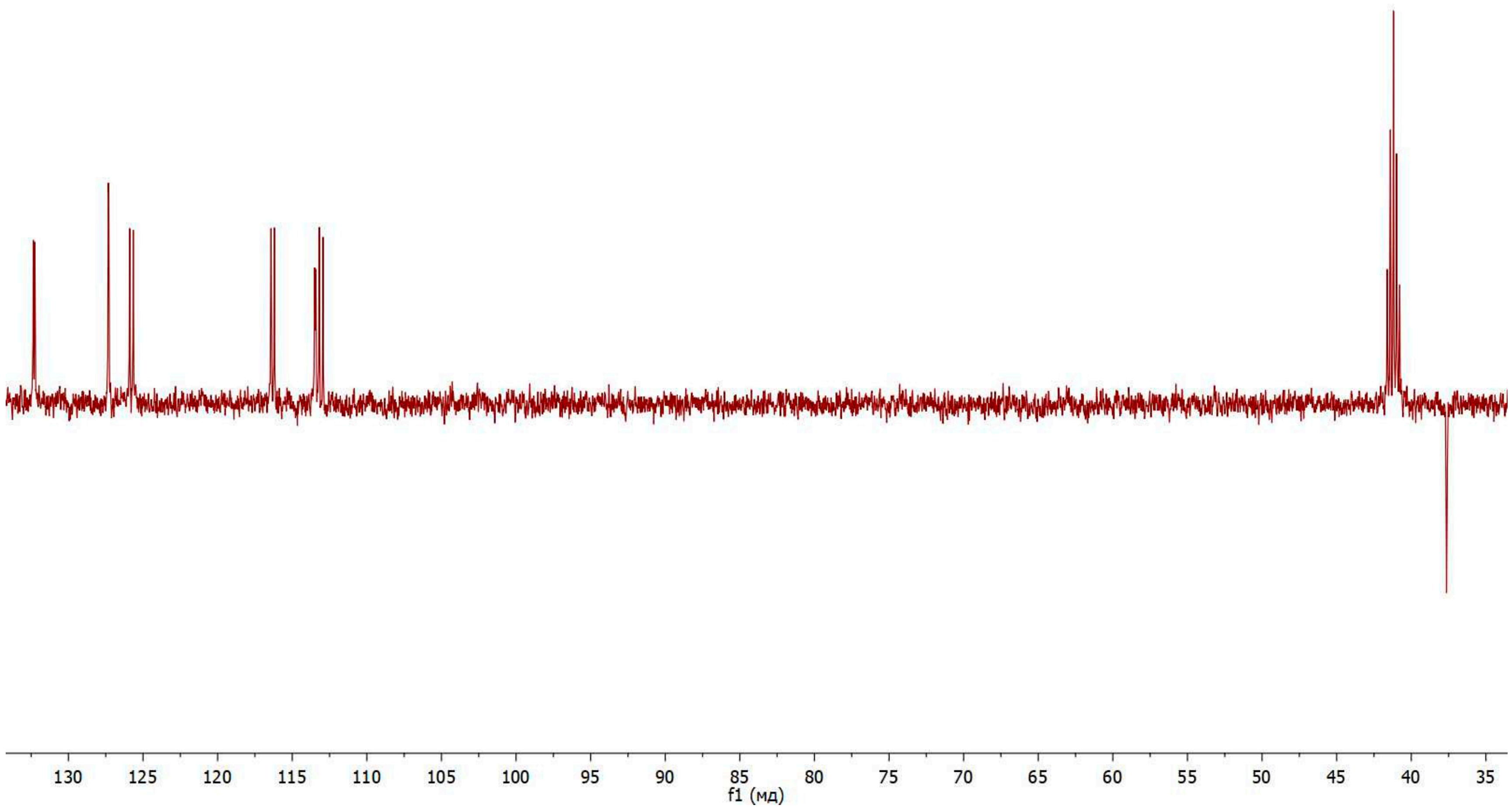

Figure S13. ^{13}C (dept) NMR spectrum of compound **3d** (126 MHz, DMSO-d_6)

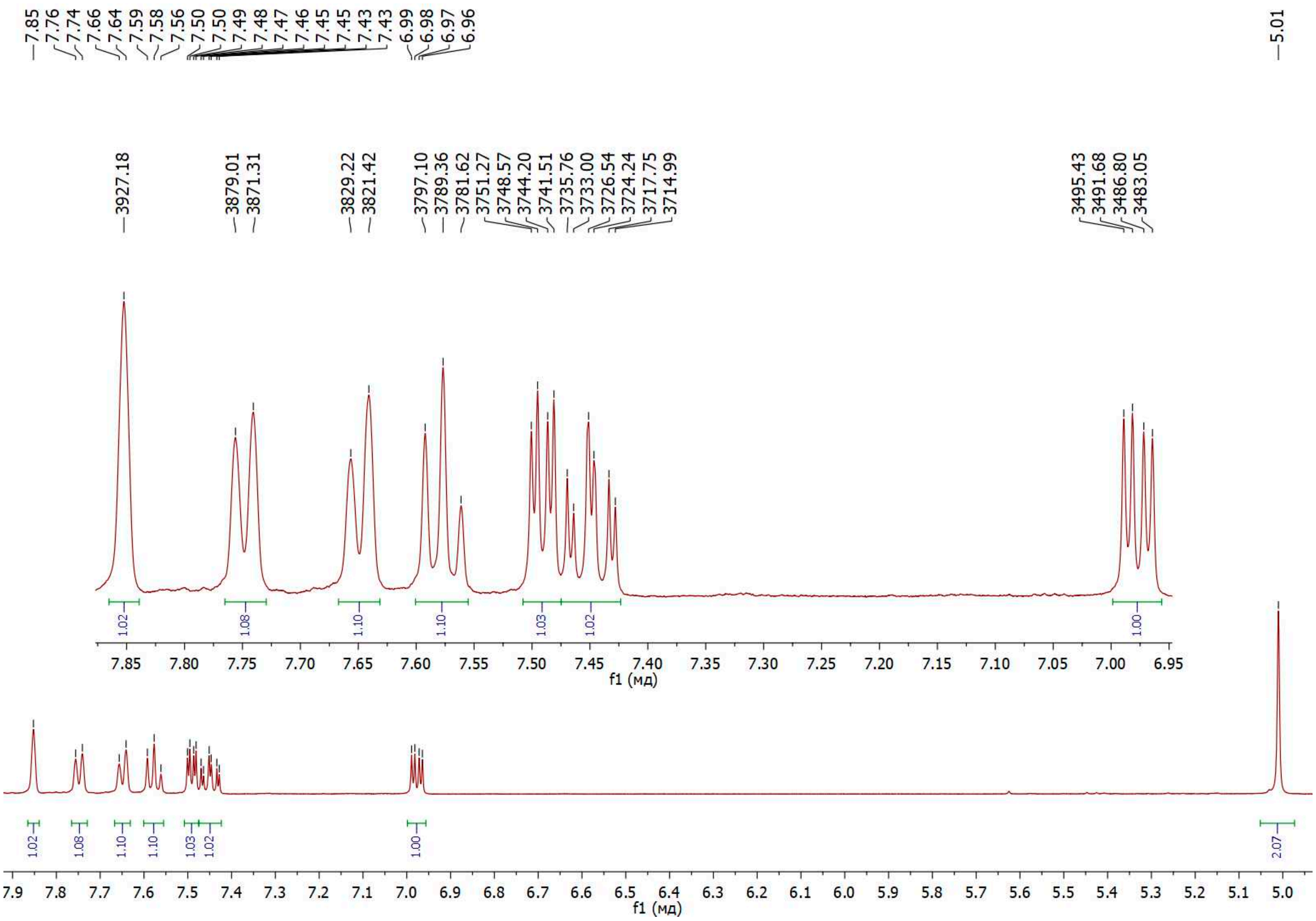

Figure S14. ^{1}H NMR spectrum of compound **3e** (400 MHz, DMSO-d_6)

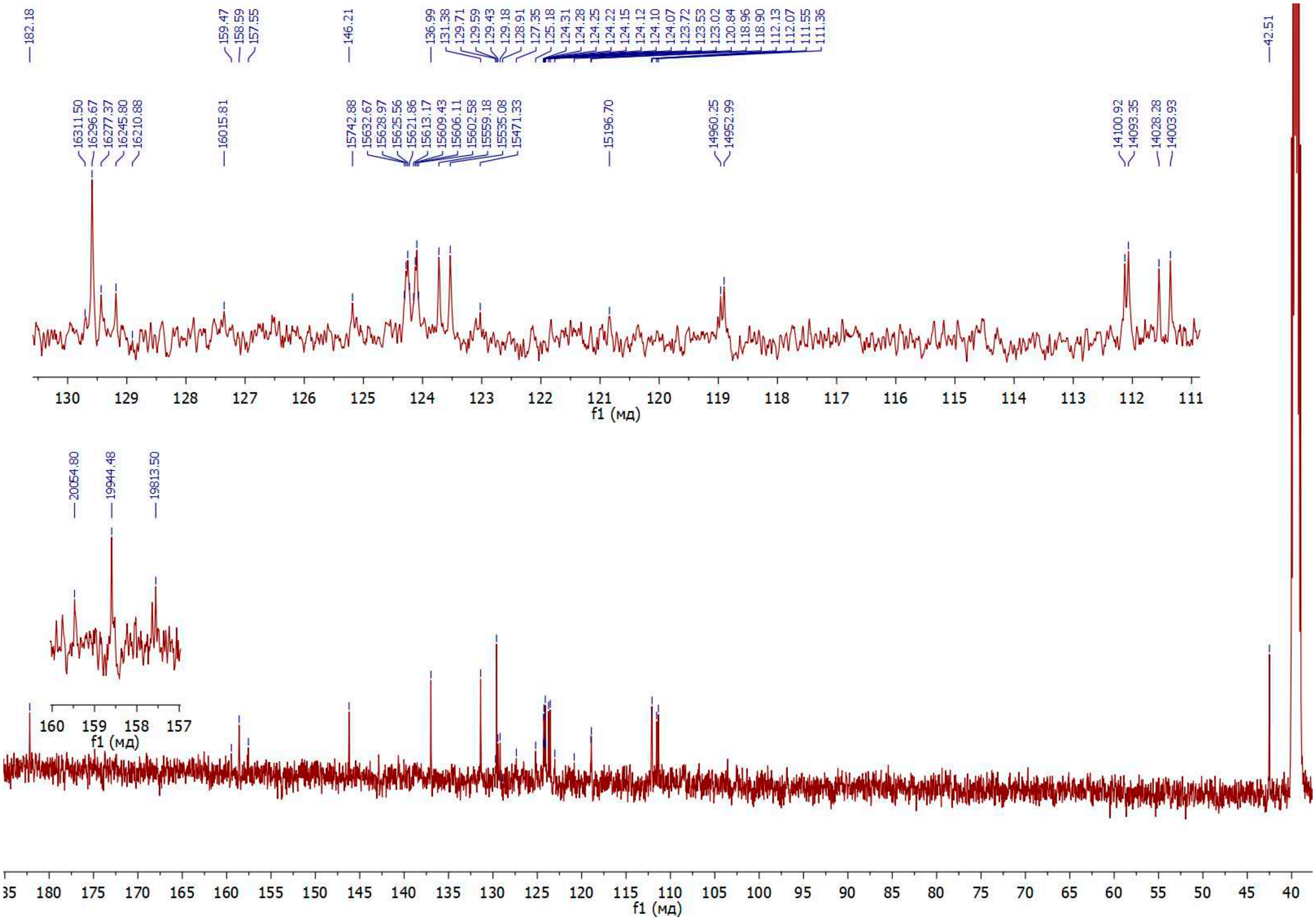

Figure S15. ^{13}C-{^{1}H} NMR spectrum of compound **3e** (101 MHz, DMSO-d_6)

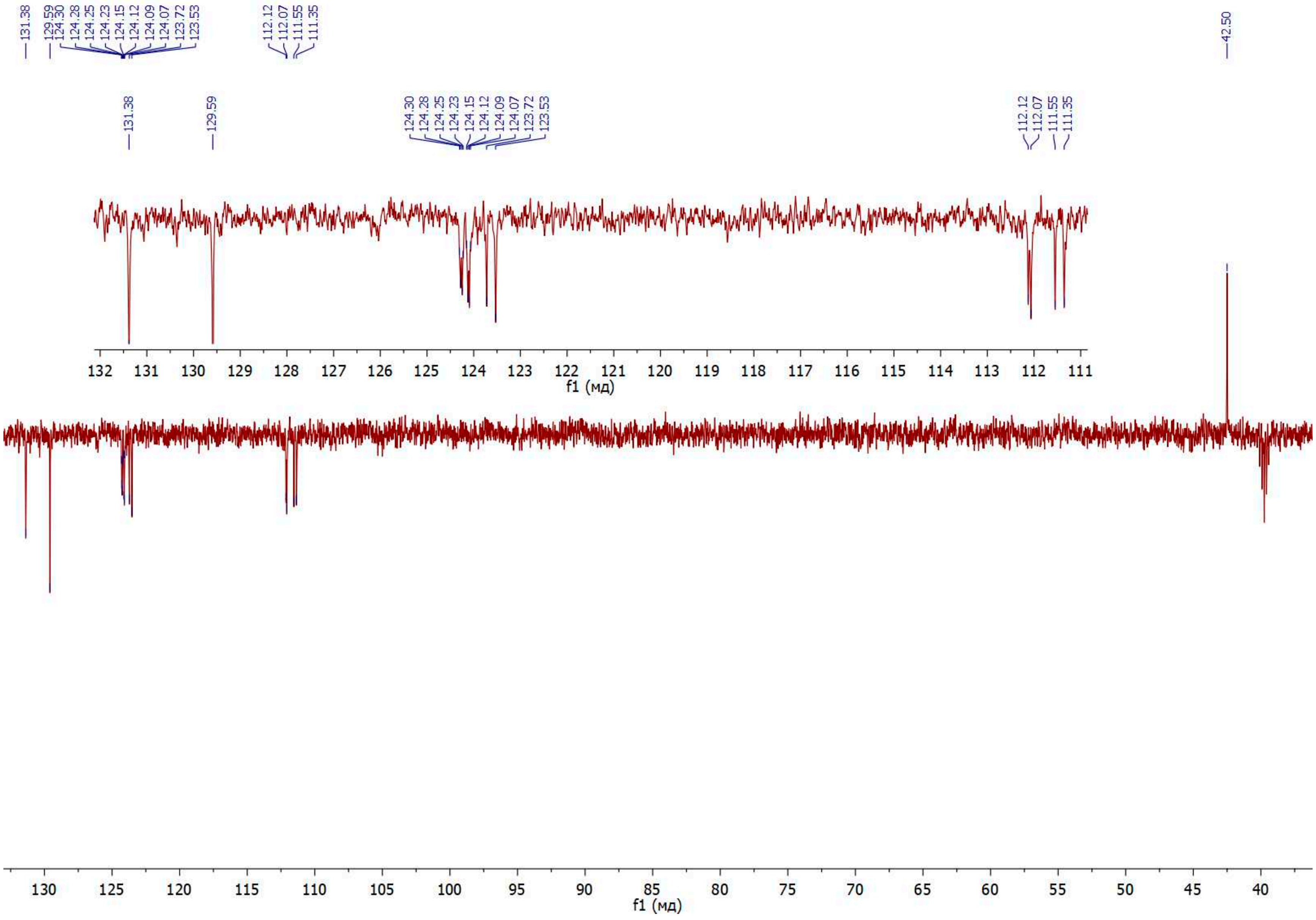

Figure S16. ^{13}C (dept) NMR spectrum of compound **3e** (101 MHz, DMSO-d_6)

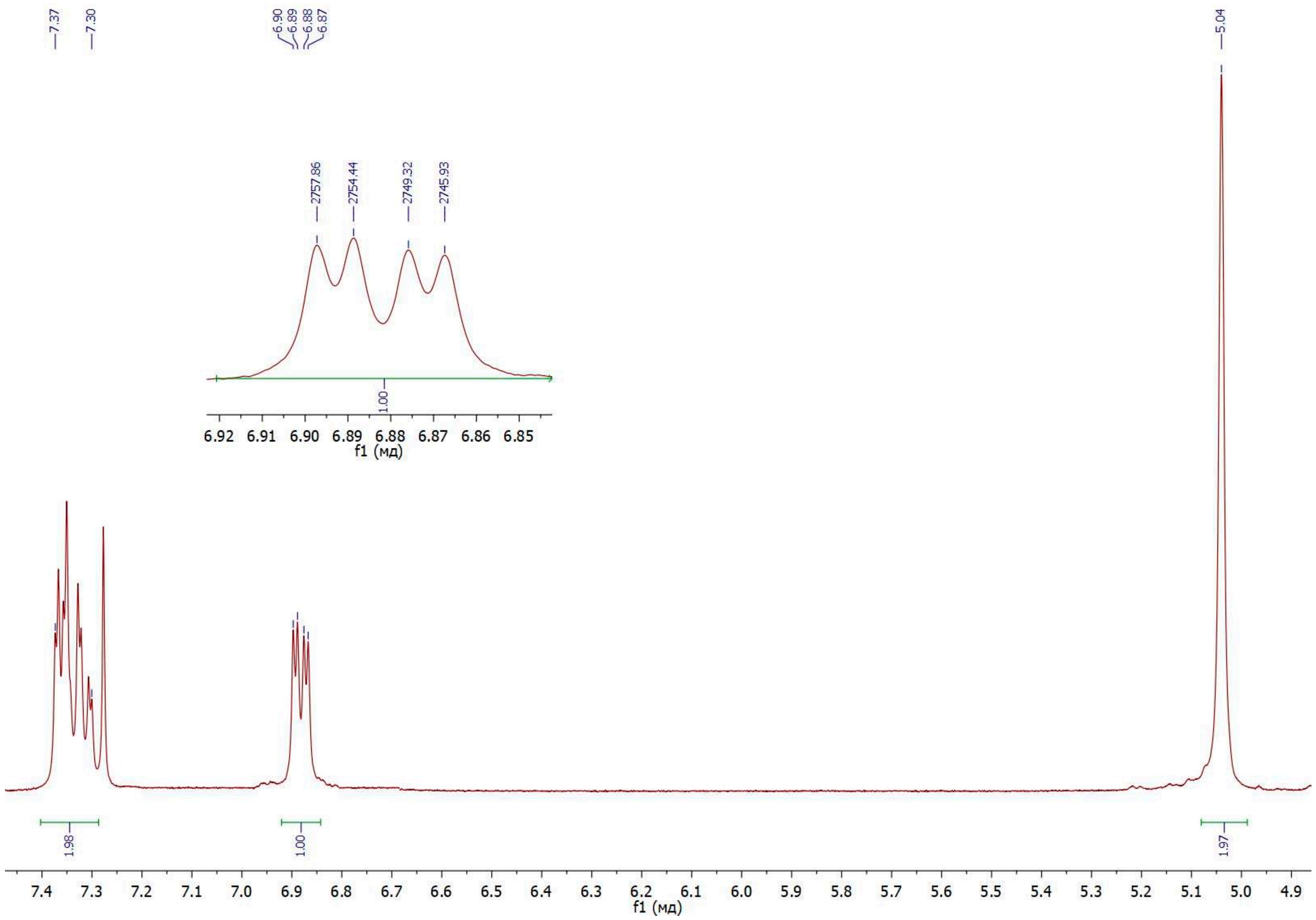

Figure S17. ^{1}H NMR spectrum of compound **3f** (500 MHz, $CDCl_3$)

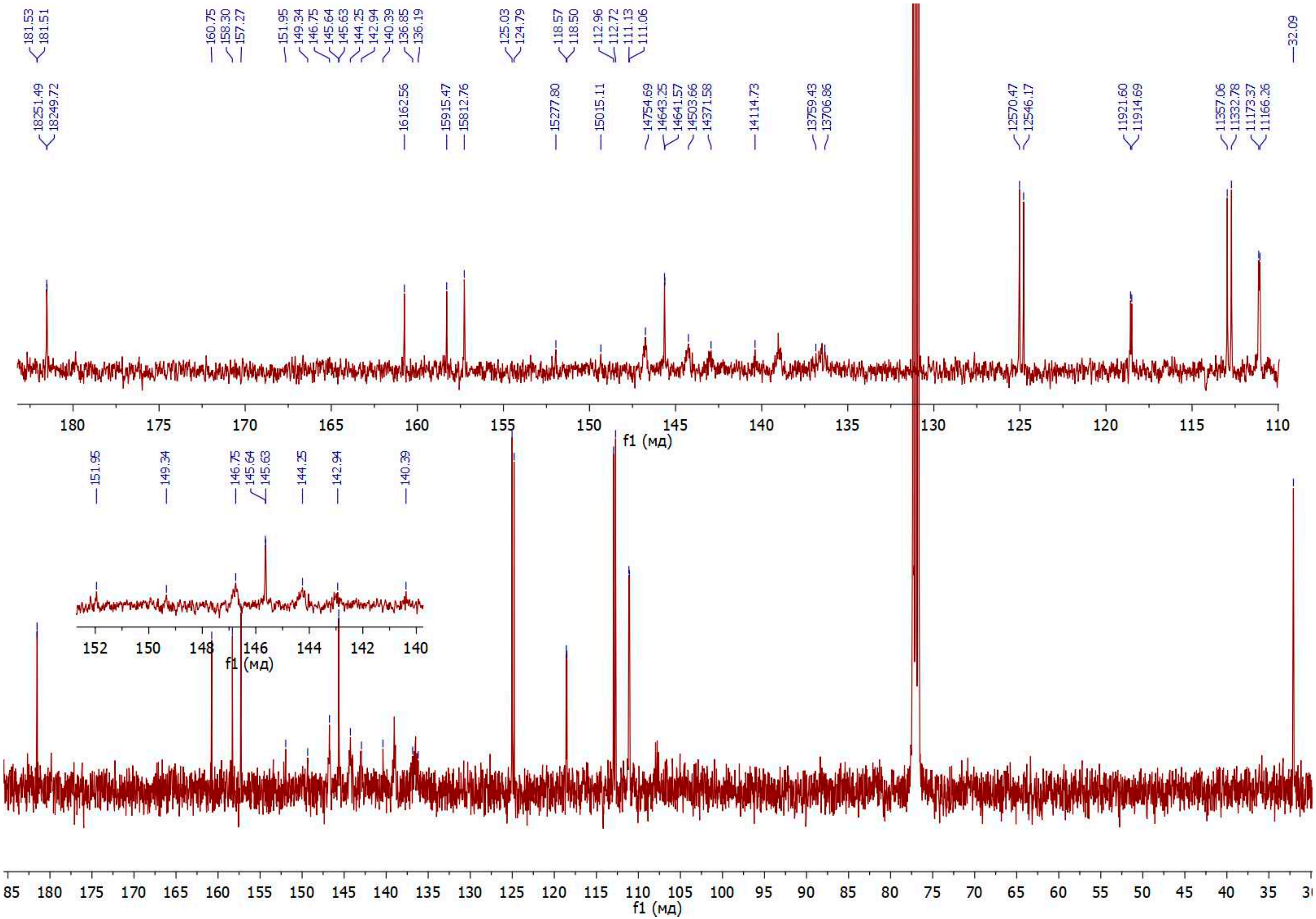

Figure S18. ^{13}C-{^{1}H} NMR spectrum of compound **3f** (126 MHz, $CDCl_3$)

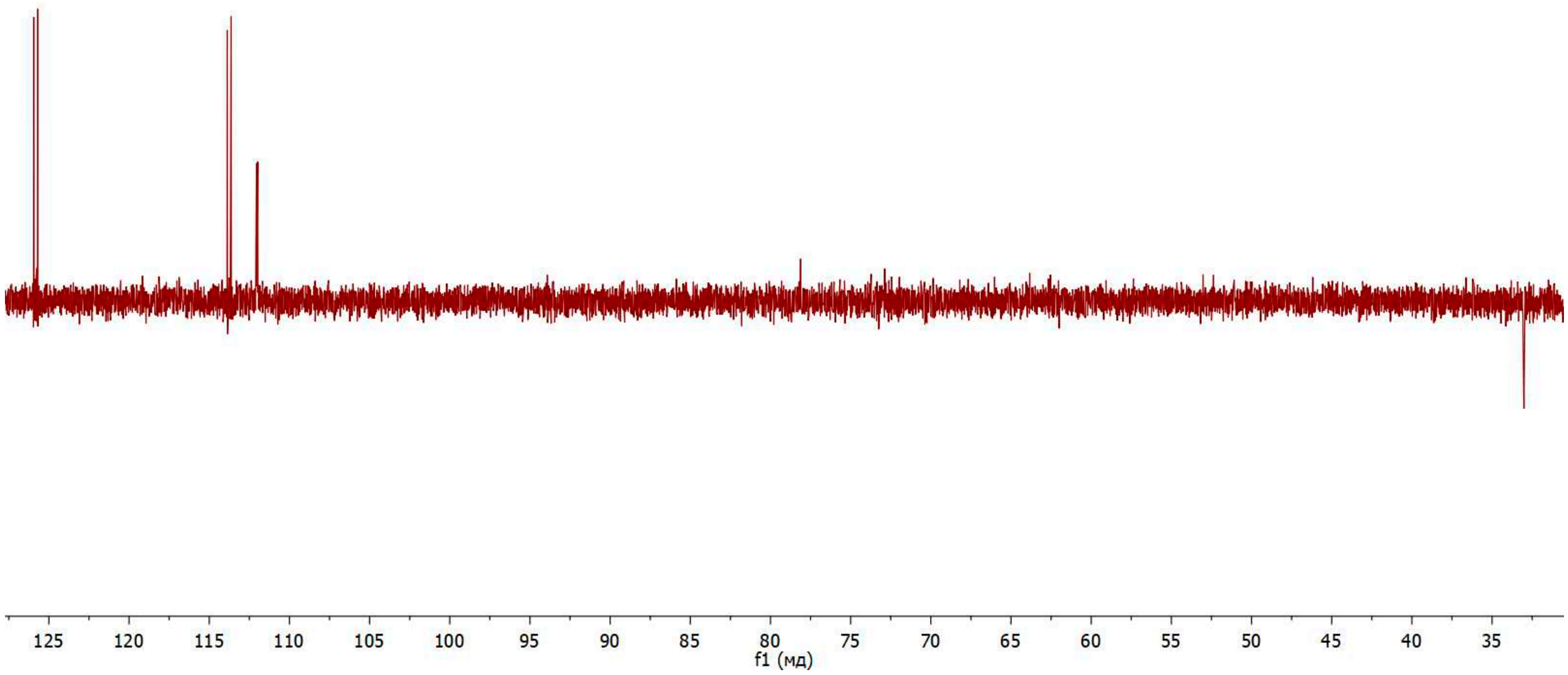

Figure S19. ^{13}C (dept) NMR spectrum of compound **3f** (126 MHz, $CDCl_3$)

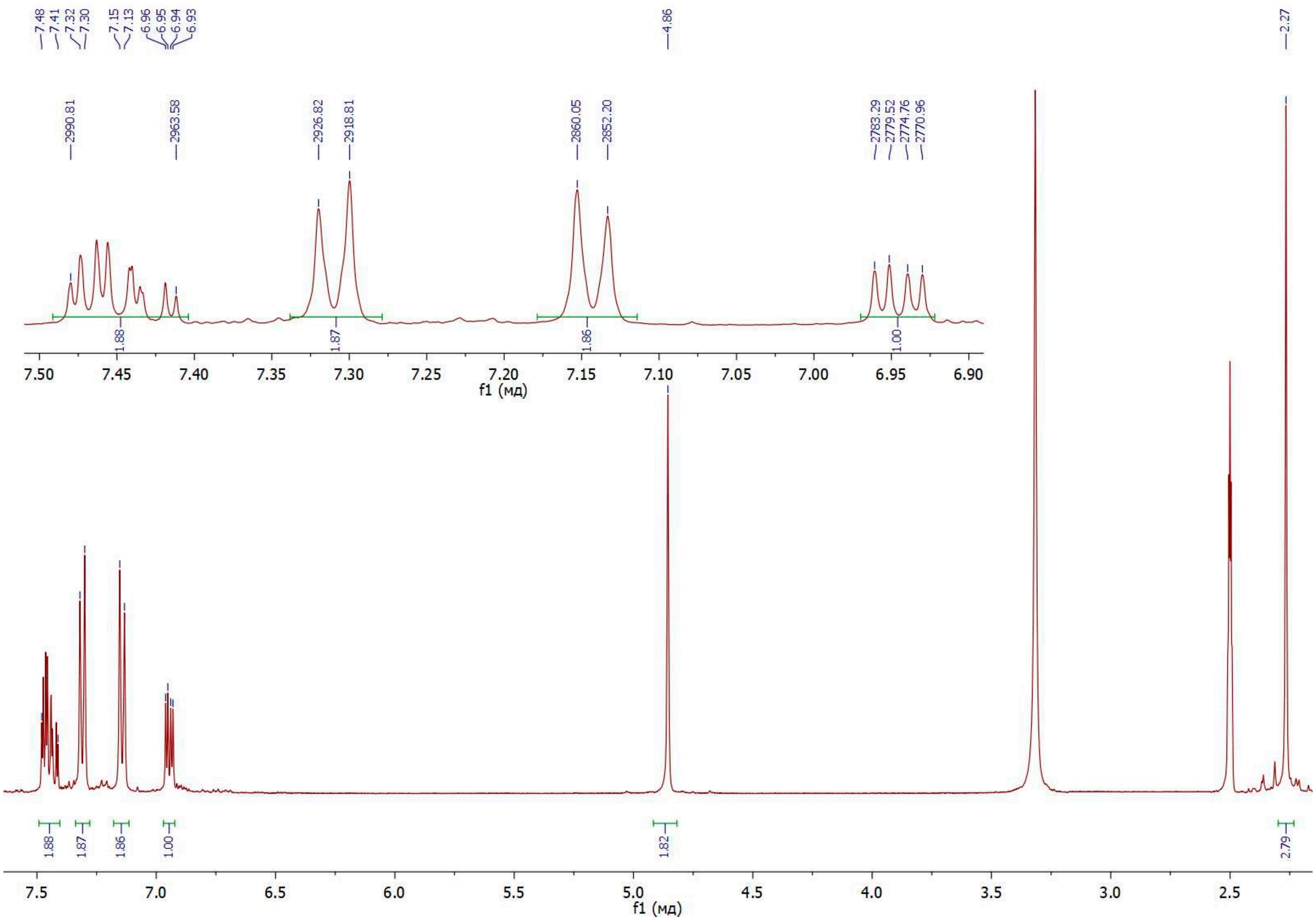

Figure S20. ^{1}H NMR spectrum of compound **4a** (500 MHz, DMSO-d_6)

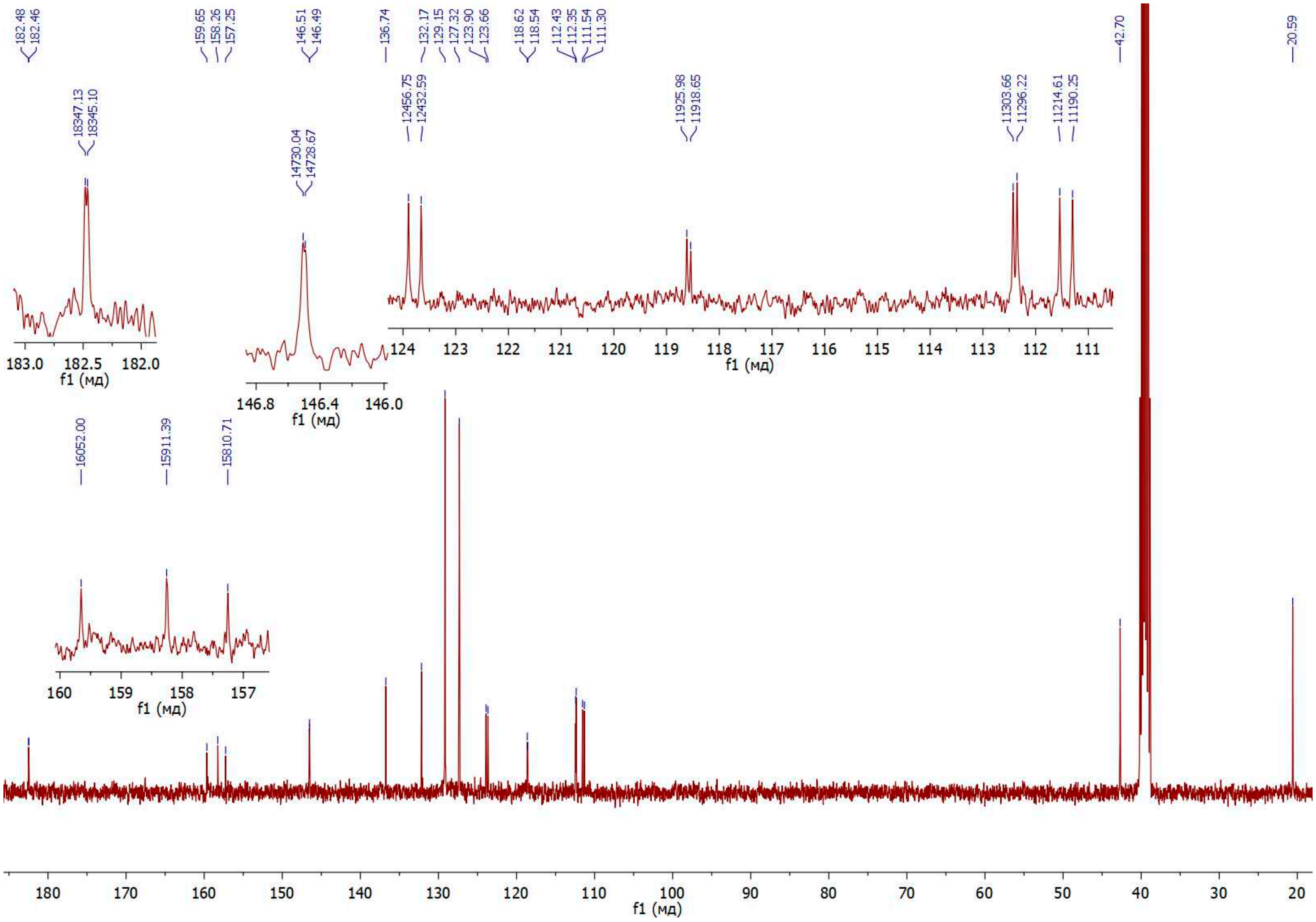

Figure S21. ^{13}C-{^{1}H} NMR spectrum of compound **4a** (126 MHz, DMSO-d_6)

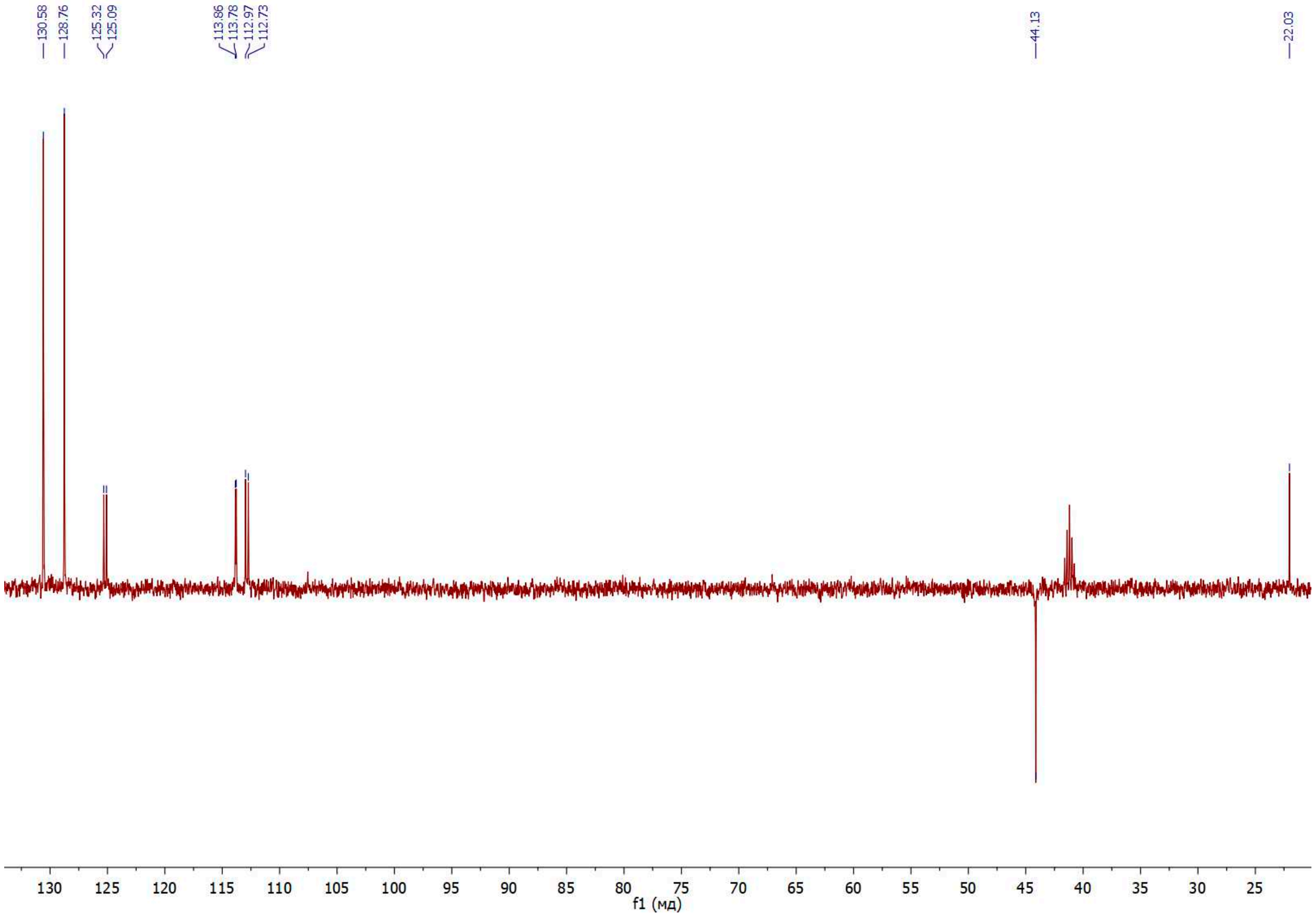

Figure S22. ^{13}C (dept) NMR spectrum of compound **4a** (101 MHz, DMSO-d_6)

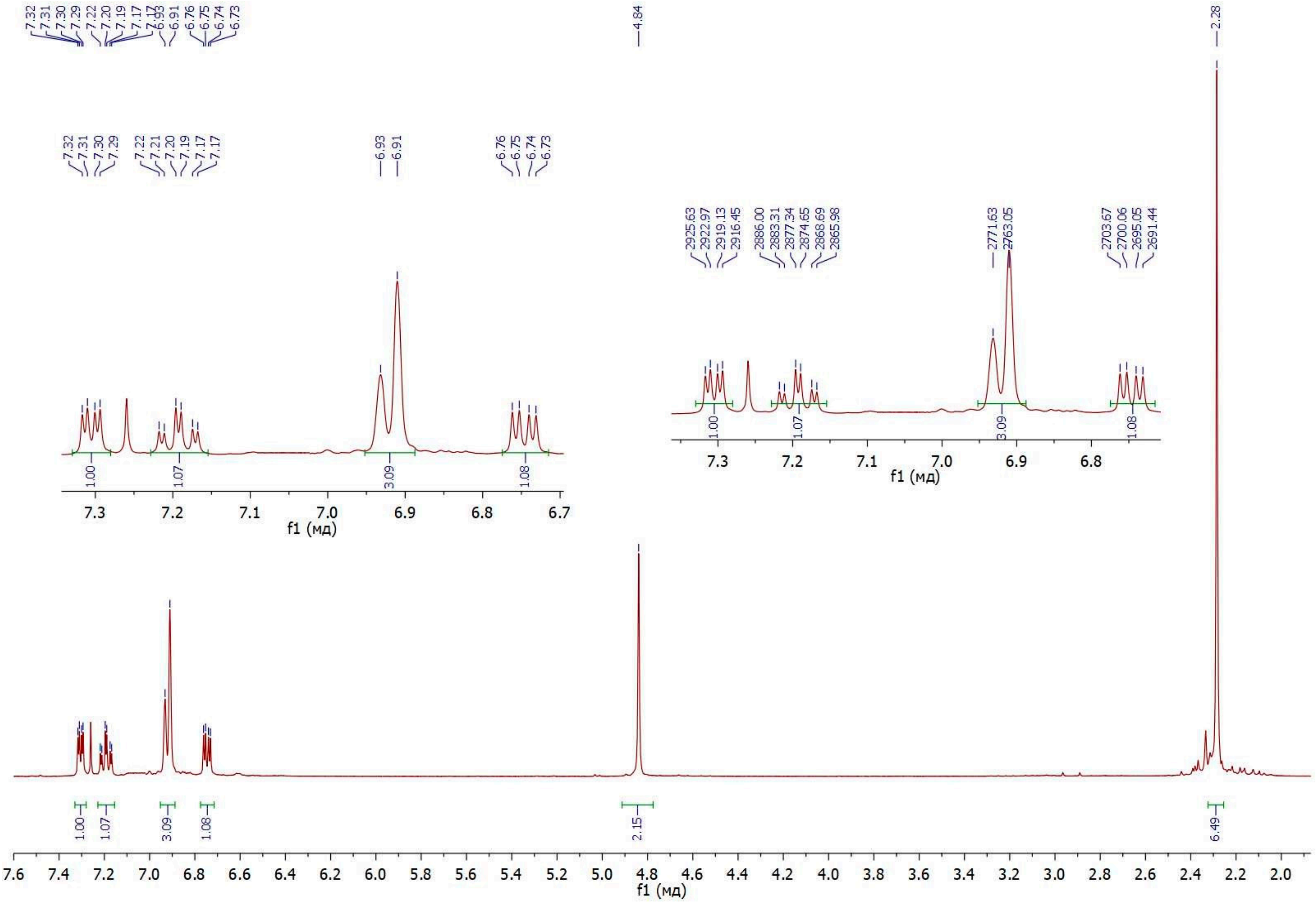

Figure S23. ^{1}H NMR spectrum of compound **4b** (400 MHz, $CDCl_3$)

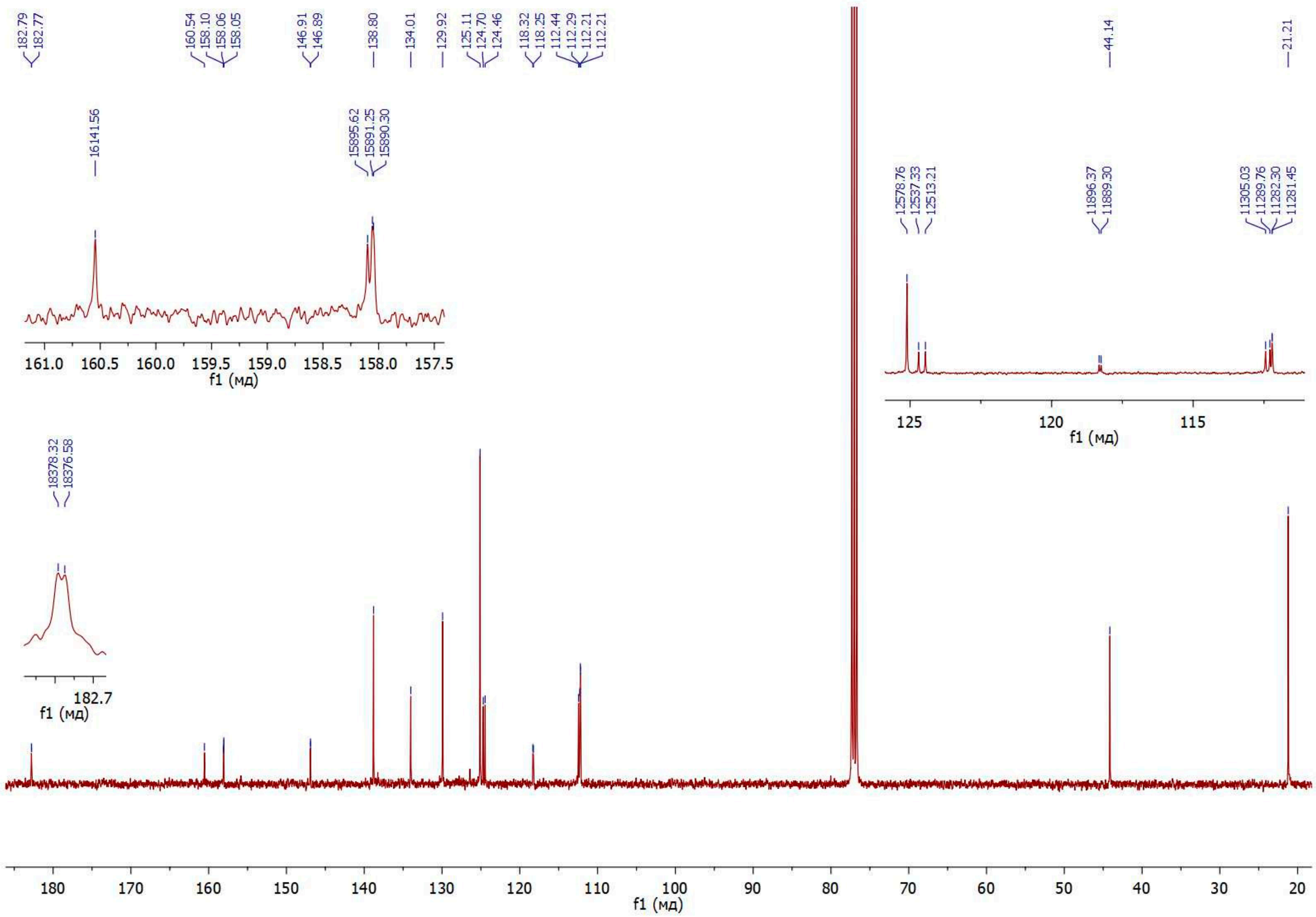

Figure S24. ^{13}C-{^{1}H} NMR spectrum of compound **4b** (101 MHz, $CDCl_3$)

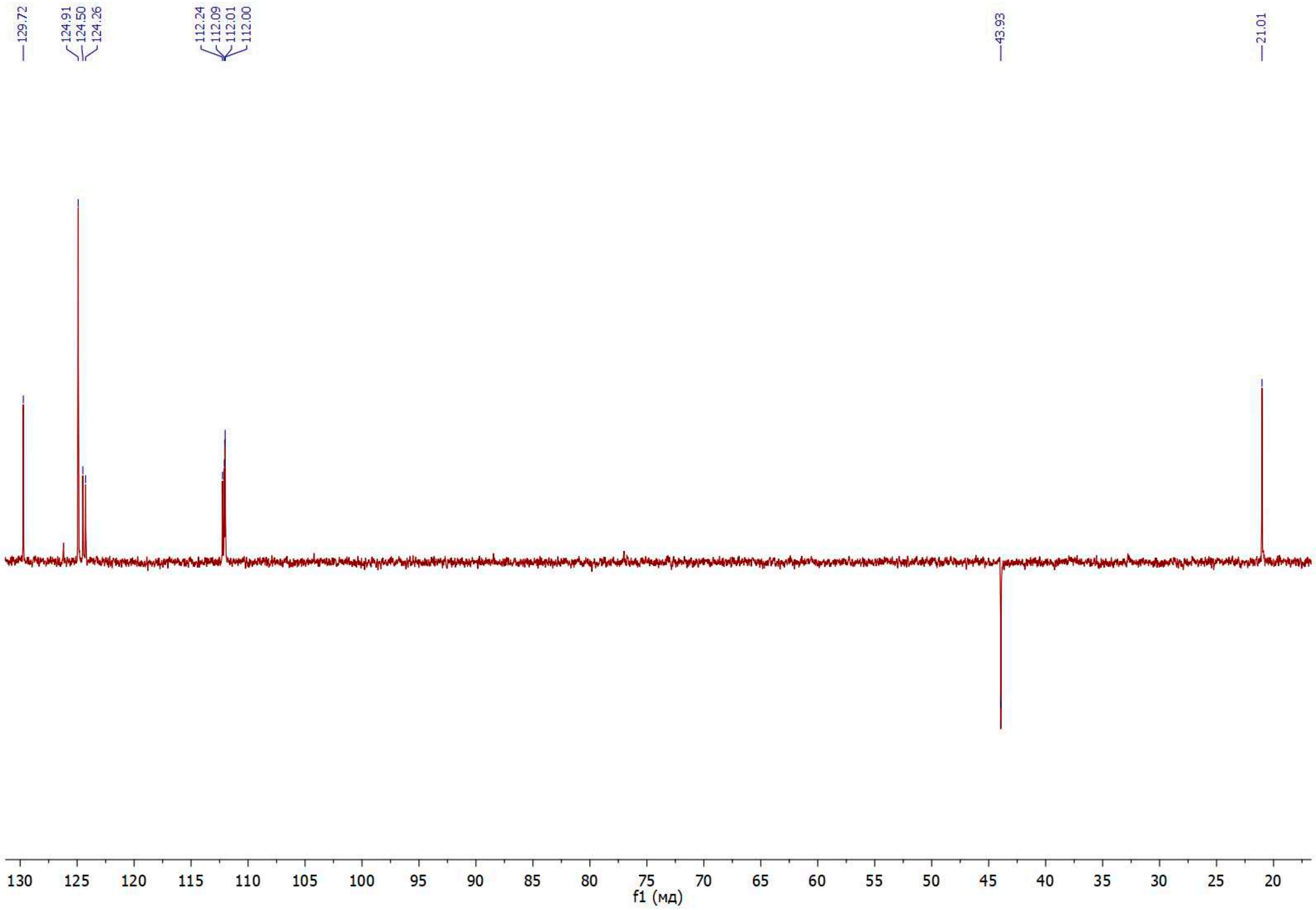

Figure S25. ^{13}C (dept) NMR spectrum of compound **4b** (101 MHz, $CDCl_3$)

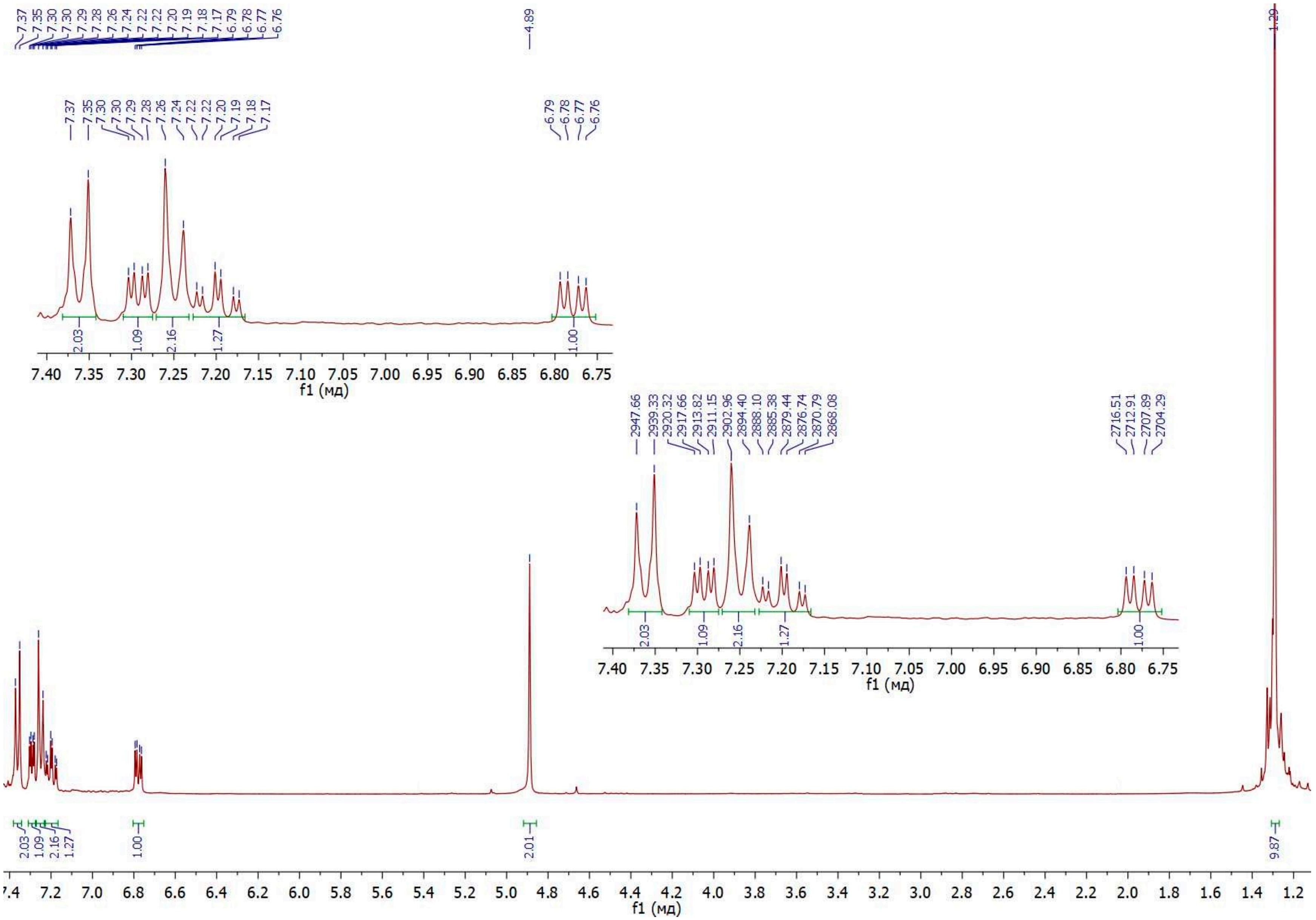

Figure S26. ^{1}H NMR spectrum of compound **4c** (400 MHz, $CDCl_3$)

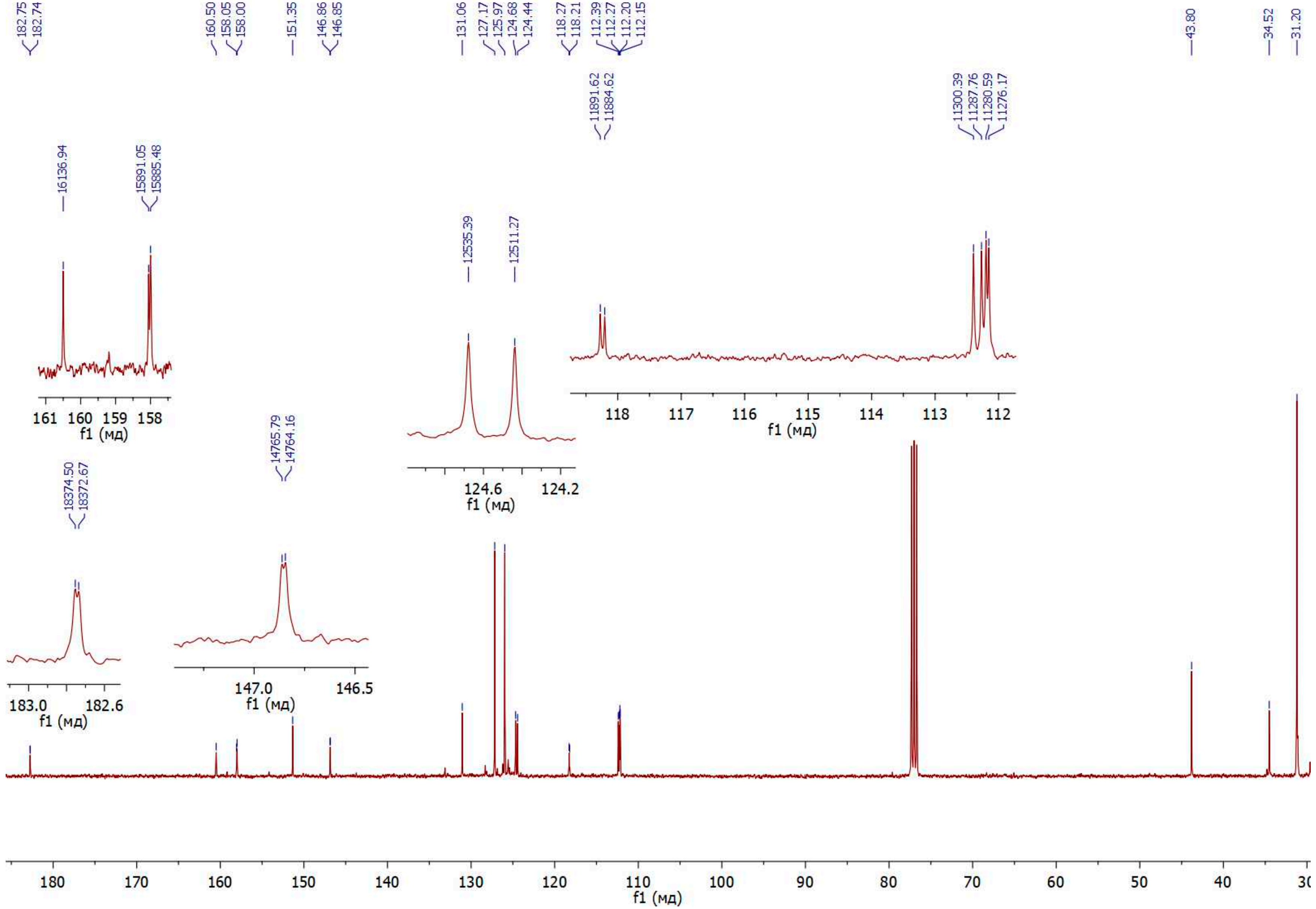

Figure S27. ^{13}C NMR spectrum of compound **4c** (101 MHz, $CDCl_3$)

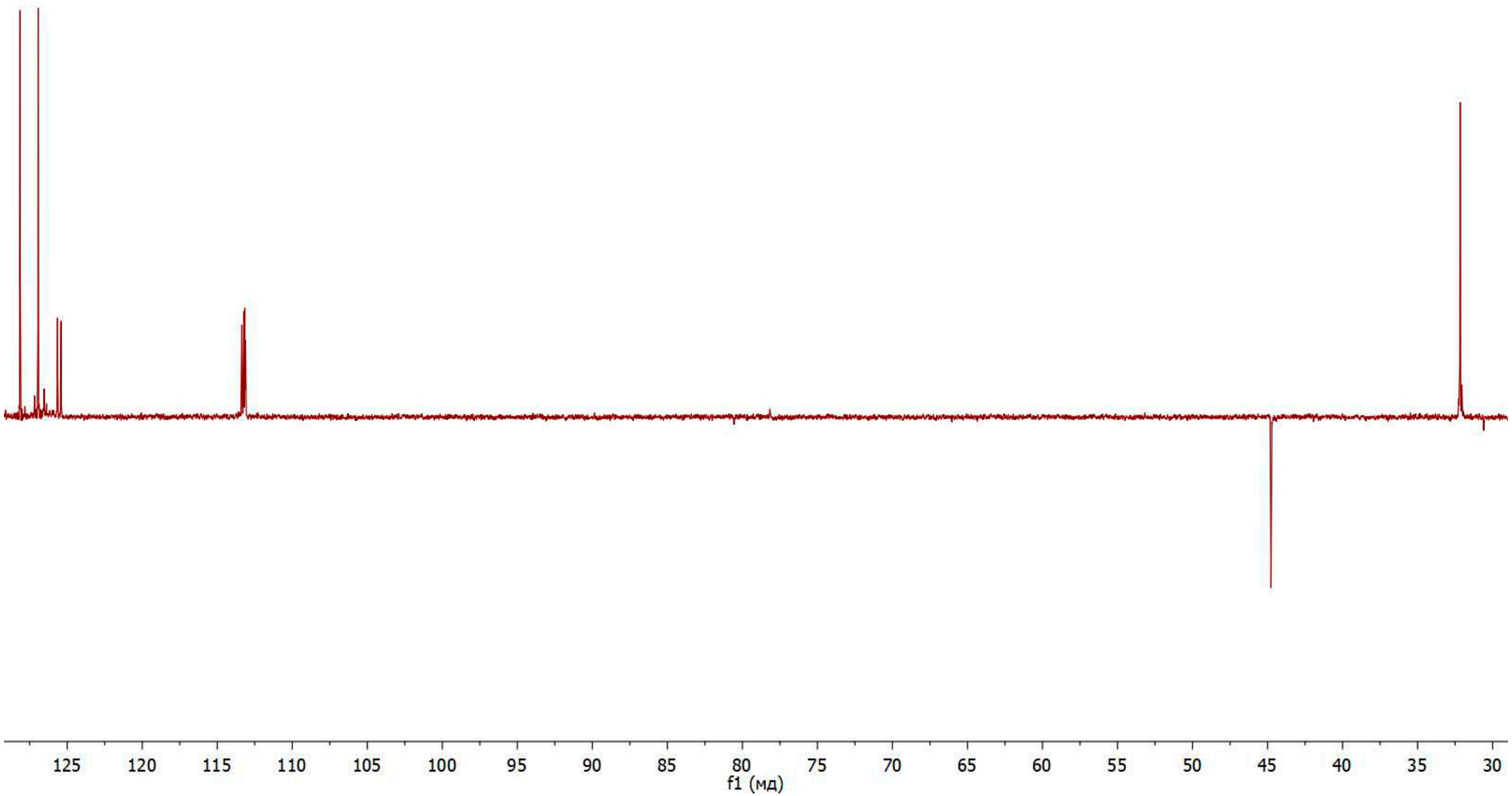

Figure S28. ^{13}C (dept) NMR spectrum of compound **4c** (101 MHz, $CDCl_3$)

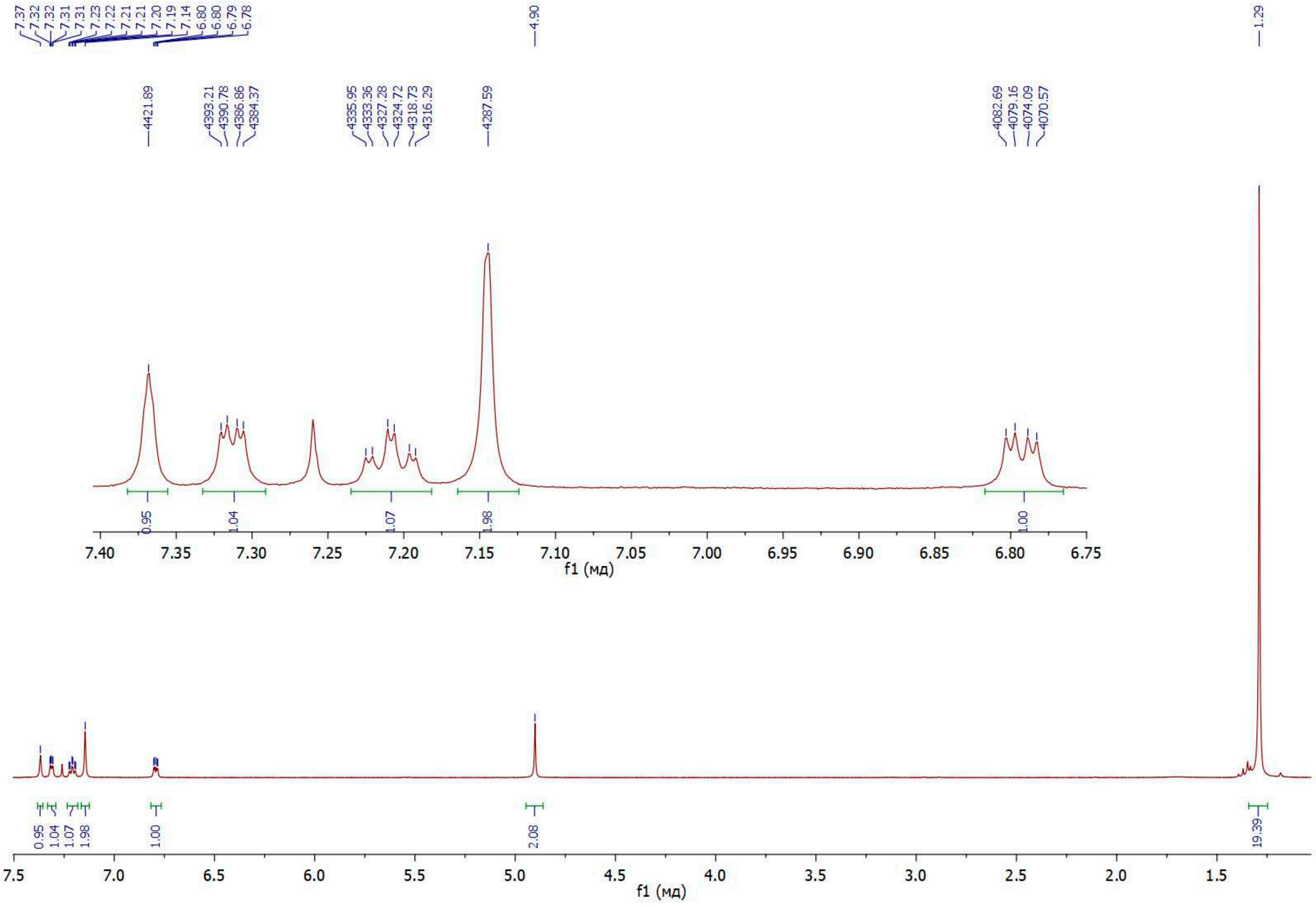

Figure S29. ^{1}H NMR spectrum of compound **4d** (400 MHz, $CDCl_3$)

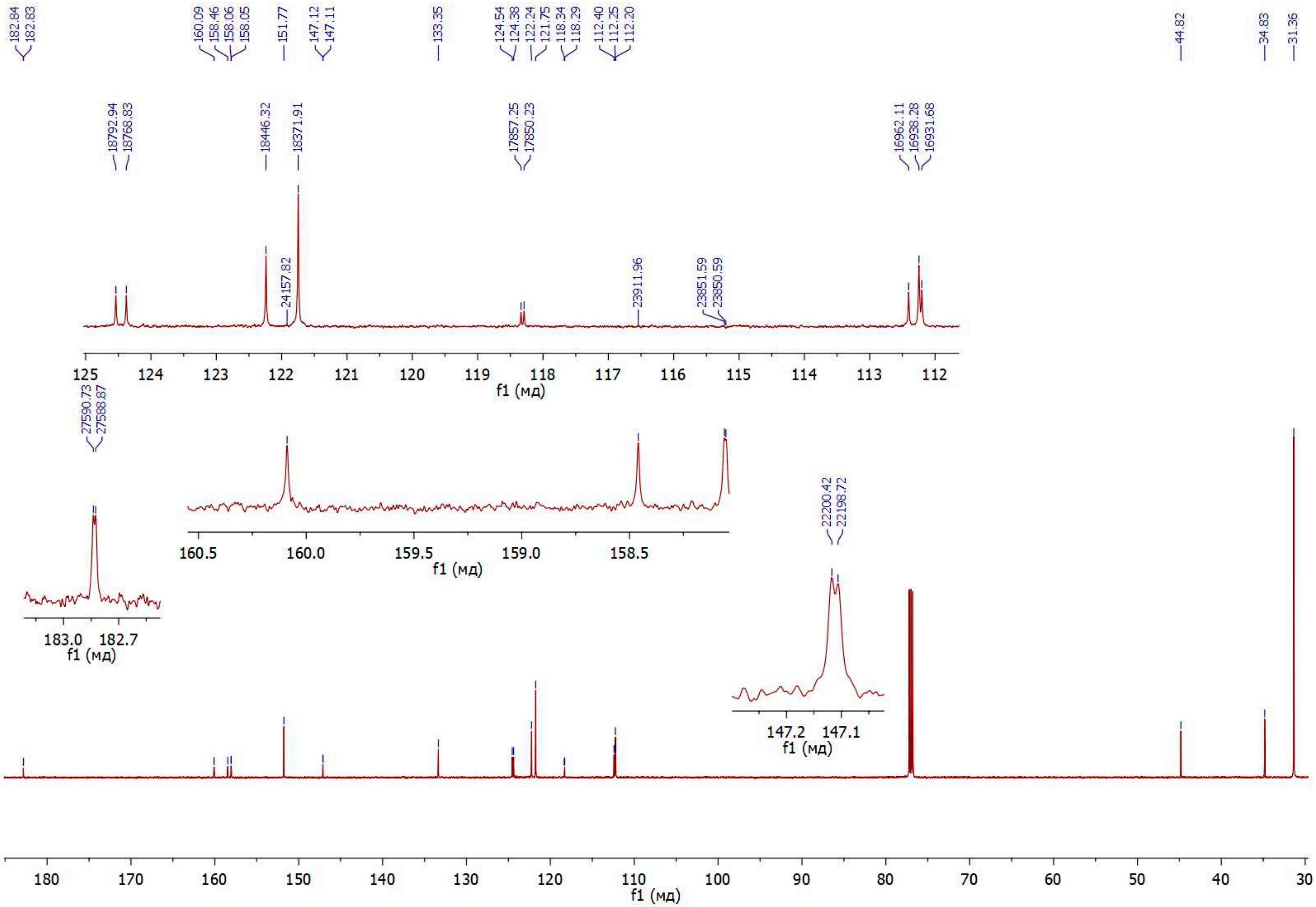

Figure S30. ^{13}C NMR spectrum of compound **4d** (101 MHz, $CDCl_3$)

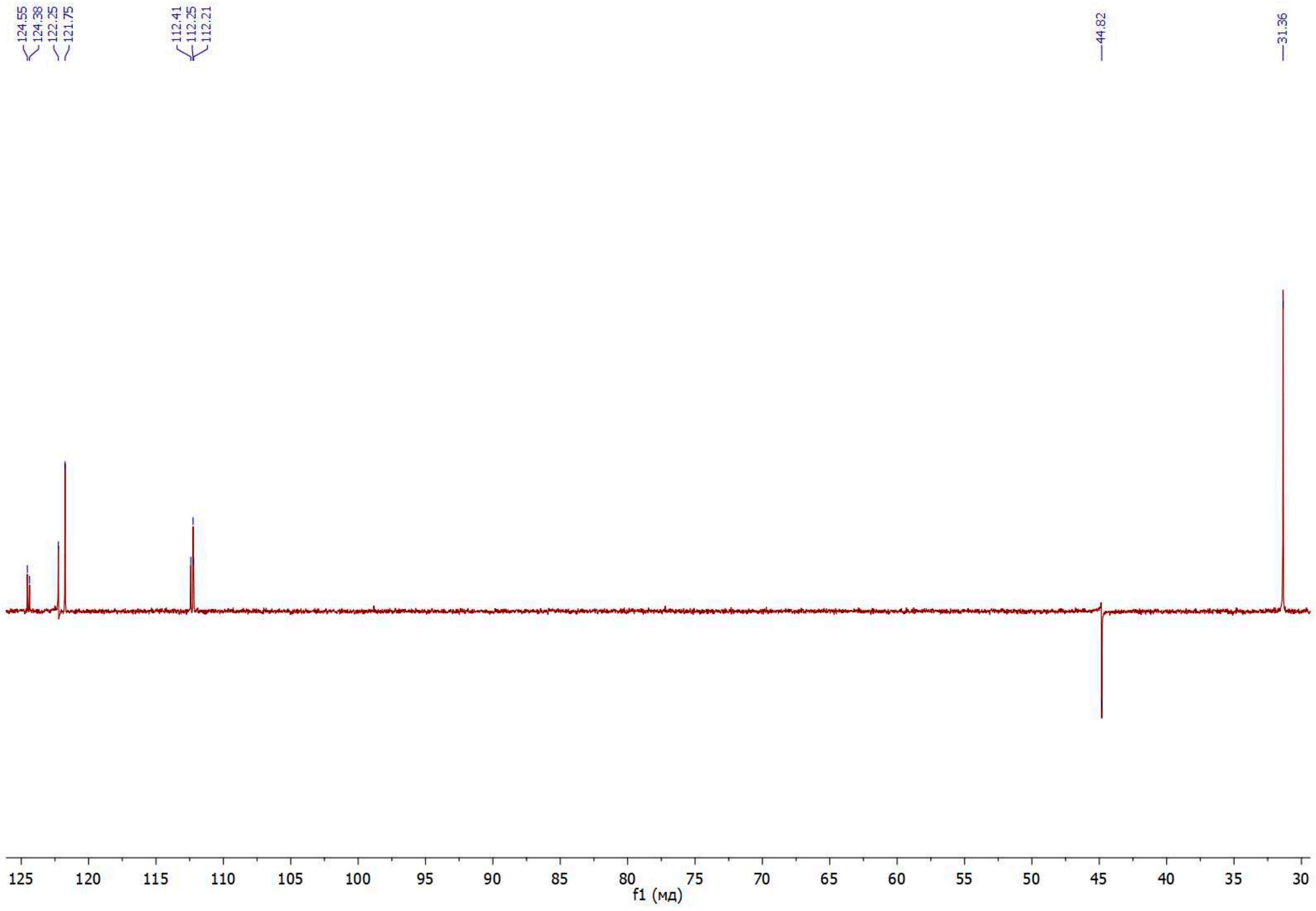

Figure S31. ^{13}C (dept) NMR spectrum of compound **4d** (101 MHz, $CDCl_3$)

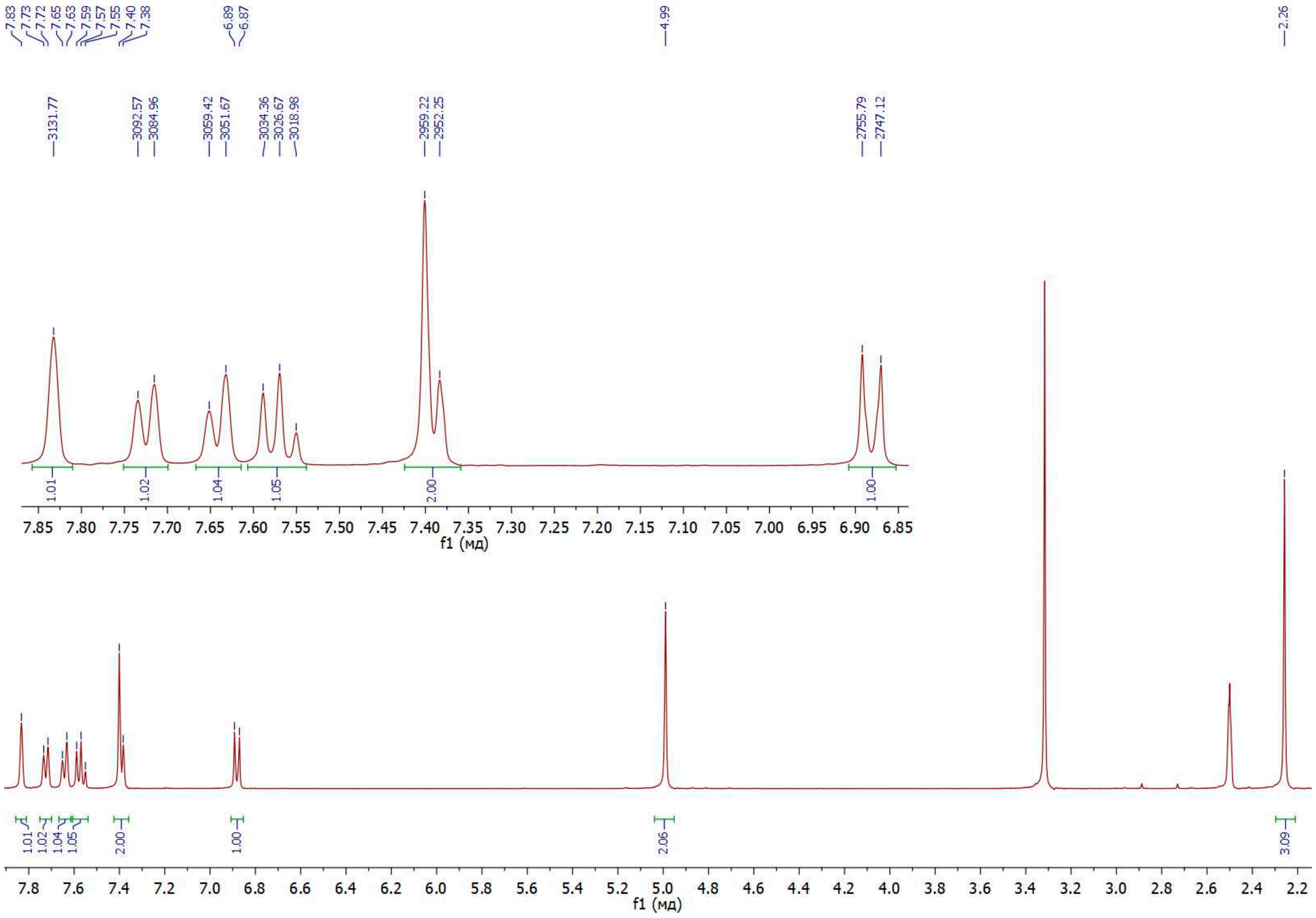

Figure S32. ^{1}H NMR spectrum of compound **4e** (500 MHz, DMSO-d_6)

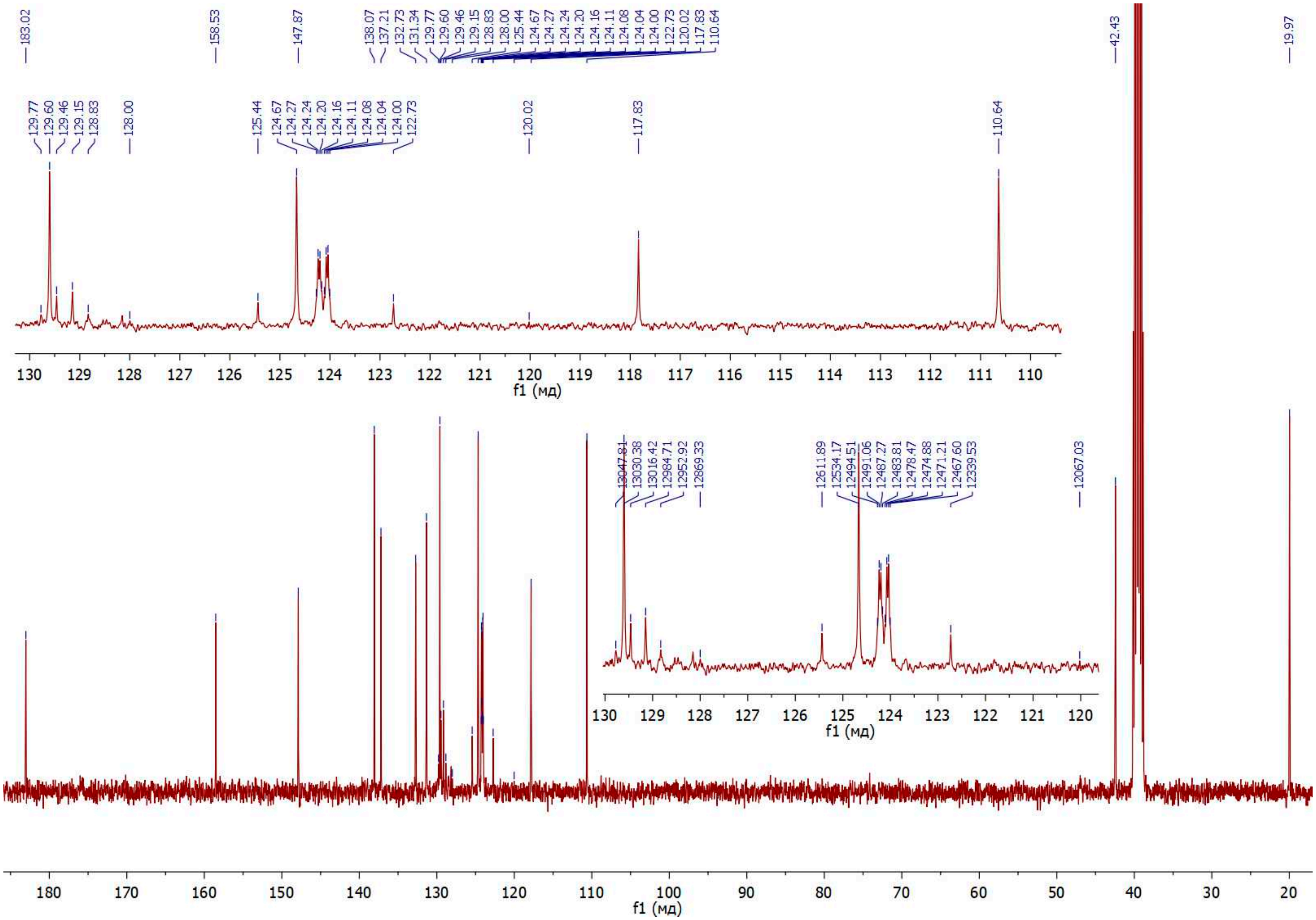

Figure S33. ^{13}C NMR spectrum of compound **4e** (126 MHz, DMSO-d_6)

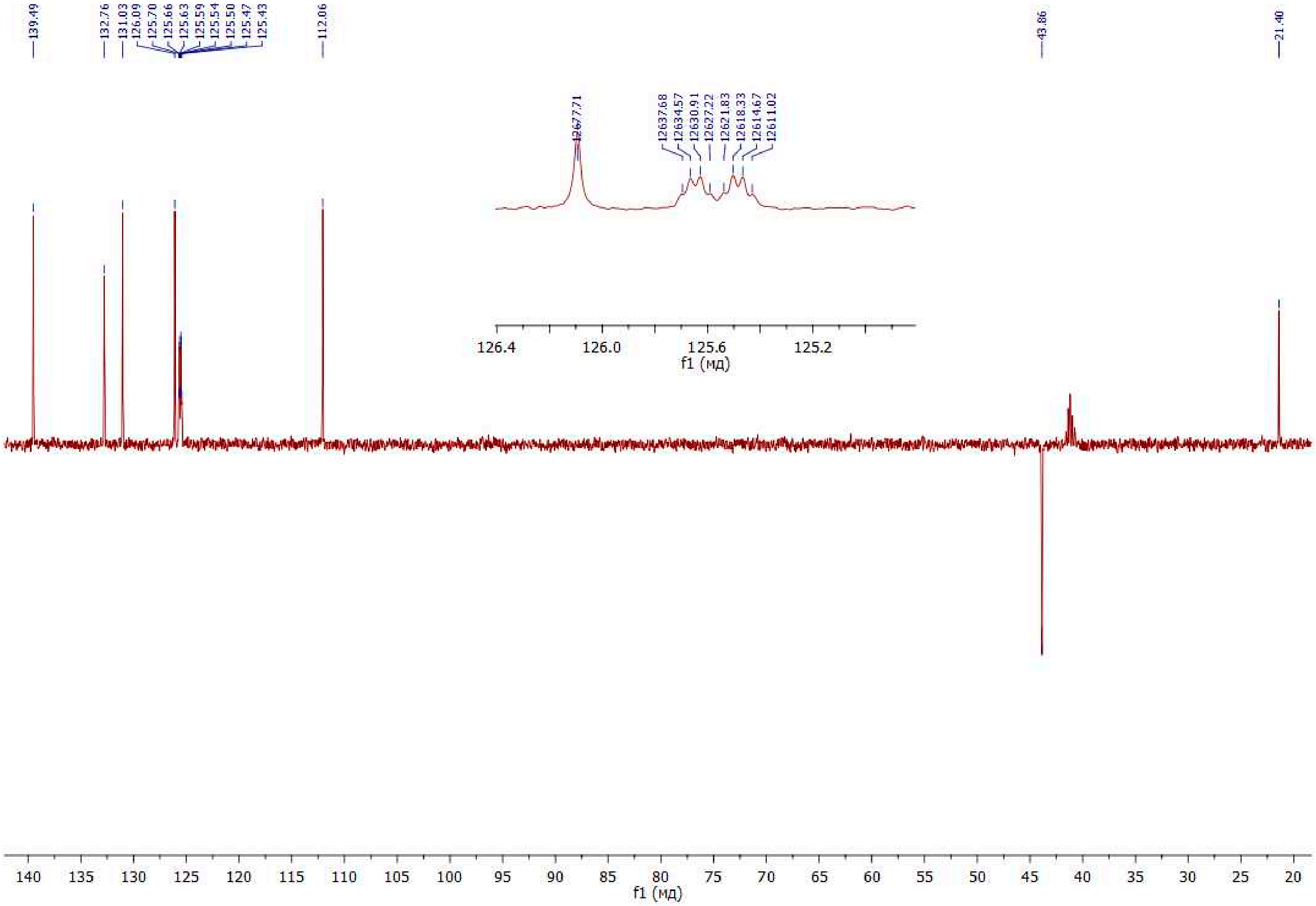

Figure S34. ^{13}C (dept) NMR spectrum of compound **4e** (126 MHz, DMSO-d_6)

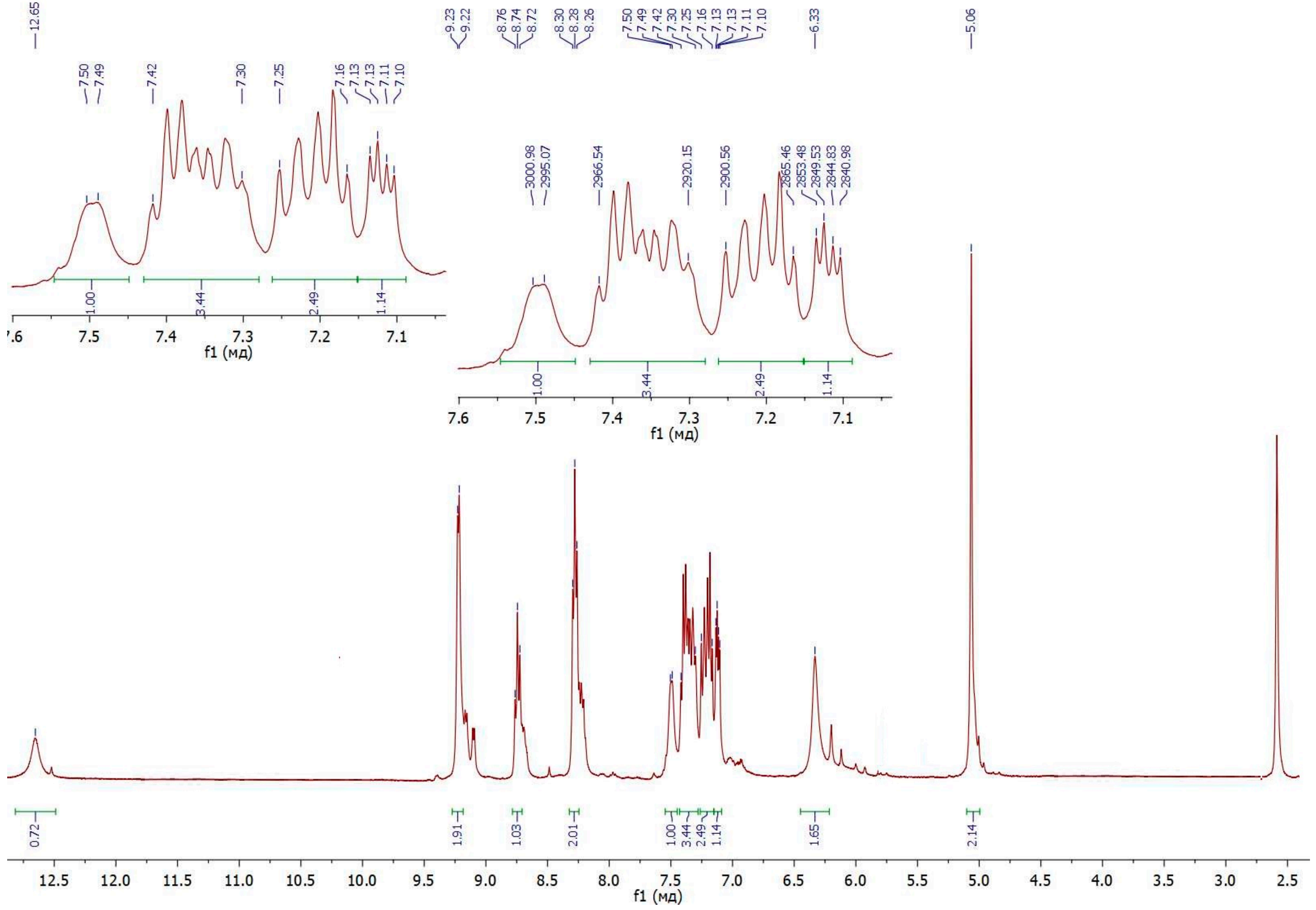

Figure S35. ^{1}H NMR spectrum of compound **5a** (500 MHz, DMSO-d_6)

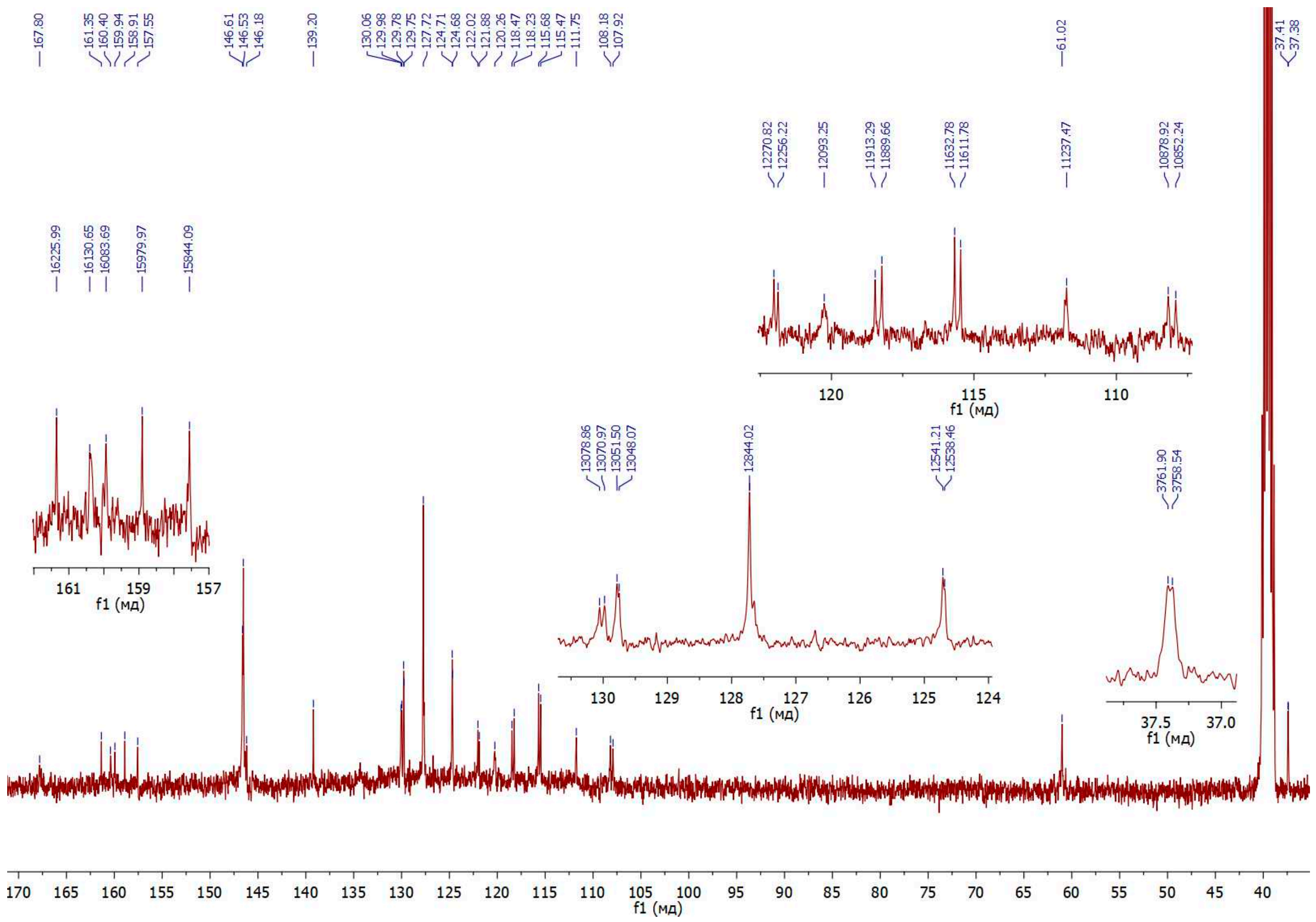

Figure S36. ^{13}C NMR spectrum of compound **5a** (126 MHz, DMSO-d_6)

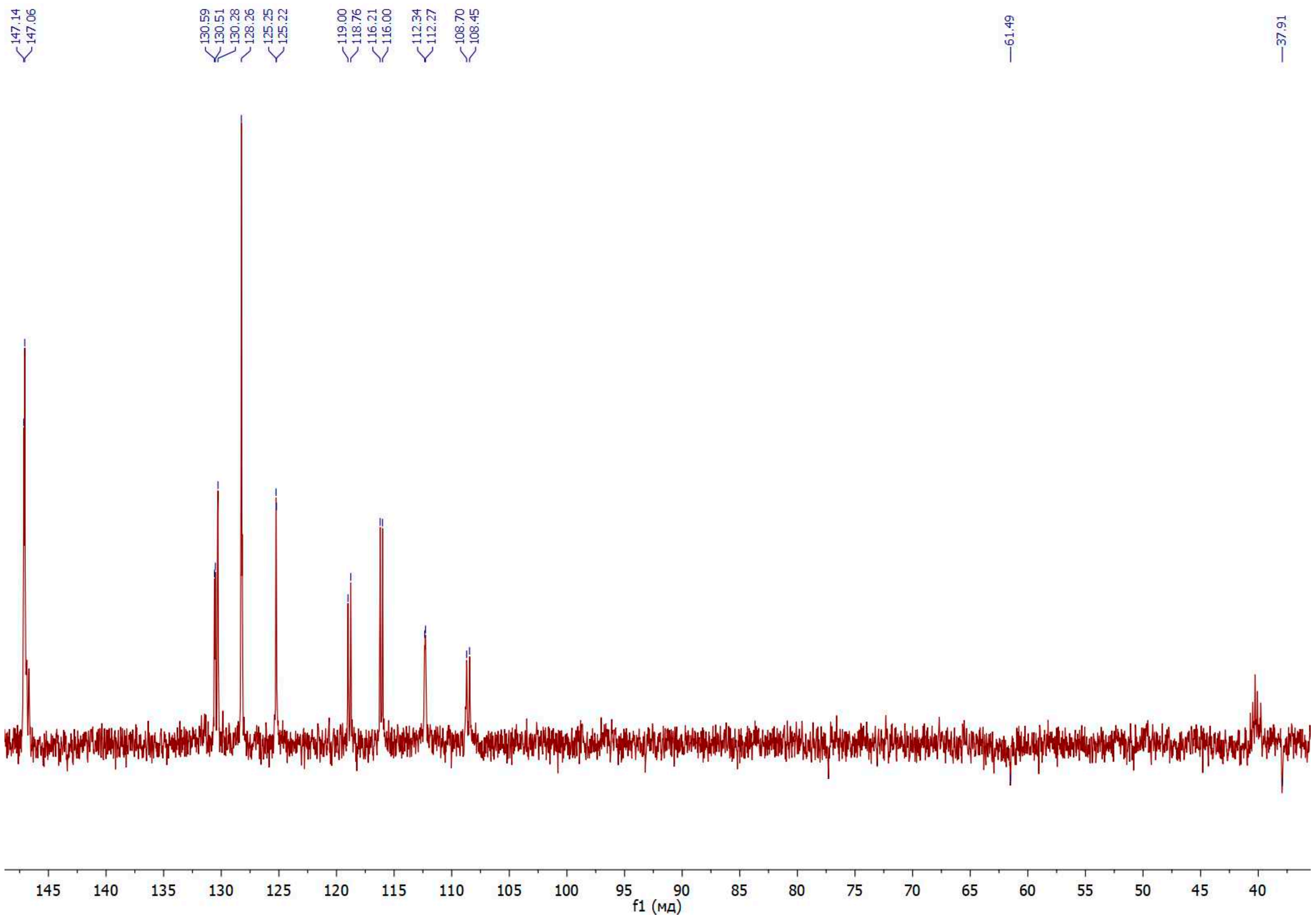

Figure S37. ^{13}C (dept) NMR spectrum of compound **5a** (126 MHz, DMSO-d_6)

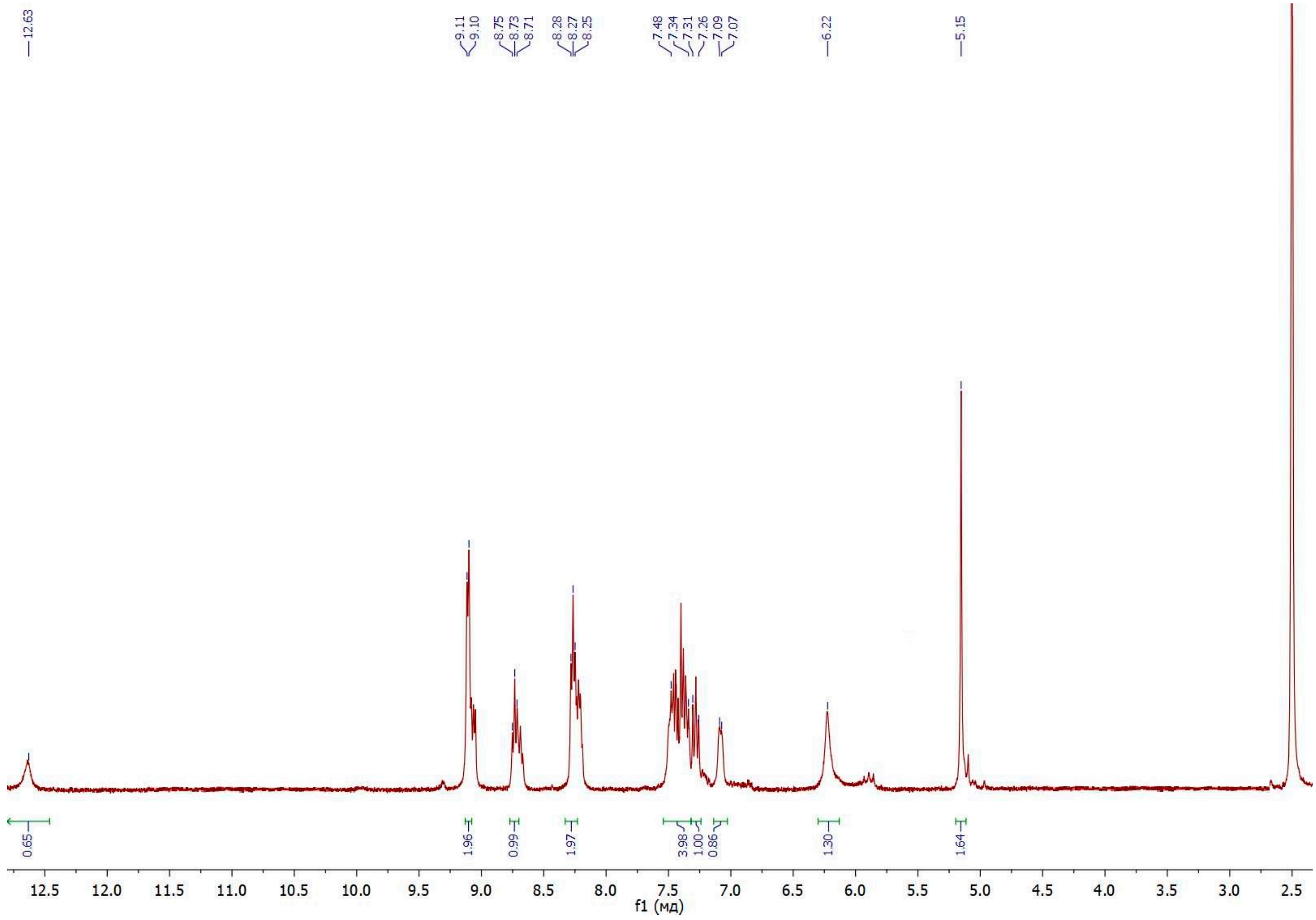

Figure S38. ^{1}H NMR spectrum of compound **5b** (500 MHz, DMSO-d_6)

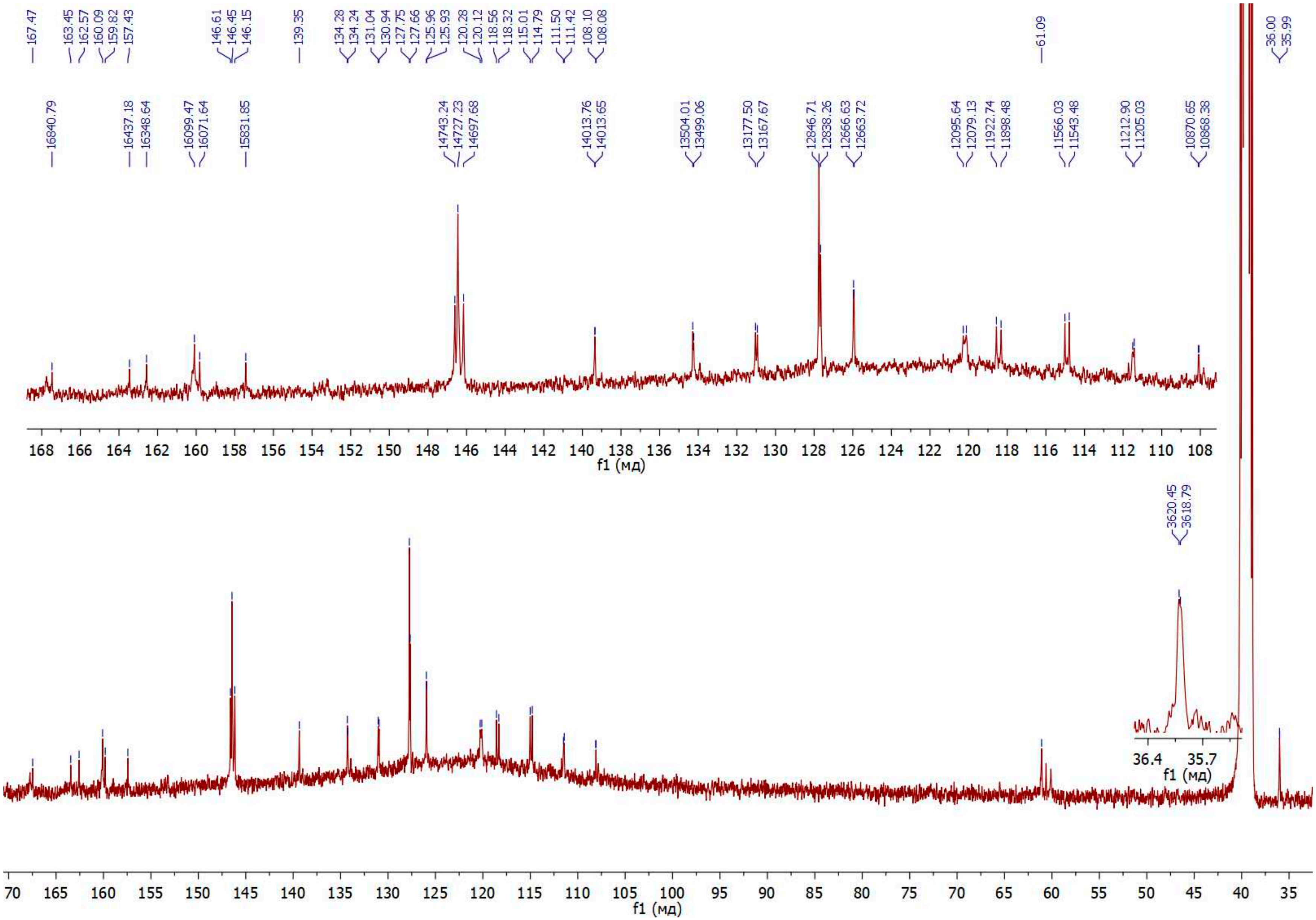

Figure S39. ^{13}C NMR spectrum of compound **5b** (126 MHz, DMSO-d_6)

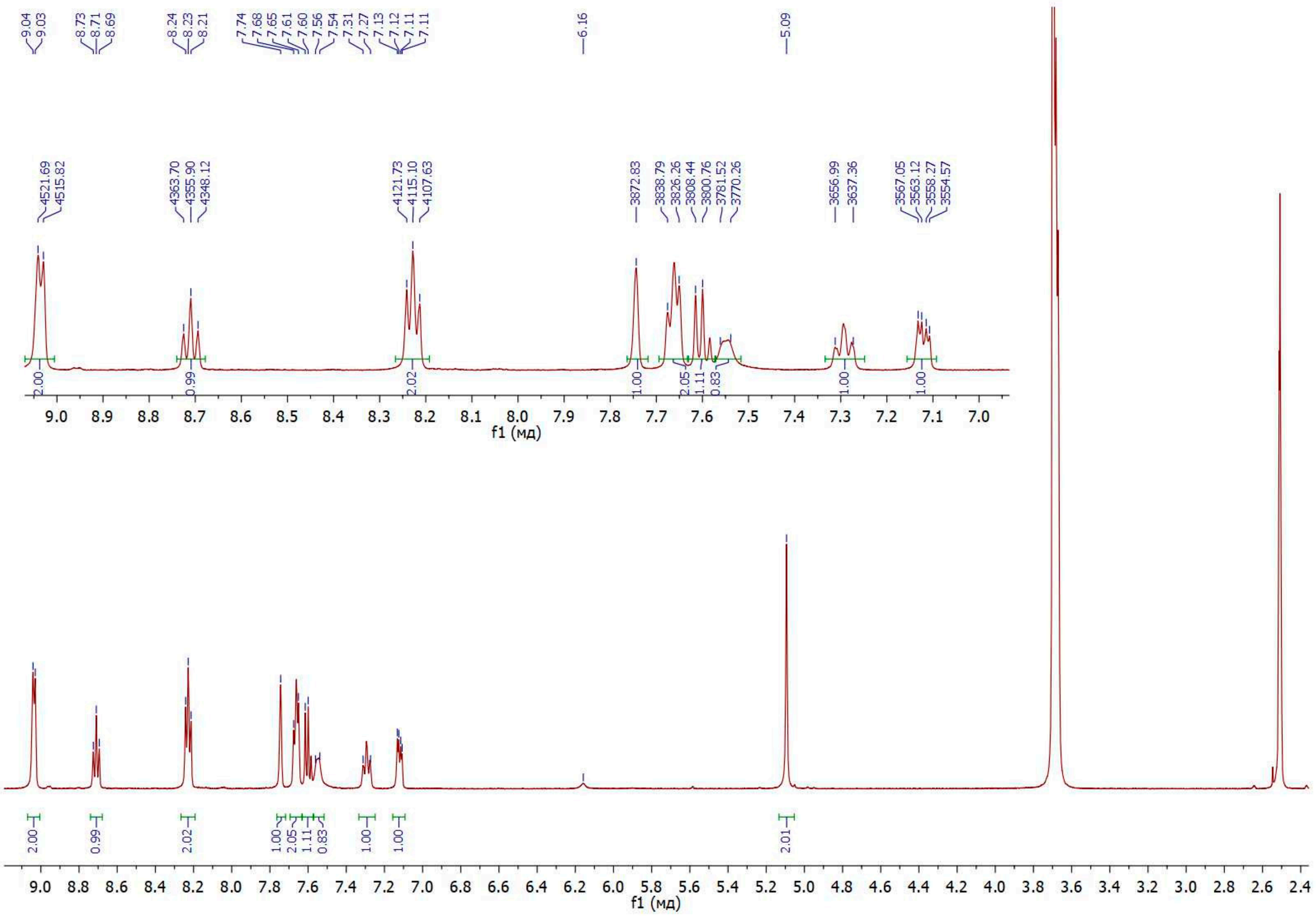

Figure S40. ^{1}H NMR spectrum of compound **5c** (500 MHz, DMSO-d_6)

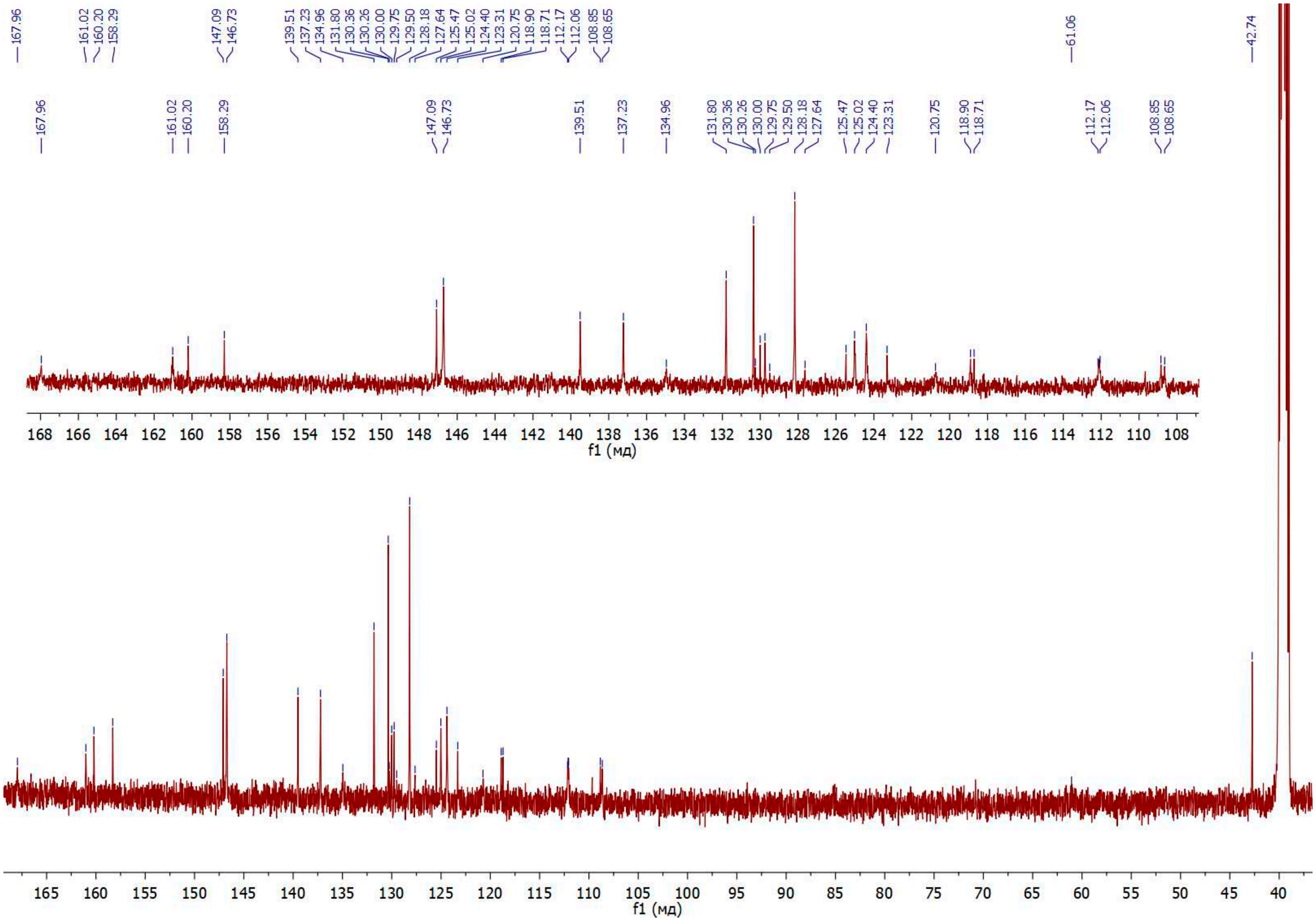

Figure S41. ^{13}C NMR spectrum of compound **5c** (126 MHz, DMSO-d_6)

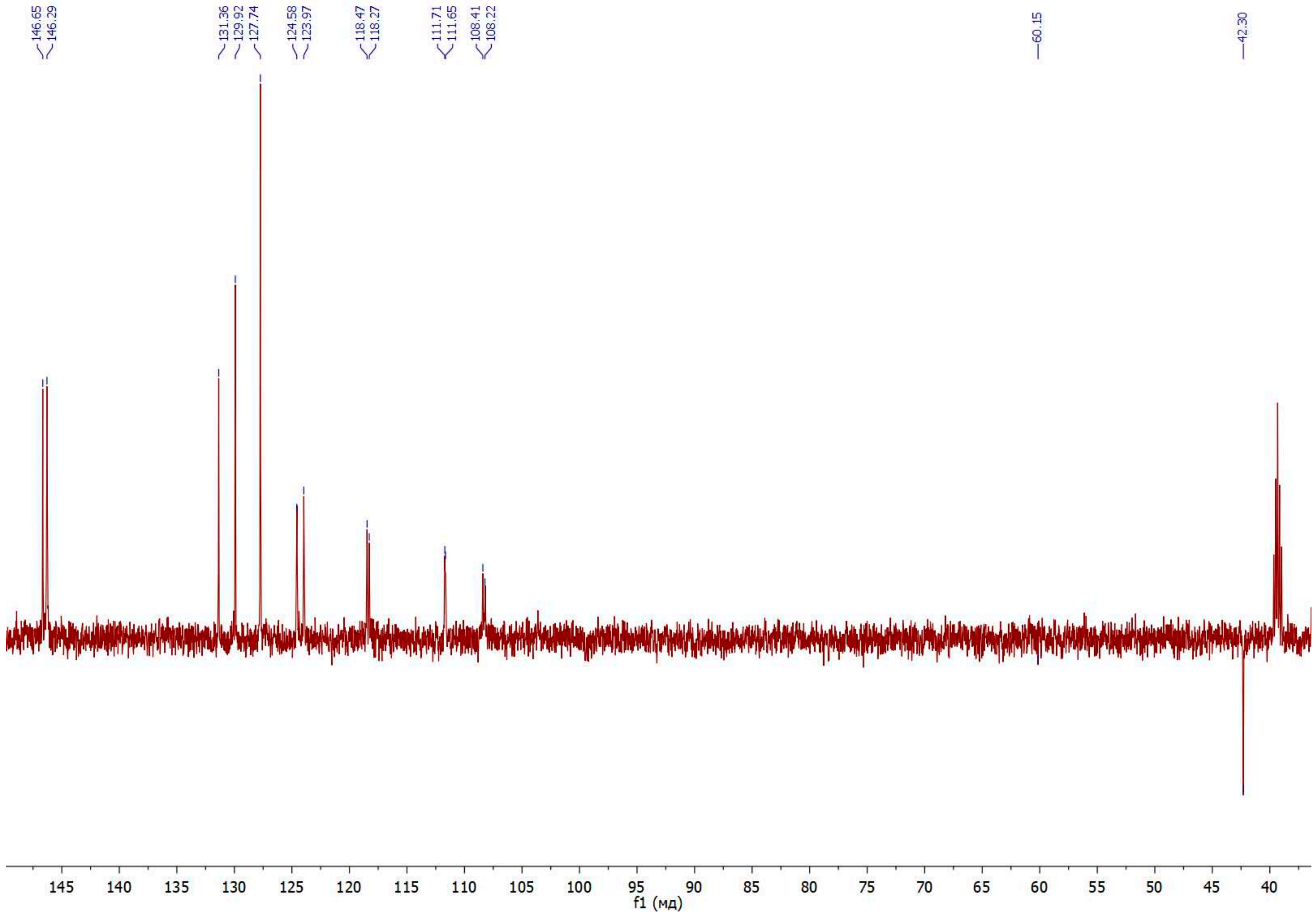

Figure S42. ^{13}C (dept) NMR spectrum of compound **5c** (126 MHz, DMSO-d_6)

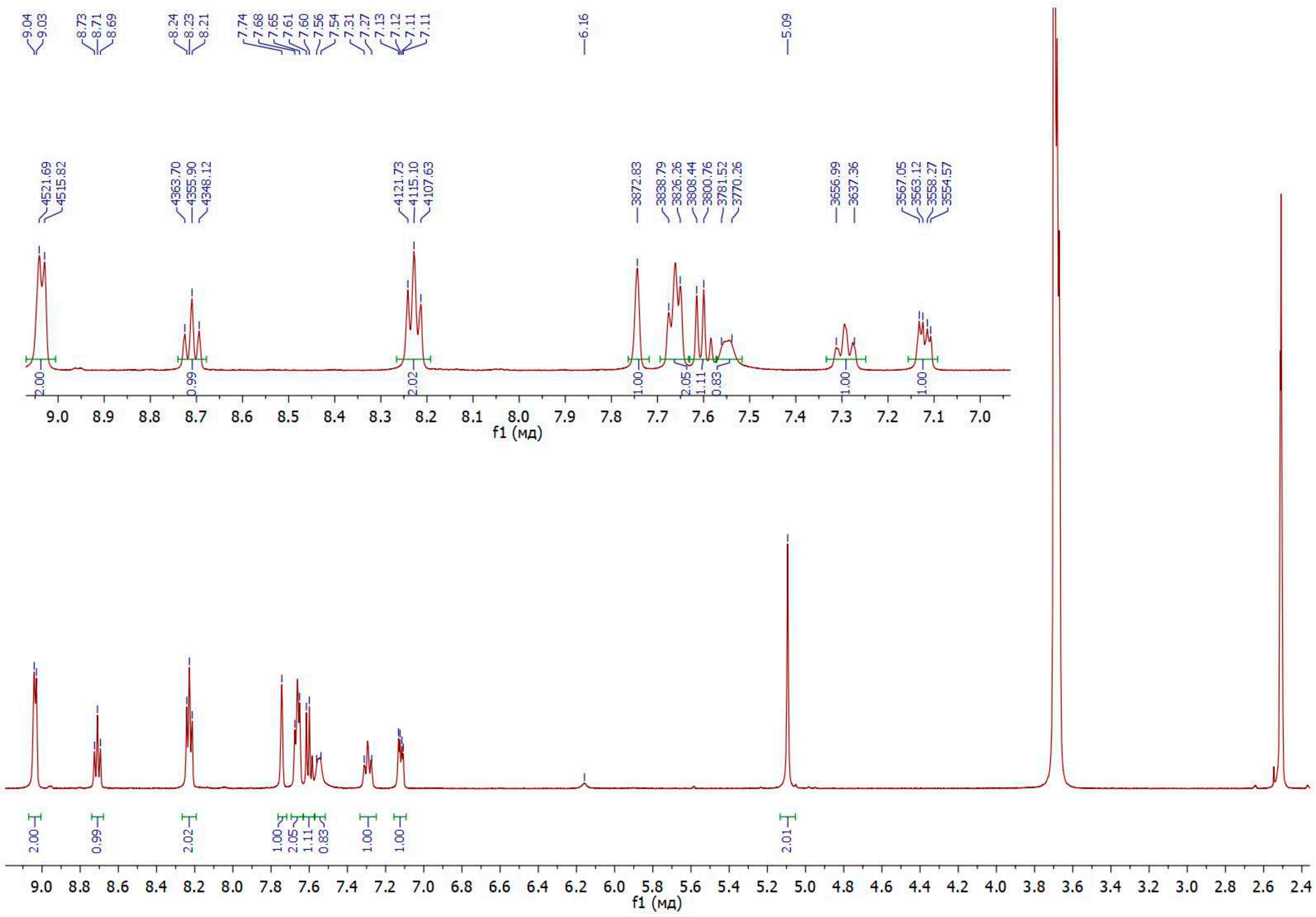

Figure S43. ^{1}H NMR spectrum of compound **5d** (600 MHz, DMSO-d_6)

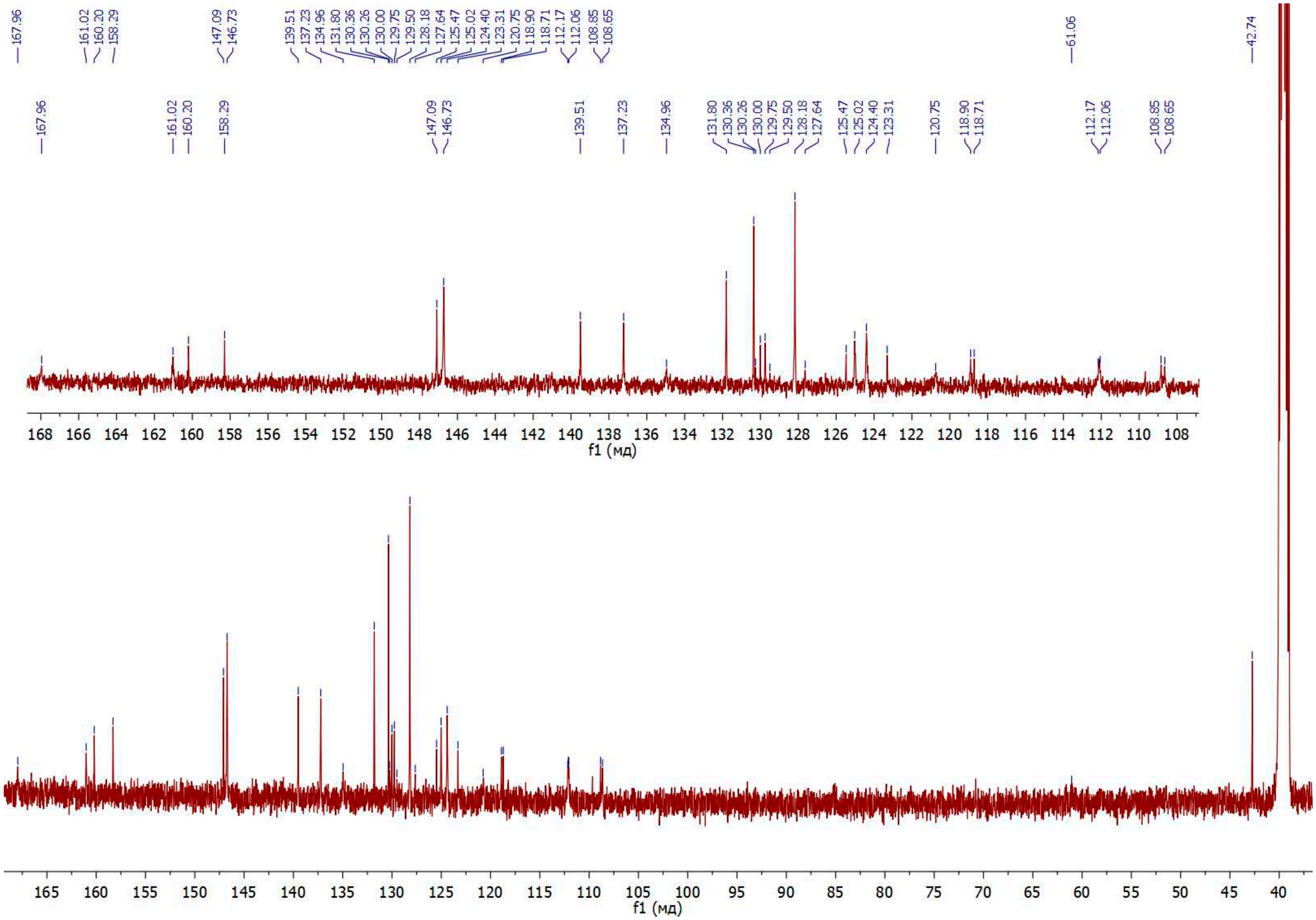

Figure S44. ^{13}C NMR spectrum of compound **5d** (151 MHz, DMSO-d_6)

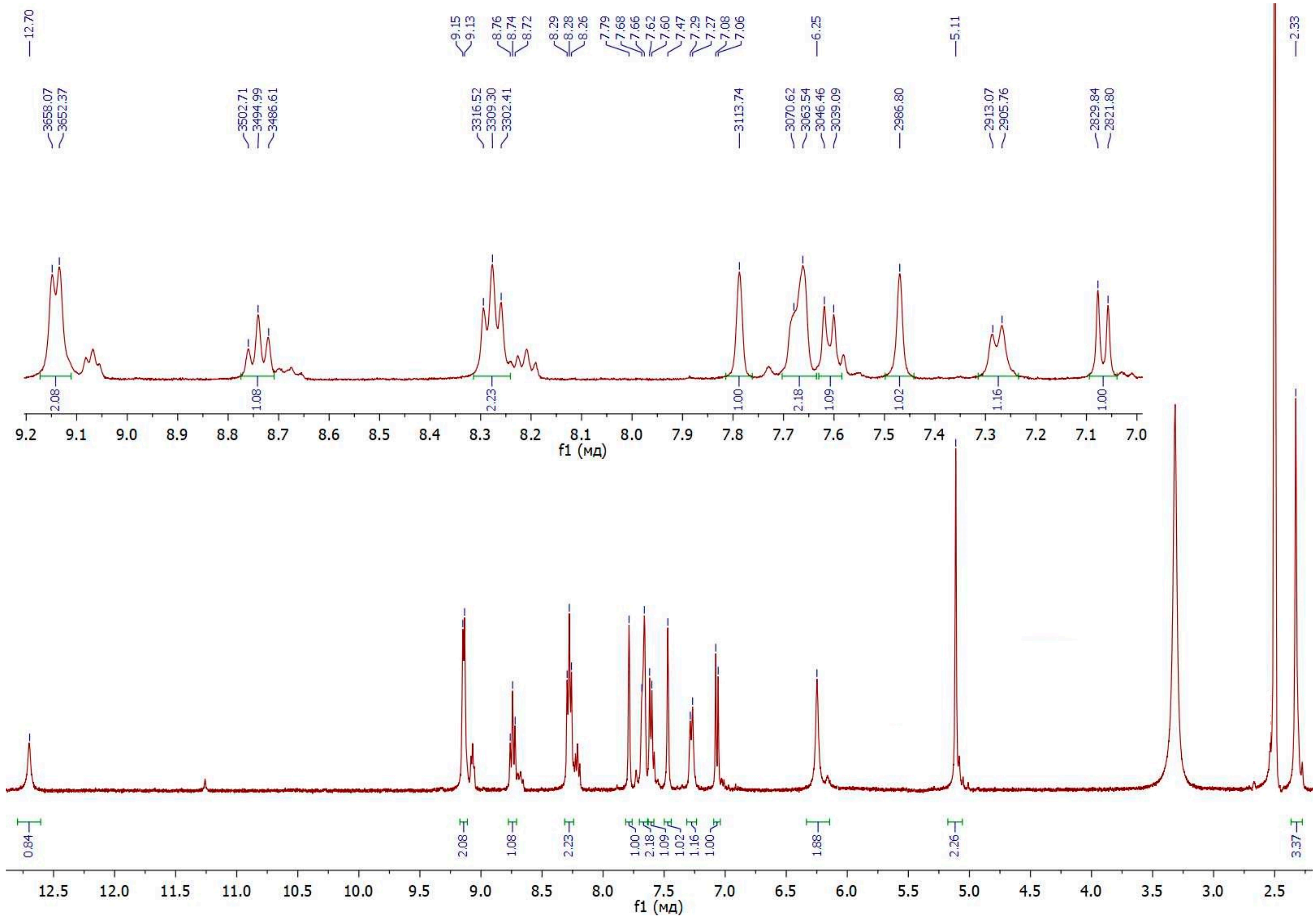

Figure S45. ^1H NMR spectrum of compound **5e** (500 MHz, DMSO-d_6)

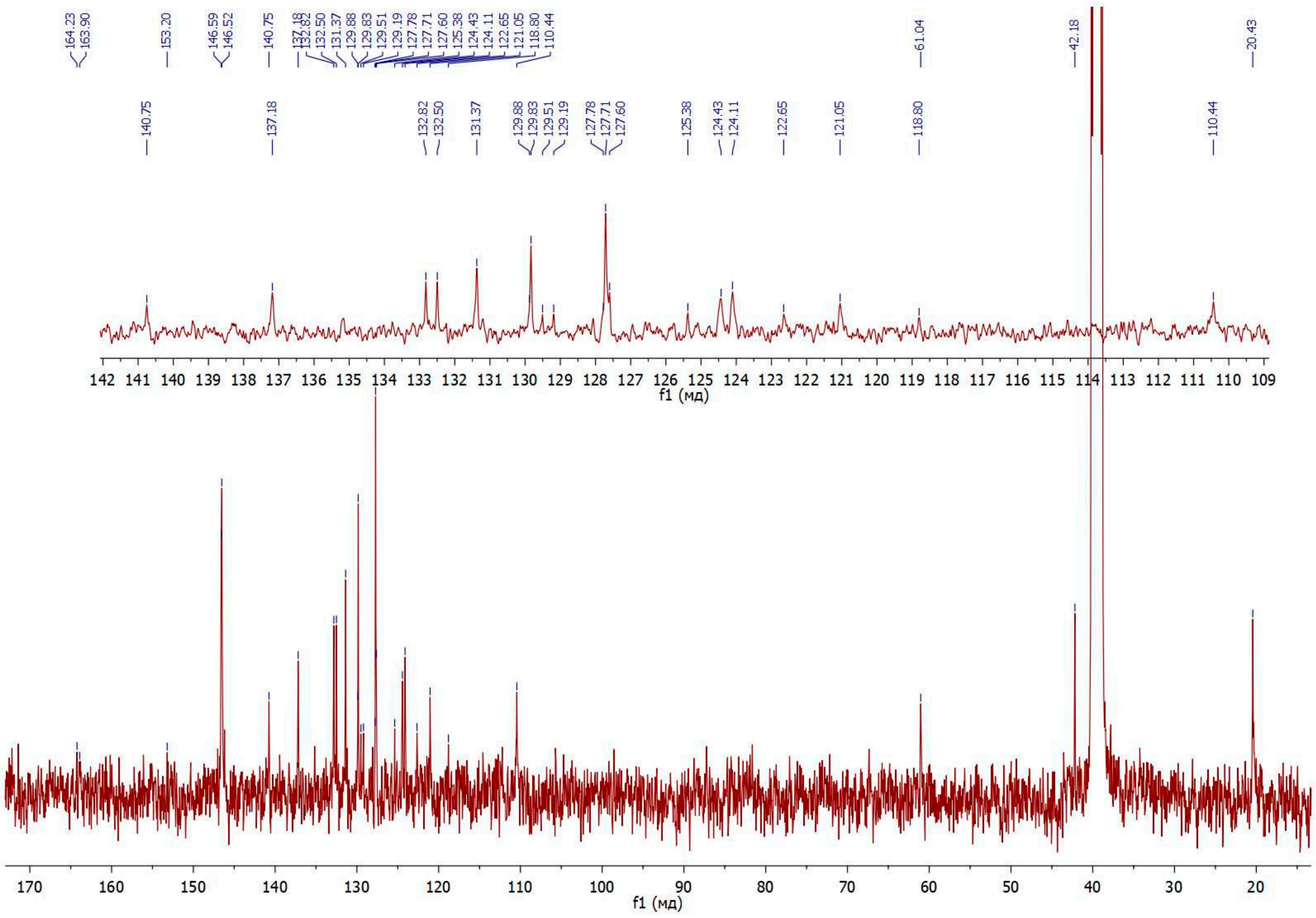

Figure S46. ^{13}C NMR spectrum of compound **5e** (126 MHz, DMSO-d_6)

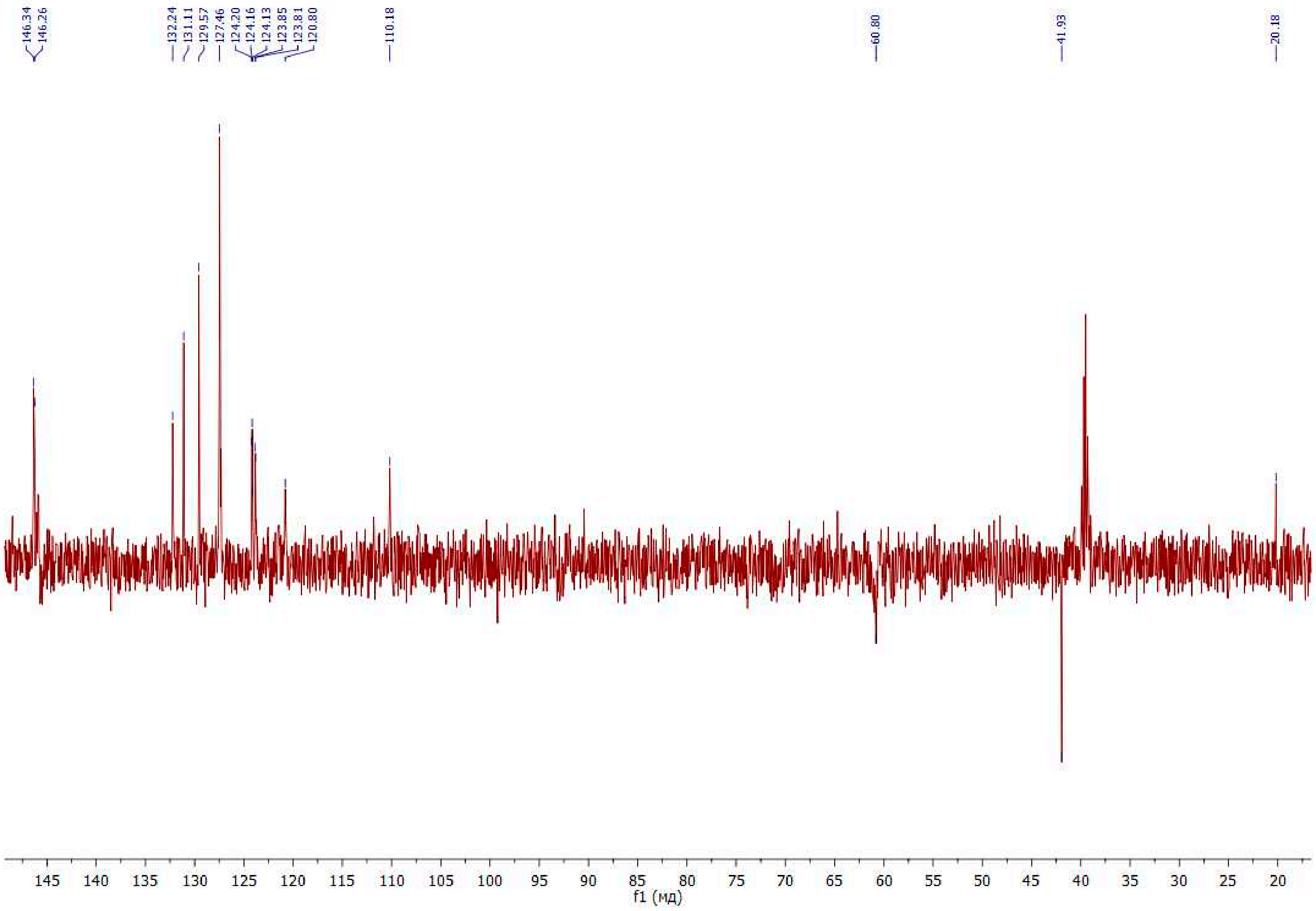

Figure S47. ^{13}C (dept) NMR spectrum of compound **5e** (126 MHz, DMSO-d_6)

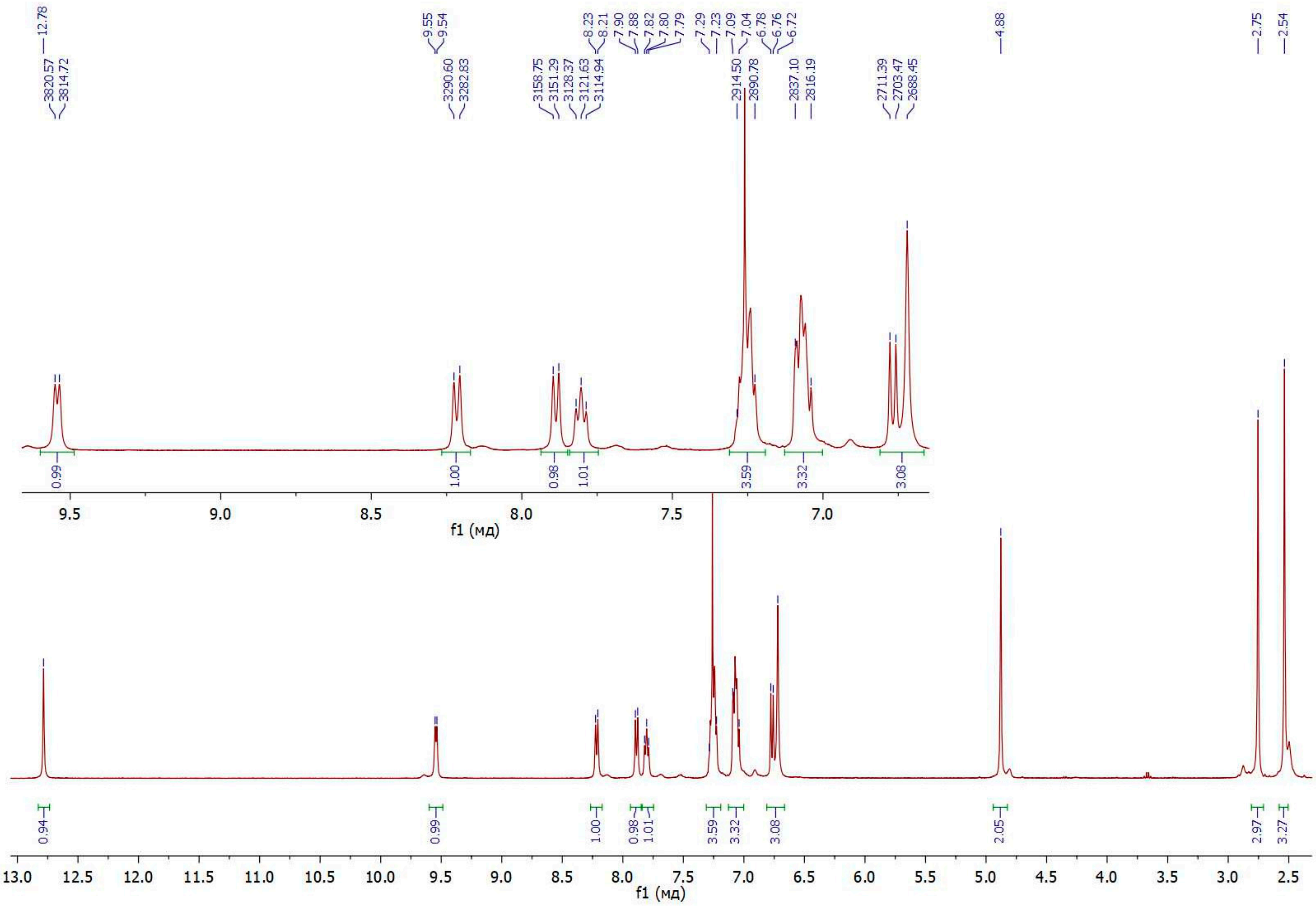

Figure S48. ^{1}H NMR spectrum of compound **7a** (600 MHz, $CDCl_3$)

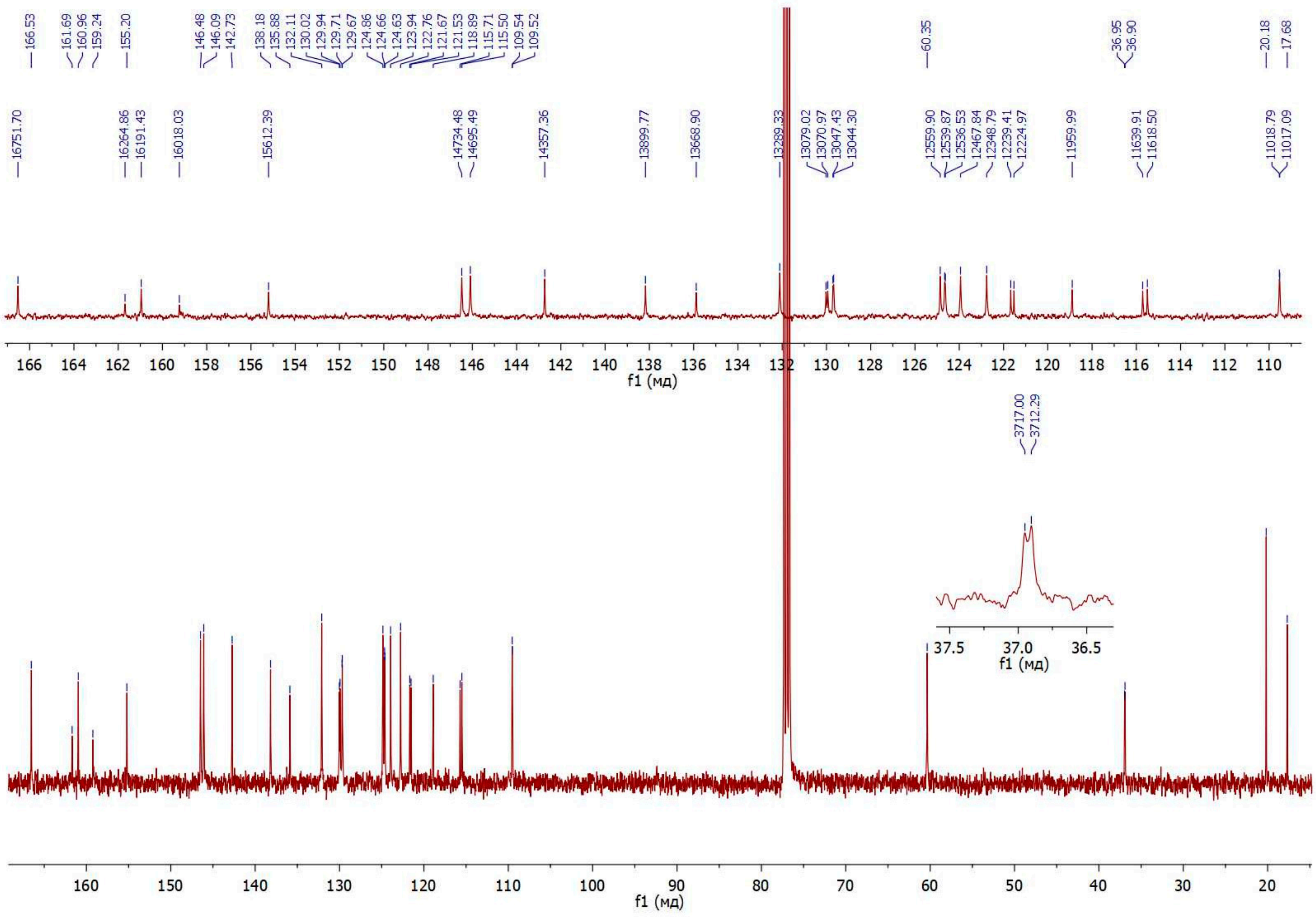

Figure S49. ^{13}C NMR spectrum of compound **7a** (151 MHz, $CDCl_3$)

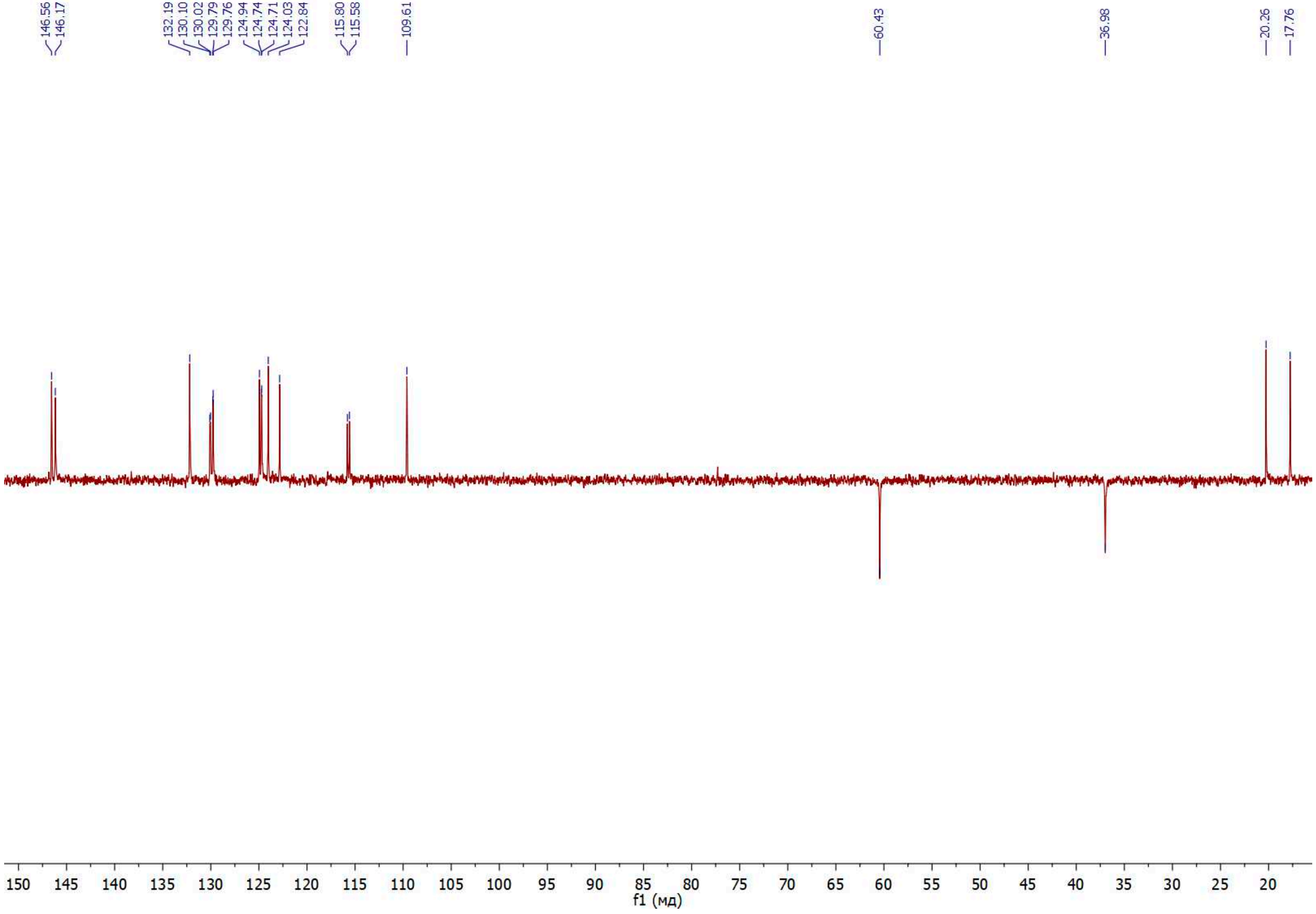

Figure S50. ^{13}C (dept) NMR spectrum of compound **7a** (151 MHz, $CDCl_3$)

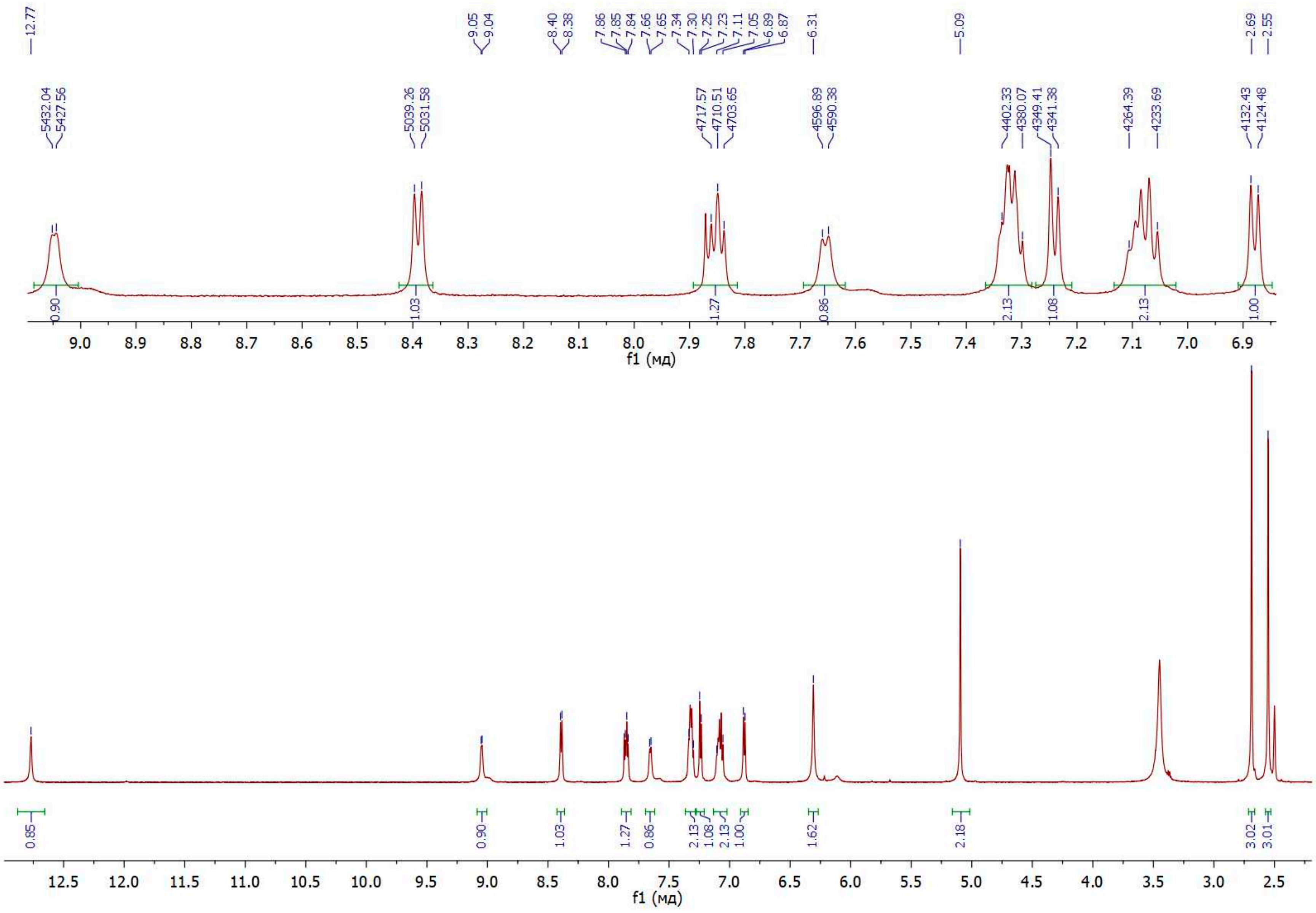

Figure S51. ^{1}H NMR spectrum of compound **7b** (600 MHz, $CDCl_3$/DMSO-d_6)

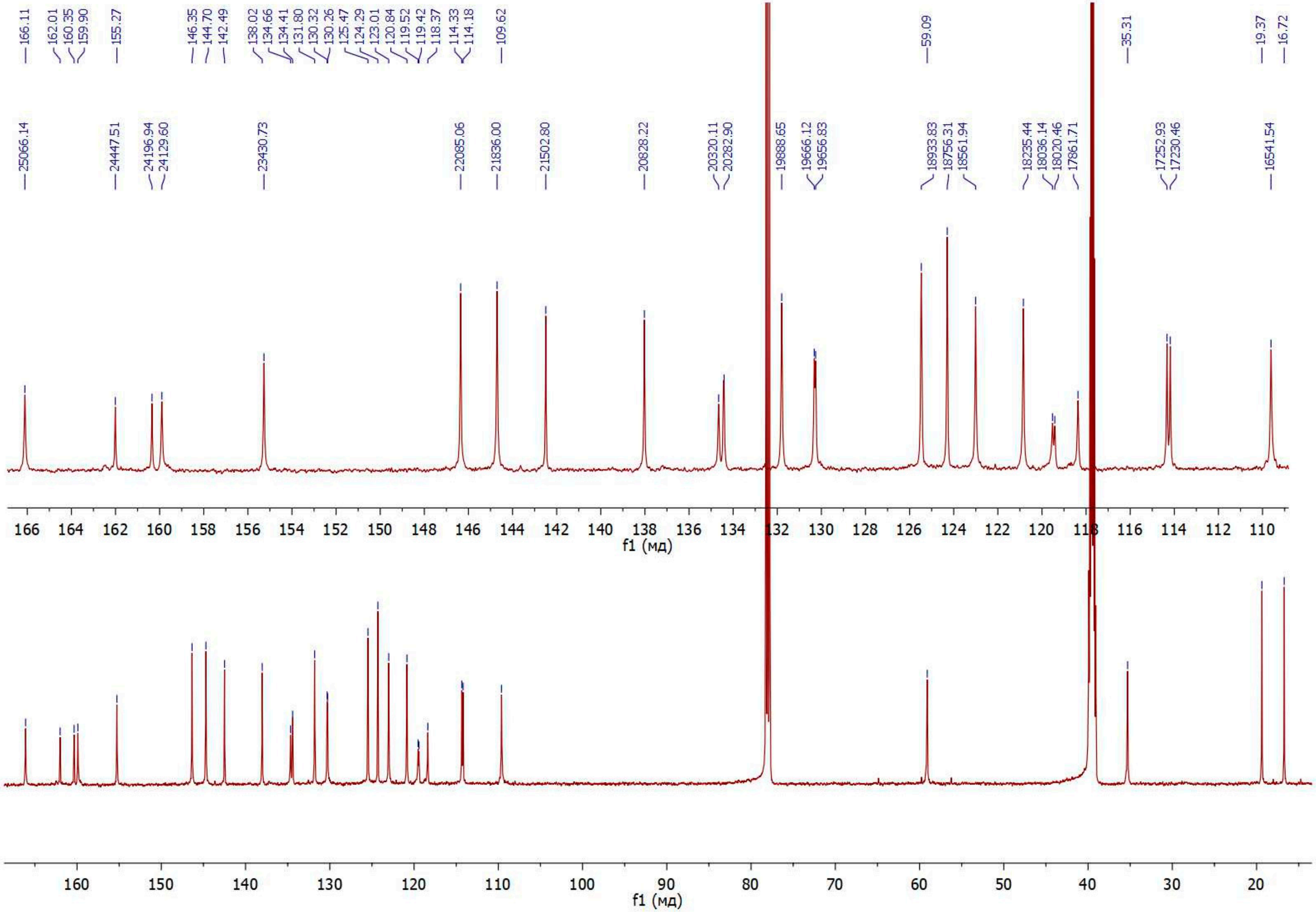

Figure S52. ^{13}C NMR spectrum of compound **7b** (151 MHz, $CDCl_3$/DMSO-d_6)

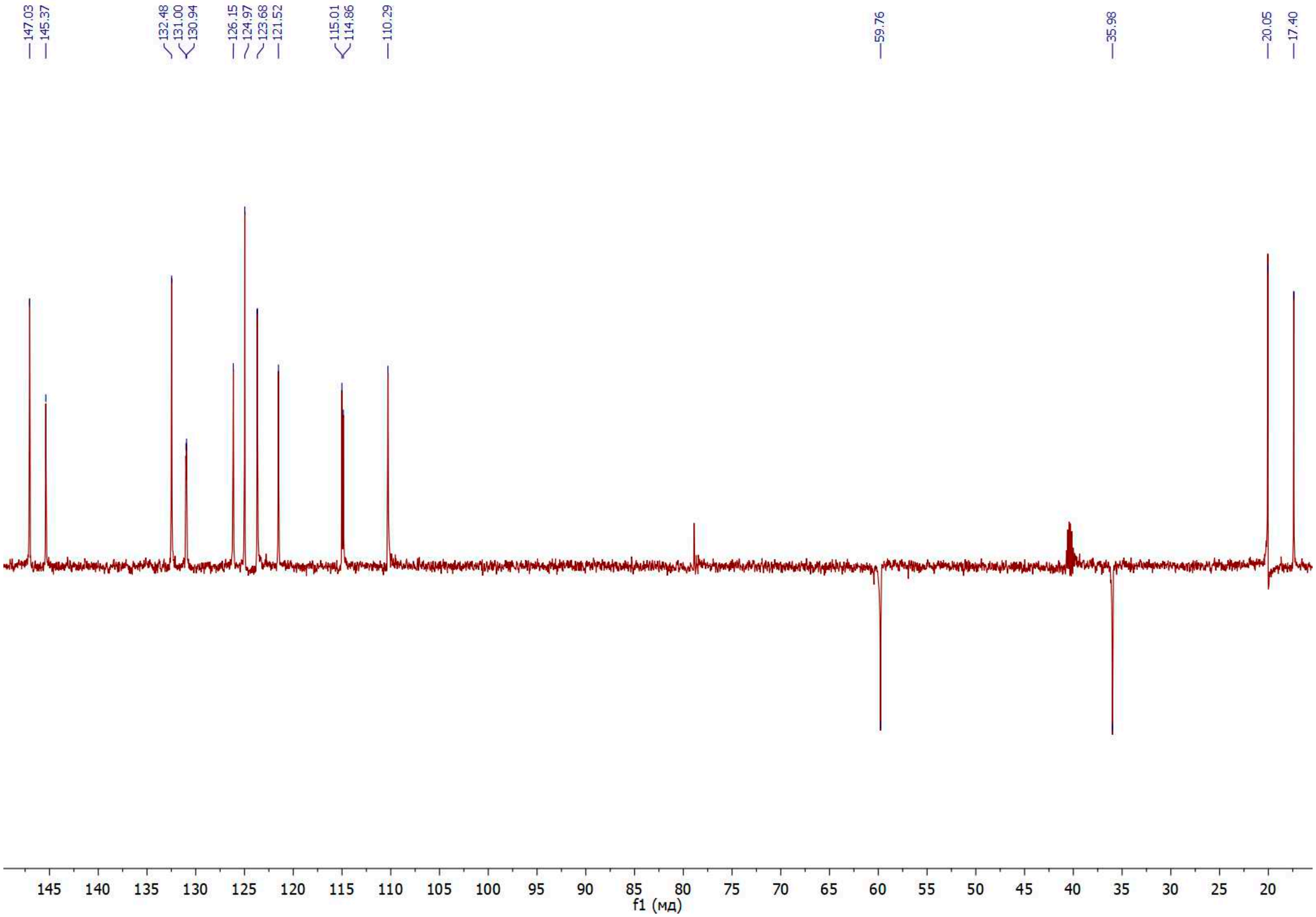

Figure S53. ^{13}C (dept) NMR spectrum of compound **7b** (151 MHz, $CDCl_3$/DMSO-d_6)

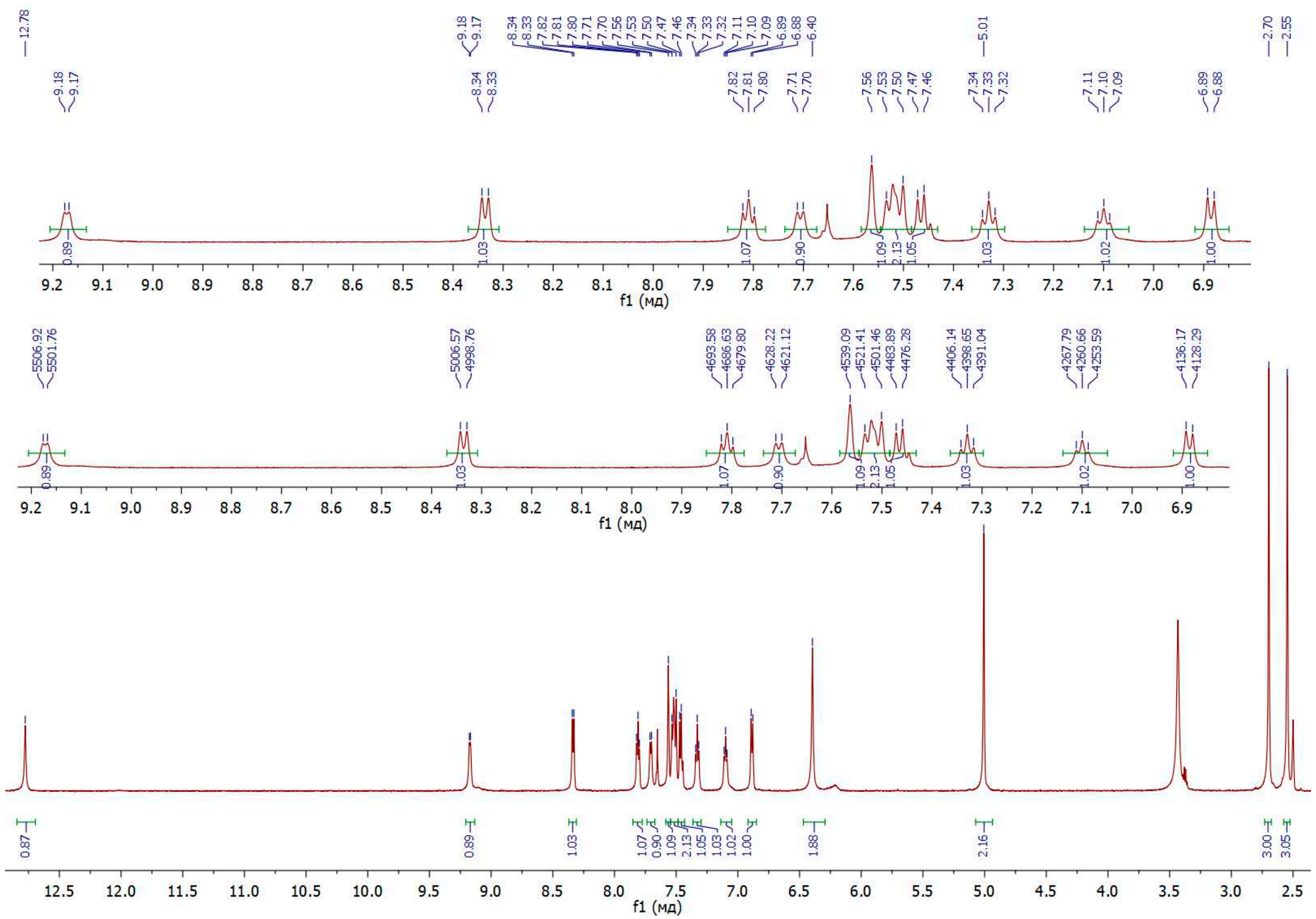

Figure S54. ^{1}H NMR spectrum of compound **7c** (400 MHz, $CDCl_3$/DMSO-d_6)

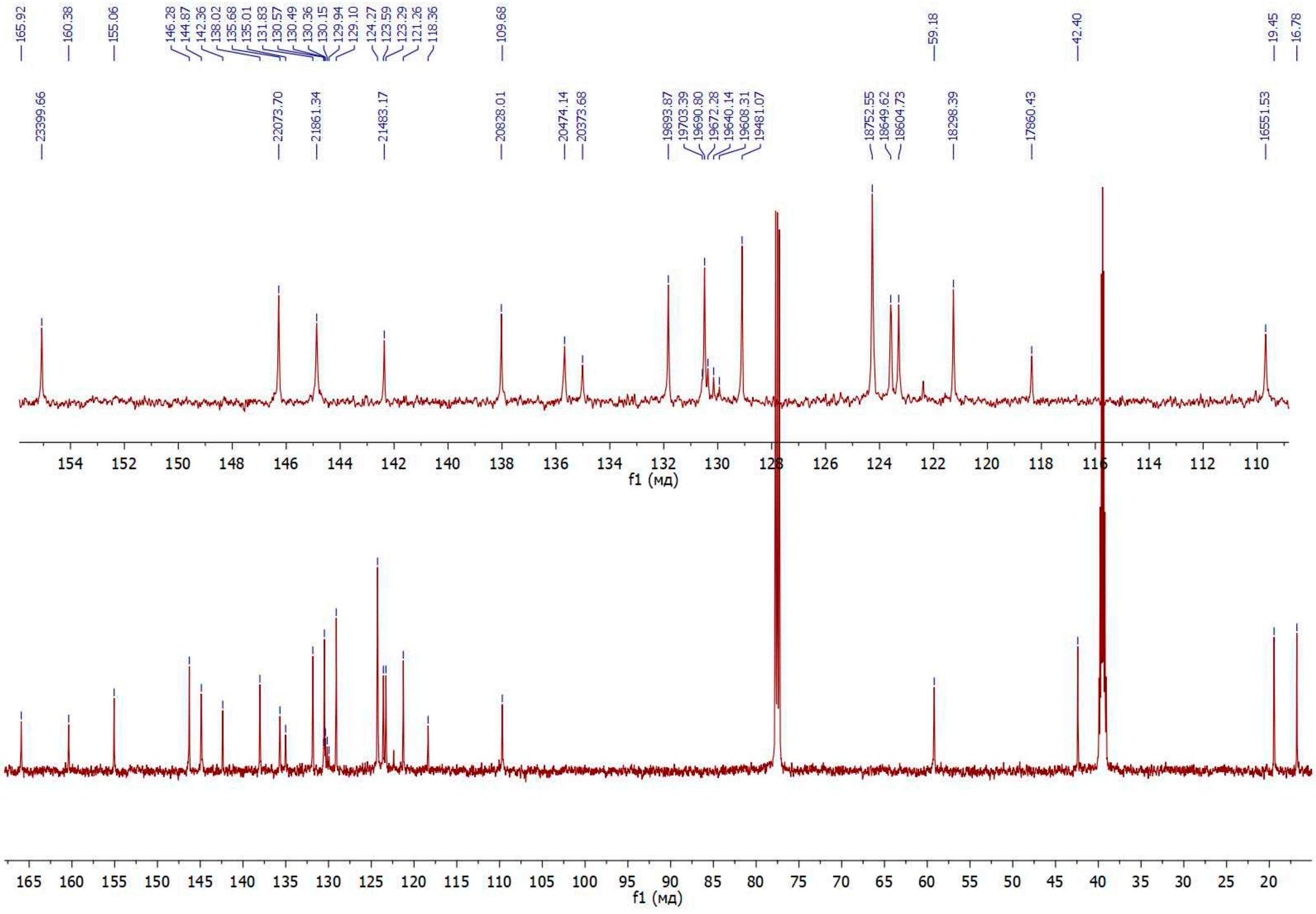

Figure S55. ^{13}C NMR spectrum of compound **7c** (101 MHz, $CDCl_3$/DMSO-d_6)

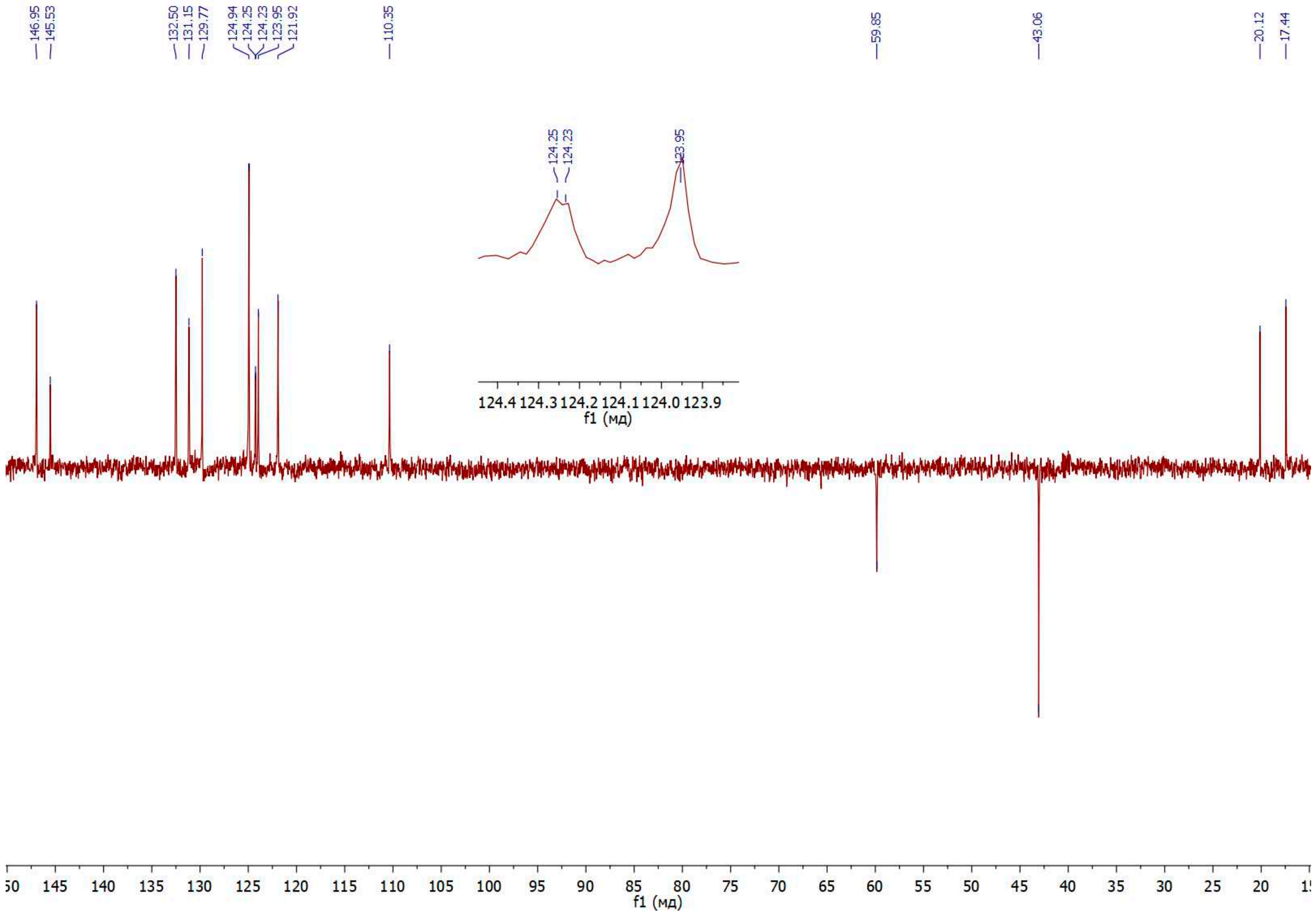

Figure S56. ^{13}C (dept) NMR spectrum of compound **7c** (101 MHz, $CDCl_3$/DMSO-d_6)

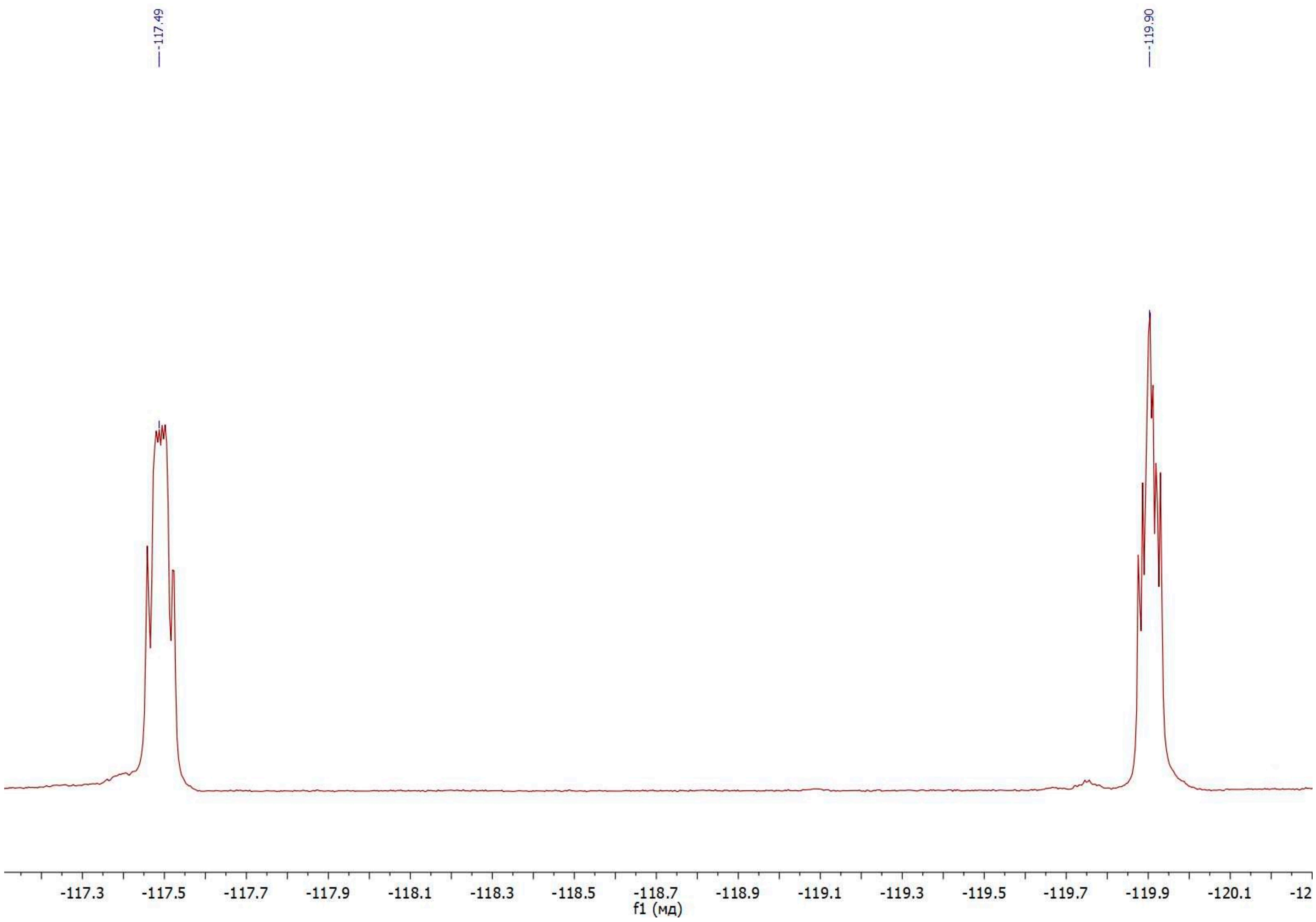

Figure S57. ^{19}F NMR spectrum of compound **3a** (377 MHz, DMSO-d_6)

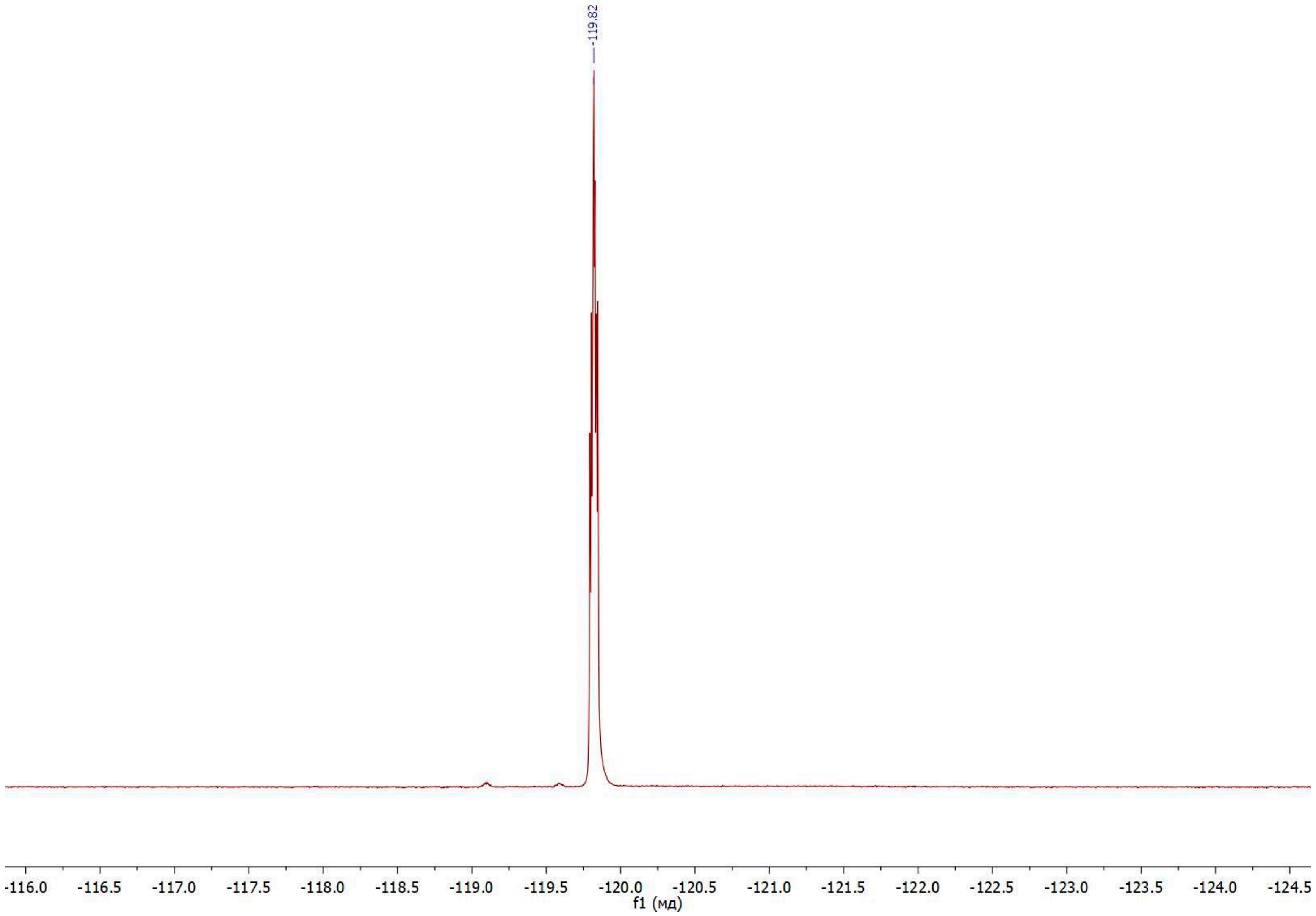

Figure S58. ^{19}F NMR spectrum of compound **3b** (377 MHz, DMSO-d_6)

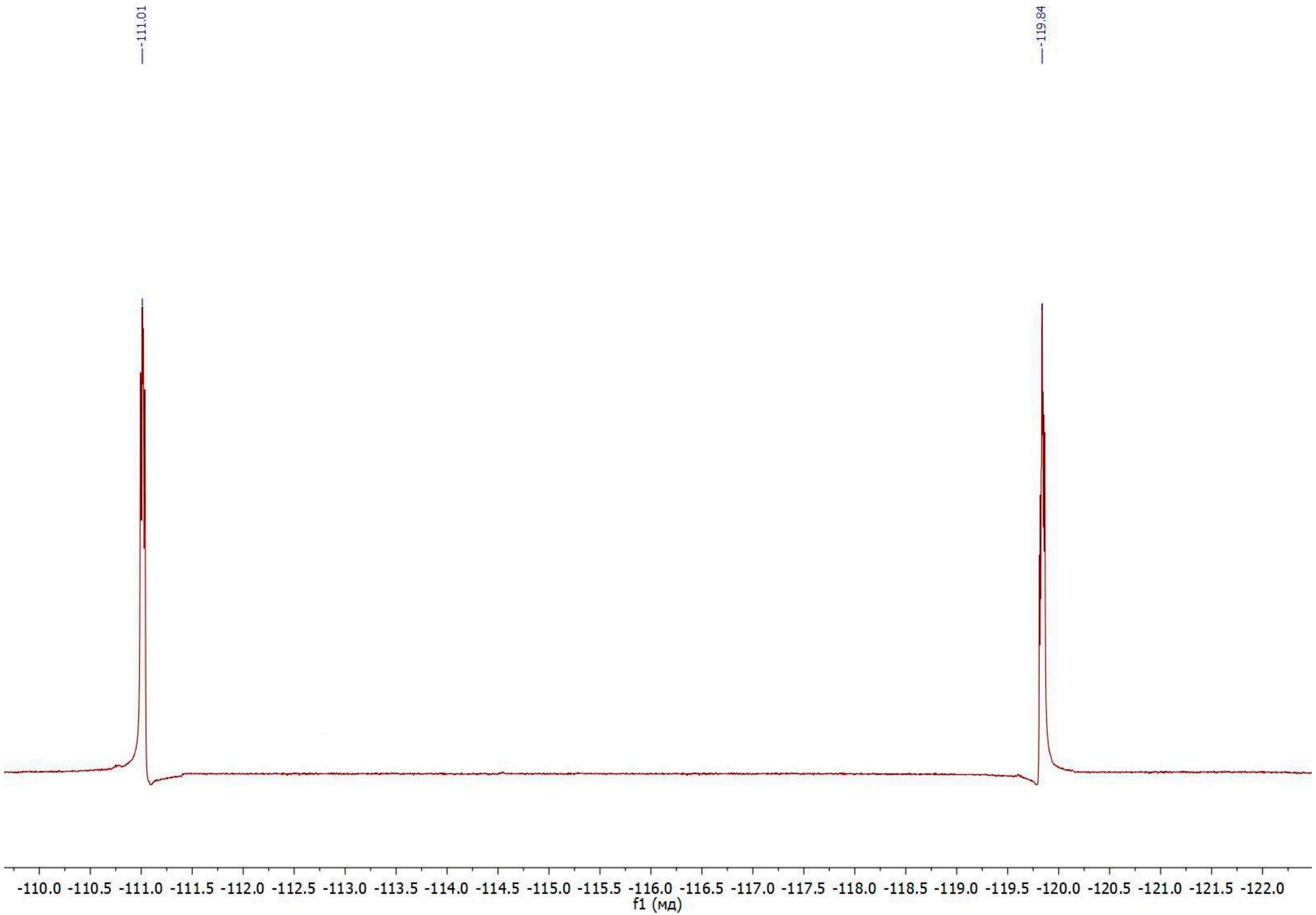

Figure S59. ^{19}F NMR spectrum of compound **3d** (377 MHz, DMSO-d_6)

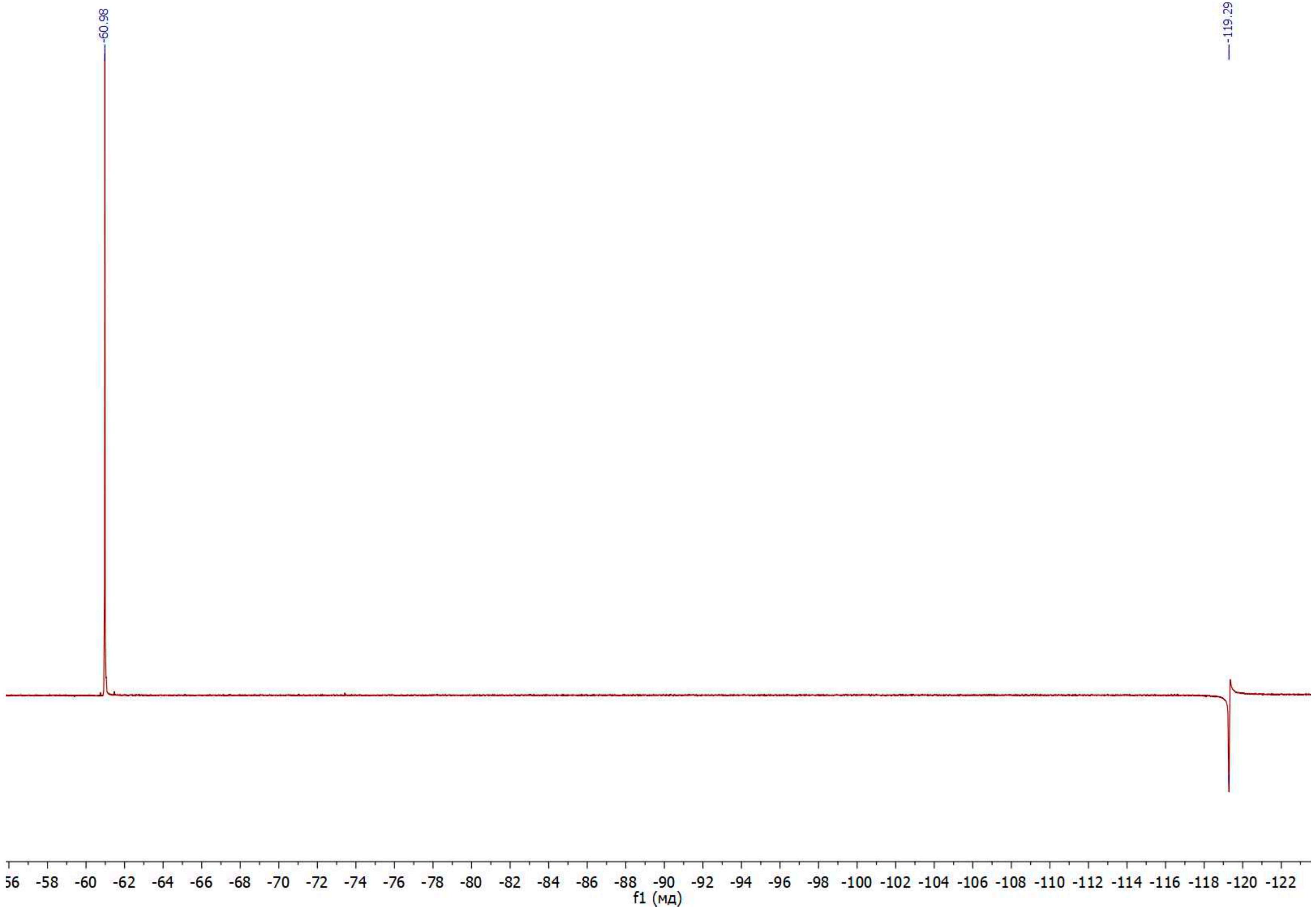

Figure S60. ^{19}F NMR spectrum of compound **5c** (377 MHz, DMSO-d_6)

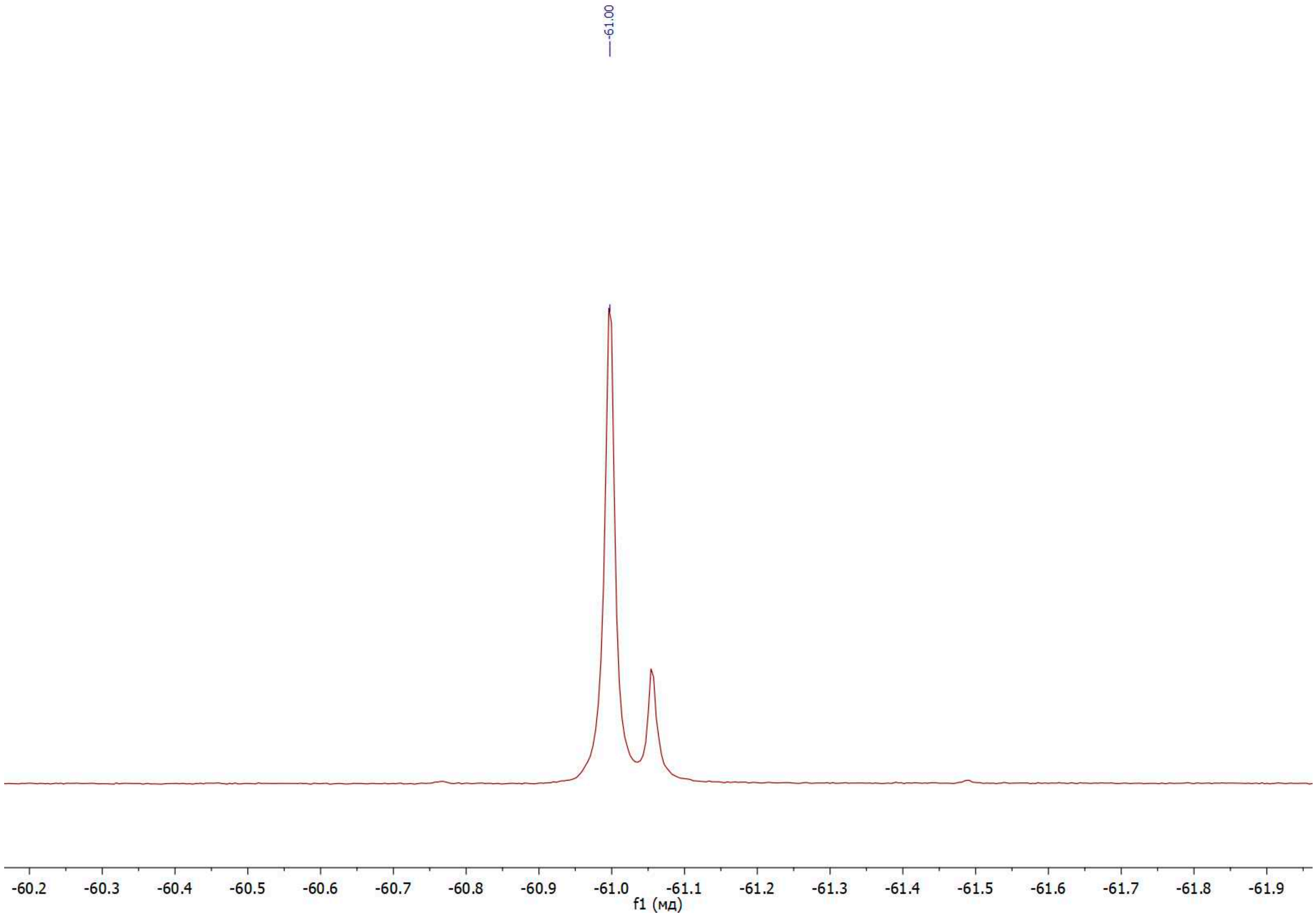

Figure S61. ^{19}F NMR spectrum of compound **5e** (377 MHz, DMSO-d_6)

Compd	IC50 (μM)			
	Cancer cell line		ChangLiver	WI38
	M-HeLa	HuTu 80		
**3a**	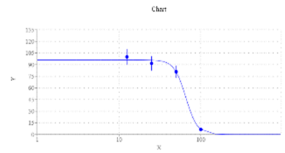	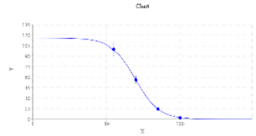	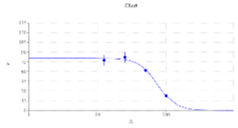	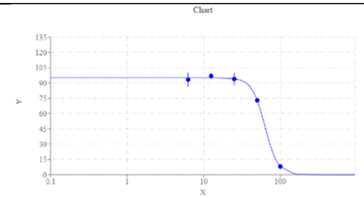
**3b**	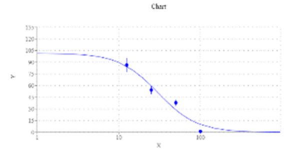	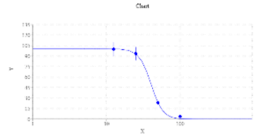	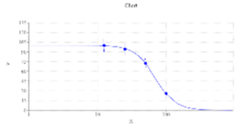	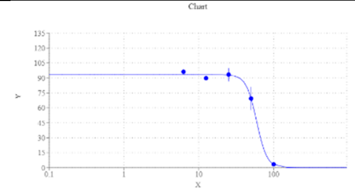
**3c**	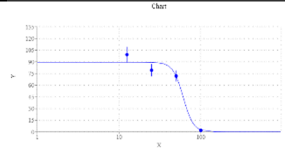	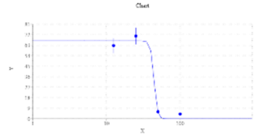	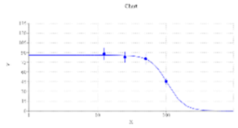	
**3d**	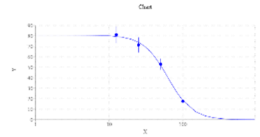	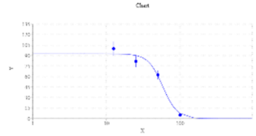	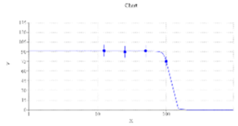	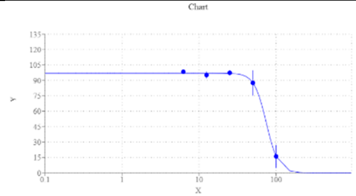
**3f**	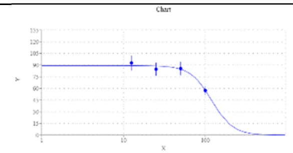	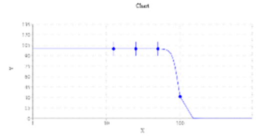	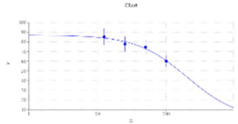	
**4a**	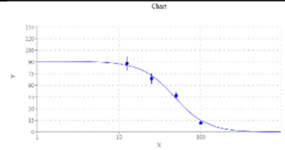	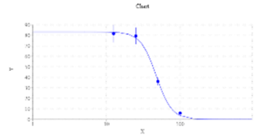	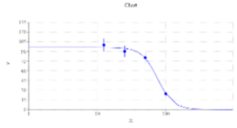	

**4b**	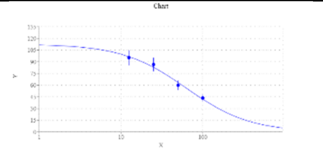	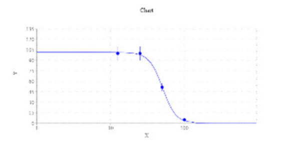	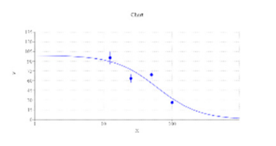	
**4c**	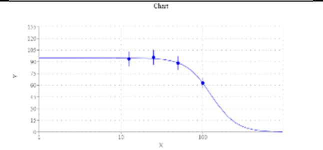	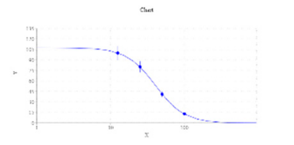	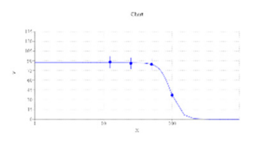	
**4d**	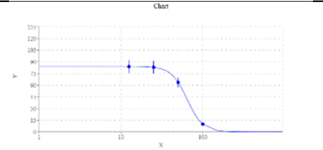	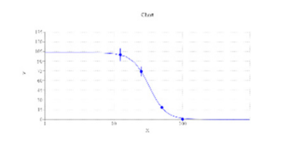	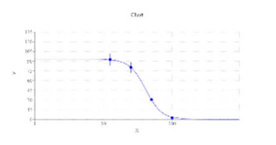	
**4e**	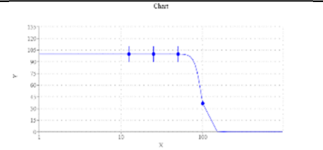	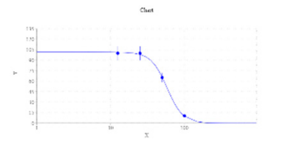	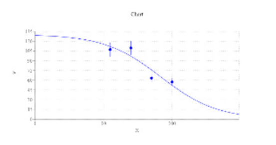	
**6a**	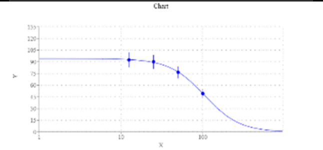	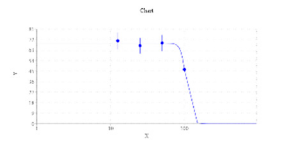	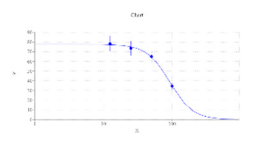	
**6b**	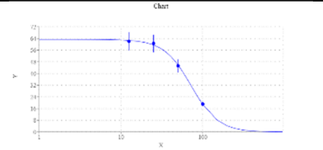	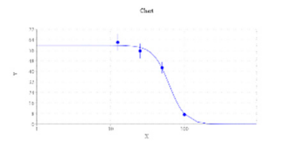	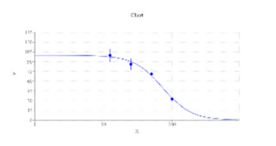	
**6c**	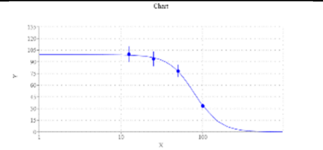	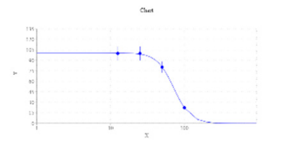	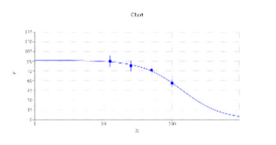	
**8a**	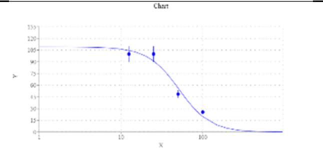	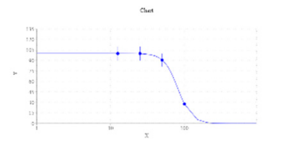	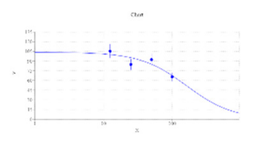	

**8b**	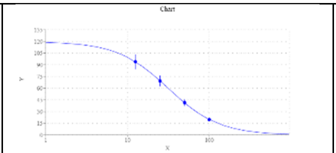	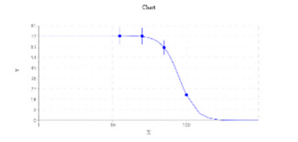	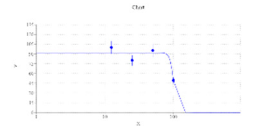	
**8c**	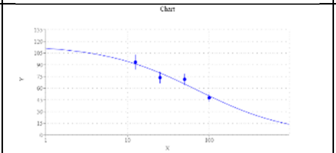	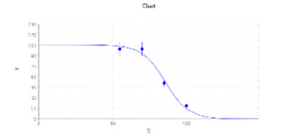	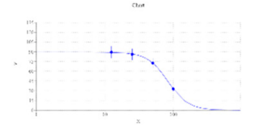	
**8d**	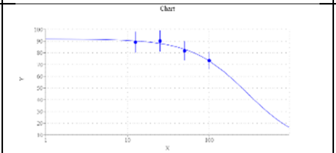	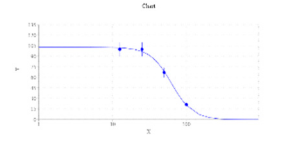	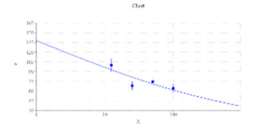	

Figure S62. Cytotoxicity curves of compounds tested for anti-cancer activity
